# A Network Comparison on Safety Profiling of Immune Checkpoint Inhibitors in Advanced Lung Cancer

**DOI:** 10.3389/fimmu.2021.760737

**Published:** 2021-12-03

**Authors:** Yi-Dan Yan, Jiu-Jie Cui, Jie Fu, Ying-Jie Su, Xiao-Yu Chen, Zhi-Chun Gu, Hou-Wen Lin

**Affiliations:** ^1^ Department of Pharmacy, Ren Ji Hospital, Shanghai Jiao Tong University School of Medicine, Shanghai, China; ^2^ Department of Oncology, Ren Ji Hospital, Shanghai Jiao Tong University School of Medicine, Shanghai, China; ^3^ Department of Pharmacy, The People’s Hospital of Guangxi Zhuang Autonomous Region (Guangxi Academy of Medical Sciences), Nanning, China

**Keywords:** immune checkpoint inhibitors, safety, lung cancer, adverse events, network comparison

## Abstract

**Background:**

Immune checkpoint inhibitors (ICIs) have become one of the standard treatment options for advanced lung cancer. However, adverse events (AEs), particularly immune–related AEs (irAEs), caused by these drugs have aroused public attention. The current network meta-analysis (NMA) aimed to compare the risk of AEs across different ICI–based regimens in patients with advanced lung cancer.

**Methods:**

We systematically searched the PubMed, EMBASE, and Cochrane Library databases (from inception to 19 April 2021) for relevant randomized controlled trials (RCTs) that compared two or more treatments, with at least one ICI administered to patients with advanced lung cancer. The primary outcomes were treatment–related AEs and irAEs, including grade 1–5 and grade 3–5. The secondary outcomes were grade 1–5 and grade 3–5 irAEs in specific organs. Both pairwise and network meta-analyses were conducted for chemotherapy, ICI monotherapy, ICI monotherapy + chemotherapy, dual ICIs therapy, and dual ICIs + chemotherapy for all safety outcomes. Node–splitting analyses were performed to test inconsistencies in network. Sensitivity analyses were adopted by restricting phase III RCTs and studies that enrolled patients with non–small cell lung cancer.

**Results:**

Overall, 38 RCTs involving 22,178 patients with advanced lung cancer were enrolled. Both pooled incidence and NMA indicated that treatments containing chemotherapy increased the risk of treatment–related AEs when compared with ICI-based regimens without chemotherapy. As for grade 1–5 irAEs, dual ICIs + chemotherapy was associated with the highest risk of irAEs (probability in ranking first: 50.5%), followed by dual-ICI therapy (probability in ranking second: 47.2%), ICI monotherapy (probability in ranking third: 80.0%), ICI monotherapy + chemotherapy (probability in ranking fourth: 98.0%), and finally chemotherapy (probability in ranking fifth: 100.0%). In grade 3–5 irAEs, subtle differences were observed; when ranked from least safe to safest, the trend was dual ICIs therapy (60.4%), dual ICIs + chemotherapy (42.5%), ICI monotherapy (76.3%), ICI monotherapy + chemotherapy (95.0%), and chemotherapy (100.0%). Furthermore, detailed comparisons between ICI–based options provided irAE profiles based on specific organ/system and severity.

**Conclusions:**

In consideration of overall immune–related safety profiles, ICI monotherapy + chemotherapy might be a better choice among ICI–based treatments for advanced lung cancer. The safety profiles of ICI–based treatments are various by specific irAEs and their severity.

**Systematic Review Registration:**

https://www.crd.york.ac.uk/prospero, identifier CRD42021268650

## Introduction

Lung cancer remains the leading cause of global cancer mortality, with approximately 1.8 million deaths annually (18% of the total cancer deaths) (1). Over the past decades, platinum–based chemotherapy has become the cornerstone for managing advanced lung cancer; however, its use is of concern due to inevitable resistance and intolerable adverse events (AEs) in these fragile patients (2, 3). Recently, immunotherapy has revolutionised treatment approaches for advanced lung cancer by making longer survival times a reality (4). Unlike traditional therapy (chemotherapy and targeted therapy), immune checkpoint Inhibitor (ICI) therapies use monoclonal antibodies to inhibit the expression of proteins [cytotoxic T lymphocyte associated antigen (CTLA4), programmed death-1 receptor (PD-1), and its ligand (PD-L1)], thereby boosting T–cell activation against cancer (5). To date, a series of ICIs [pembrolizumab, nivolumab, ipilimumab, atezolizumab, and durvalumab registered by the U.S. Food and Drug Administration (FDA); camrelizumab, sintilimab, and tislelizumab approved by the National Medical Products Administration (NMPA)] have been successfully introduced for use in patients with advanced lung cancer. Recently, ICI monotherapy with or without chemotherapy, dual ICIs combination, even dual ICIs combined with chemotherapy have been clinically applied as standard first–line treatment options for advanced lung cancer.

With a dramatic increase in the availability of ICI drugs and their superior efficacy, a substantial proportion of patients with lung cancer are administered these agents. Nonetheless, concerns regarding unique treatment–specific toxicities owing to their pharmacological mechanisms, namely immune–related AEs (irAEs), associated with ICI regimens are growing (6). IrAEs are unintended effects following the activation of the immune system by ICI–mediation and can occur in any organ or system, including the gastrointestinal tract, lungs, endocrine, skin, heart, renal, liver, and muscles (7). In published randomised controlled trials (RCTs), patients administered ICIs experienced fewer AEs than those undergoing chemotherapy, while the incidence of any irAEs seemed to be distinctly higher in the ICI group (8). Without timely identification and proper management, irAEs can become severe complications, resulting in treatment discontinuation or failure, and even death (9, 10).

Previous meta-analyses have examined the risks of irAEs associated with ICI therapy; however, most of them mainly involved patients with all types of cancers (11–13). In addition, these studies did not explicitly examine the risk of individual irAEs across different ICI regimens, which may vary according to cancer type. Recently, one network meta-analysis (NMA) (14) and six traditional meta-analyses (14–19) addressed this issue in patients with lung cancer. However, they focused on one or two specific irAEs; therefore, the entire toxicity spectrum in these patients is yet to be described. Since, to the best our knowledge, head–to–head comparisons among ICI regimens are lacking in current literature, an indirect analysis could be performed to obtain comparative results and rank all possible treatments (20). In the present study, we conducted an NMA using up-to-date data from ICI–treated patients with advanced lung cancer to compare the risk of developing AEs during or following various treatment strategies.

## Methods

### Literature Search

This NMA was conducted according to the priori established protocol (PROSPERO: CRD42021268650; https://www.crd.york.ac.uk/prospero/#searchadvanced), and reported in accordance with the PRISMA (Preferred Reporting Items for Systematic Reviews and Meta-analyses) guidelines (21) and its extension statement for NMA (**Table S1**). The PubMed, EMBASE, and Cochrane Library databases were systematically searched from inception to 19 April 2021 with language restricted to English. The search terms and their combinations used in the search strategies are shown in **Table S2**. We also identified potential studies listed as references in the retrieved articles and searched unpublished data from the ClinicalTrials.gov website.

### Study Selection

The eligibility criteria for published studies were as follows: head-to-head phase II and III RCTs comparing two or more treatments, including at least one ICI drug in patients with advanced lung cancer. Studies published only in the form of conference abstracts, posters, and presentations of ongoing RCTs were excluded. If several studies were derived from the same trial, the study that reported comprehensive safety data was involved. Two authors (Y.Y. and J.C.) independently screened all titles and abstracts, and further assessed potentially eligible full text based on the aforementioned criteria.

### Study Outcomes and Data Extraction

The primary outcomes of this study were overall safety outcomes, viz. treatment–related adverse events and immune-related adverse events as defined in each study (**Table S3**), including grade 1–5 and grade 3–5, respectively. The secondary outcomes were grade 1–5 and grade 3–5 irAEs in specific organ systems, including the gastrointestinal system (colitis and diarrhea), pulmonary system (pneumonitis), endocrine system (hyperthyroidism, hypothyroidism, thyroiditis, hypophysitis, and diabetes), skin (pruritus, rash, and severe skin reaction), and others (myocarditis, nephritis, hepatitis, myositis, and hypersensitivity/infusion reaction). Grading of AEs was reported according to the Common Terminology Criteria for Adverse Events (CTCAE), as reported in each study. Two authors (Y.Y. and J.C.) used a pre-designed form to extract the following data: study characteristics (study ID and publication year, NCT number, cancer type, study design, arms, treatment regimens, number of patients, follow–up time, and version of CTCAE), demographics and clinical characteristics (age, sex, PS score, brain/CNS metastasis, liver metastasis, bone metastasis, current/former smoker, prior surgery, and prior radiotherapy), and data on the aforementioned outcomes. The above information was extracted from the main text and **Supplementary Materials**, and only accessible data were analysed.

### Quality Assessment

The methodological quality of the included trials was assessed using the Cochrane Collaboration Risk of Bias Tool (22). Low, moderate, or high risk of bias was assigned to each citation within the following five aspects: random sequence generation, allocation concealment, masking, assessment of outcomes, and selective reporting. Disagreements during study selection, data extraction, and quality assessment processes were resolved by consensus following a consultation with the corresponding investigator (Z.G.).

### Statistical Analyses

To illustrate the direct and indirect comparisons among the treatments, a plot of the network geometry was generated. A pairwise meta-analysis of head-to-head comparisons was conducted to make direct estimates. Results were reported as relative risks (RRs) with 95% confidence intervals (95% CIs) using a random–effect model. Statistical heterogeneity was assessed using the *I^2^*-test, with a value > 50% representing considerable heterogeneity (23). A sensitivity analysis was performed to assess the robustness of the results by sequentially eliminating each study from the pool (24, 25). Furthermore, meta-regression analyses were performed to explore the influence of potential factors on patient outcomes (26). When a single analysis involved > 10 studies, publication bias was evaluated using funnel plots, as well as Egger’s and Begg’s tests (27). For outcomes with potential publication bias, the trim and fill method were used to estimate the number of missing studies and to provide an estimated intervention effect to perform adjustment for publication bias. In the network comparison among treatment regimens, chemotherapy was used as the reference comparator. Random effects and consistency models were used to calculate RRs and their 95% CIs; these models are thought to be the most conservative approach to dealing with between–study heterogeneity. Cumulative probabilities were used to provide a hierarchy of the treatments. According to the cumulative probabilities, treatment regimens were ranked from the worst (i.e., associated with the highest risk of AEs) to the best (i.e., associated with the lowest risk of AEs) (28). Transitivity was appraised in consistency and coherence: first, interaction analyses were used to assess the comparability between the consistency and inconsistency models; second, node–splitting analyses were performed to test coherence in the network. To further ensure the robustness of the findings, sensitivity analyses were adopted by restricting the following factors: phase III RCTs and studies that enrolled patients with non–small cell lung cancer (NSCLC). The incidences of grade 1–5 and grade 3–5 AEs were also pooled using meta-analysis (29). All data were analysed by using STATA version13.0 (Statacorp, College Station, Texas, United States), with p values < 0.05 indicating a statistically significant difference.

## Results

### Study Selection and Characteristics

**Figure 1:**
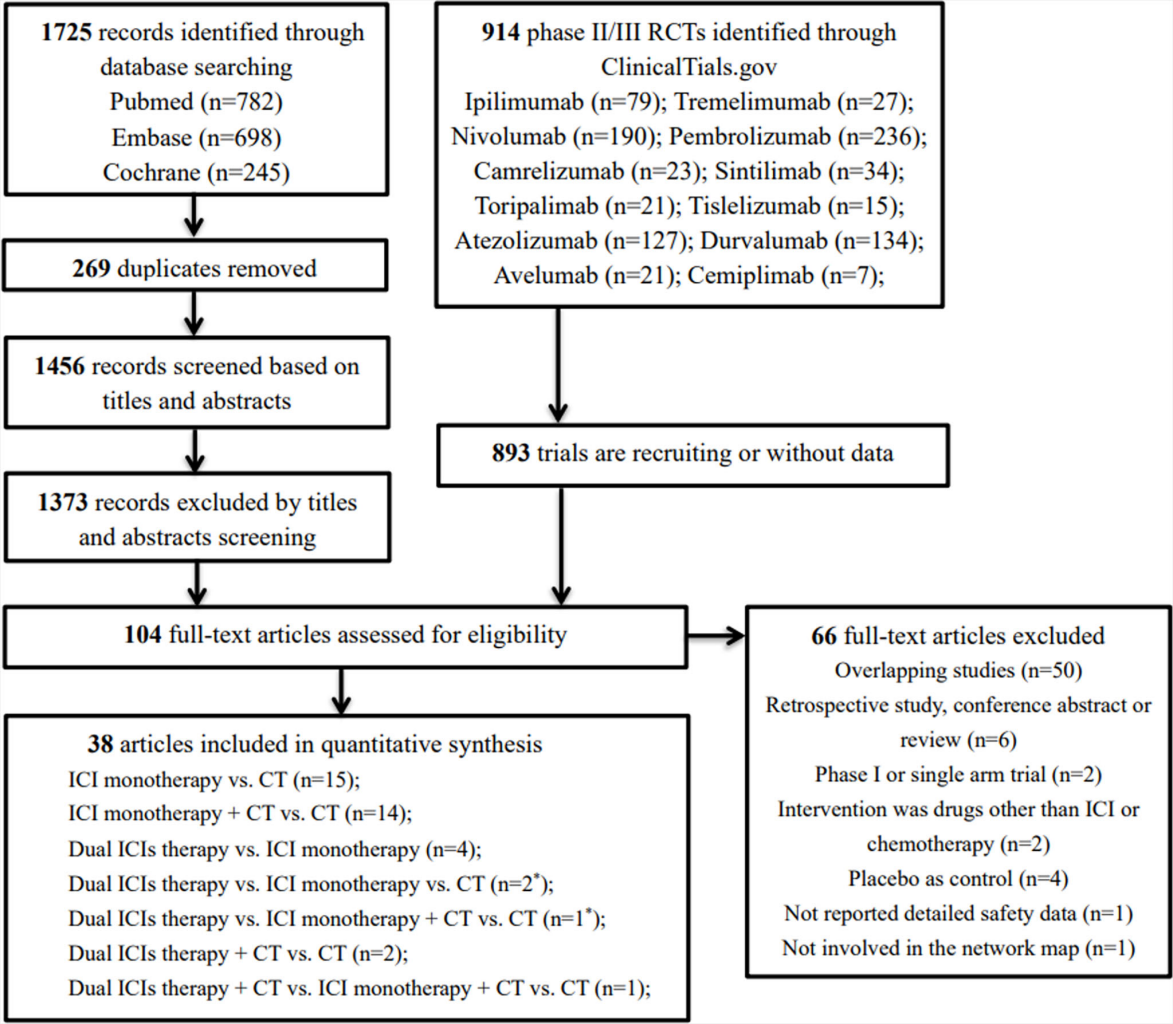
Flow diagram for the selection of eligible studies. ICI, immune checkpoint inhibitor; CT, chemotherapy; n, number; *, one study involved two groups, dual ICIs therapy *vs.* ICI monotherapy *vs.* CT (group A) and dual ICIs therapy *vs.* ICI monotherapy + CT *vs.* CT (group B).

**Figure 2:**
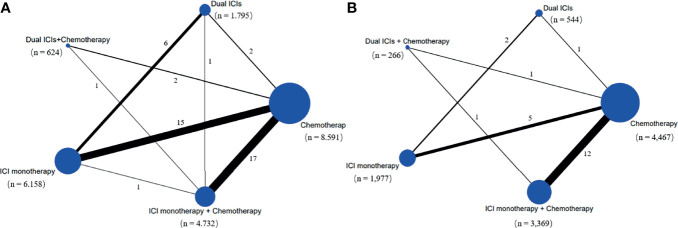
Network map of comparisons based on five treatments in grade 1-5 adverse events **(A)** and grade 1-5 immune-related adverse events **(B)**. Each circular node represents a type of treatment. The node size is proportional to the total number of patients administering a treatment (in parentheses). Each line represents a type of head-to-head comparison. The width of lines is proportional to the total number of studies comparing the connected treatments. ICI, immune checkpoint inhibitor; n, number.

Our initial search yielded 1,725 records from databases and 914 records from the ClinicalTrials.gov platform; 2,535 records were excluded following screening of titles and abstracts. The remaining 104 full–text articles were reviewed, and 66 articles were excluded for reasons depicted in **Figure 1** and **Table S4**. Given that only one trial (IMpower150) involved groups of ICI + targeted + chemotherapy and ICI + targeted therapy, which had no head-to-head comparison with other five treatments, it was excluded in our network map. Finally, 38 studies (30–67) met the inclusion criteria, and their characteristics are listed in **Table 1**. Of these 38 studies, 30 were phase III trials, six were phase II trials, one was a phase I/II trial, and one was a phase II/III trial. As for the indication, 28 RCTs involved patients with NSCLC, and the remaining nine RCTs included patients with small cell lung cancer (SCLC). The sample sizes ranged from 73–1,739 participants, and the median follow–up time varied from 6.6–30.2 months across trials. As shown in the network map (**Figure 2**), a total of 22,178 patients with advanced lung cancer were included in five treatment regimens (8,768, 6,057, 4,917, 1,807, and 629 patients received chemotherapy, ICI monotherapy, ICI monotherapy + chemotherapy, dual ICIs therapy, and dual ICIs + chemotherapy, respectively). Detailed patient demographics and clinical characteristics are summarised in **Table S5**. The median age of patients was ranged from 50.1–66 years, and the proportion of males was 68.9%. Most of the patients were current or former smokers (84.4%), with a PS score of 0–1 (98.8%). Overall, 16.7% of the patients were reported to have metastasis (brain, liver, or bone) at baseline, while 3.4% and 4.8% of them were previously reported to have undergone surgery and radiotherapy, respectively.

**Table 1:** Baseline characteristics of 38 studies.

Study, year	NCT number	Cancer type	Phase	Line of treatment	Arms	Treatment	Numbers	Median follow-up time (months)	CTCAE version
CA184-041, 2012	NCT00527735	NSCLC	II	1	1	Ipi 10mg/kg q3w+PC	138	NR	3.0
					2	PC	66	NR	
CA184-041, 2013	NCT00527735	SCLC	II	1	1	Ipi 10mg/kg q3w+PC	85	NR	3.0
					2	PC	45	NR	
CheckMate 057,2015	NCT01673867	Nonsquamous NSCLC	III	2	1	Niv 3mg/kg q2w	292	Min 13.2	4.0
					2	Docetaxel	290	Min 13.2	
CheckMate 017, 2015	NCT01642004	Squamous NSCLC	III	2	1	Niv 3mg/kg q2w	135	Min 11.0	4.0
					2	Docetaxel	137	Min 11.0	
CheckMate 032, 2016	NCT01928394	SCLC	I/II	≥2	1	Niv 3mg/kg q2w	98	6.6	4.0
					2	Niv 1mg/kg+Ipi 3mg/kg q3w	61	10.6	
					3	Niv 3mg/kg+Ipi 1mg/kg q3w	54	12.0	
POPLAR, 2016	NCT01903993	NSCLC	II	≥2	1	Ate 1200mg q3w	144	14.8	4.0
					2	Docetaxel	143	15.7	
KEYNOTE-010, 2016	NCT01905657	NSCLC	II/III	≥2	1	Pem 2mg/kg q3w	344	13.1	4.0
					2	Pem 10mg/kg q3w	346	13.1	
					3	Docetaxel	343	13.1	
KEYNOTE-021, 2016	NCT02039674	Nonsquamous NSCLC	II	1	1	Pem200mg q3w+AC	60	10.6	4.0
					2	AC	63	10.6	
CA184-156, 2016	NCT01450761	SCLC	III	1	1	Ipi 10mg/kg q3w+EP	478	10.5	3.0
					2	EP	476	10.2	
KEYNOTE 024, 2016	NCT02142738	NSCLC	III	1	1	Pem 200mg q3w	154	25.2	4.0
					2	P-based chemotherapy	151	25.2	
CheckMate 026, 2017	NCT02041533	NSCLC	III	1	1	Niv 3mg/kg q2w	271	13.5	4.0
					2	P-based chemotherapy	270	13.5	
Study 104, 2017	NCT01285609	Squamous NSCLC	III	1	1	Ipi 10 mg/kg q3w+PC	388	12.5	3.0
					2	PC	361	11.8	
OAK, 2017	NCT02008227	NSCLC	III	≥2	1	Ate 1200mg q3w	425	21	4.0
					2	Docetaxel	425	21	
JAVELIN Lung 200, 2018	NCT02395172	NSCLC	III	≥2	1	Ave 10 mg/kg q2w	396	18.9	4.0
					2	Docetaxel	396	17.8	
KEYNOTE-189, 2018	NCT02578680	Nonsquamous NSCLC	III	1	1	Pem 200mg q3w+AP	410	10.5	4.0
					2	AP	206	10.5	
IMpower133, 2018	NCT02763579	SCLC	III	1	1	Ate 1200mg q3w+EC	201	13.9	4.0
					2	EC	202	13.9	
KEYNOTE-407, 2018	NCT02775435	Squamous NSCLC	III	1	1	Pem 200mg q3w+PC/nPC	278	7.8	4.03
					2	PC/nPC	281	7.8	
CheckMate 227, 2019	NCT02477826	NSCLC	III	1	GroupA-1	Niv 3mg/kg q2w+Ipi 1mg/kg q6w	396	Min 29.3	4.0
					GroupA-2	Niv 240mg q2w	396	Min 29.3	
					GroupA-3	P-based chemotherapy	397	Min 29.3	
					GroupB-1	Niv 3mg/kg q2w+Ipi 1mg/kg q6w	187	Min 29.3	
					GroupB-2	Niv 360mg +P-based chemotherapy q3w	177	Min 29.3	
					GroupB-3	P-based chemotherapy	186	Min 29.3	
KEYNOTE-042, 2019	NCT02220894	NSCLC	III	1	1	Pem 200mg q3w	637	12.8	4.0
					2	P-based chemotherapy	637	12.8	
IFCT-1603, 2019	NCT03059667	SCLC	II	2	1	Ate 1200mg q3w	49	13.7	4.0
					2	EC or topotecan	24	13.7	
IMpower130, 2019	NCT02367781	Nonsquamous NSCLC	III	1	1	Ate 1200mg q3w+nPC	483	18.5	4.0
					2	nPC	240	19.2	
CheckMate 078, 2019	NCT02613507	NSCLC	III	2	1	Niv 3mg/kg q2w	338	10.4	4.0
					2	Docetaxel	166	8.8	
PROLUNG, 2020	NCT02574598	NSCLC	II	2	1	Pem 200mg q3w+Docetaxel	40	8.9	NR
					2	Docetaxel	38	7.9	
IMpower110, 2020	NCT02409342	NSCLC	III	1	1	Ate 1200mg q3w	277	13.4	4.0
					2	P-based chemotherapy	277	13.4	
IMpower131, 2020	NCT02367794	Squamous NSCLC	III	1	1	Ate 1200mg q3w+PC	338	NR	4.0
					2	Ate 1200mg q3w+nPC	343	26.8	
					3	nPC	340	24.8	
IMpower132, 2020	NCT02657434	Nonsquamous NSCLC	III	1	1	Ate 1200mg q3w+AP	292	28.4	4.0
					2	AP	286	28.4	
ARCTIC, 2020	NCT02352948	NSCLC	III	≥3	1	Dur 20mg/kg+Tre 1mg/kg q4w	174	9.1	NR
					2	Dur 10mg/kg q2w	117	9.1	
					3	Tre 10mg/kg q4w	60	9.1	
MYSTIC, 2020	NCT02453282	NSCLC	III	1	1	Dur 20mg/kg q4w	374	30.2	NR
					2	Dur 20mg/kg q4w+Tre 1mg/kg q4w	372	30.2	
					3	P-based chemotherapy	372	30.2	
KEYNOTE-604, 2020	NCT03066778	SCLC	III	1	1	Pem 200mg q3w+EP	228	21.6	4.0
					2	EP	225	21.6	
ORIENT-11, 2020	NCT03607539	Nonsquamous NSCLC	III	1	1	Sin 200mg q3w+AP	266	8.9	4.03
					2	AP	131	8.9	
CameL, 2020	NCT03134872	Nonsquamous NSCLC	III	1	1	Cam 200mg q3w+AC	205	19.3	4.03
					2	AC	207	19.3	
CASPIAN, 2021	NCT03043872	SCLC	III	1	1	Dur 1500mg q3w+Tre 75mg q3w+EP	268	25.1	4.03
					2	Dur 1500mg q3w+EP	268	25.1	
					3	EP	269	25.1	
CheckMate 451, 2021	NCT02538666	SCLC	III	2	1	Niv 1mg/kg+Ipi 3mg/kg q3w	279	8.4	4.0
					2	Niv 240mg q2w	280	9.9	
CheckMate 9LA, 2021	NCT03215706	NSCLC	III	1	1	Niv 360mg q3w+Ipi 1mg/kg q6w+P-based chemotherapy	361	9.7	4.0
					2	P-based chemotherapy	358	9.7	
CheckMate 331, 2021	NCT02481830	SCLC	III	2	1	Niv 240mg q2w	284	7.0	4.0
					2	Topotecan or amrubicin	285	7.6	
KEYNOTE-598, 2021	NCT03302234	NSCLC	III	1	1	Pem 200mg q3w+Ipi 1mg/kg q6w	284	20.6	4.0
					2	Pem 200mg q3w	284	20.6	
EMPOWER-Lung 1, 2021	NCT03088540	NSCLC	III	1	1	Cem 350mg q3w	356	13.1	4.03
					2	P-based chemotherapy	354	13.1	
RATIONALE 307, 2021	NCT03594747	Squamous NSCLC	III	1	1	Tis 200mg q3w+PC	120	8.6	5.0
					2	Tis 200mg q3w+nPC	119	8.6	
					3	PC	121	8.6	

CTCAE, Common Terminology Criteria for Adverse Events; NSCLC, non–small cell lung cancer; SCLC, small cell lung cancer; mg, milligram; kg, kilogram; q3w, receive agents every three weeks; Ipi, ipilimumab; PC, paclitaxel + carboplatin; Niv, nivolumab; Ate, atezolizumab; Pem, pembrolizumab; AC, pemetrexed + carboplatin; EP, etoposide + cisplatin; P-based chemotherapy, platinum–based chemotherapy; Ave, avelumab; AP, pemetrexed + platinum; EC, etoposide + carboplatin; nPC, nanoparticle albumin-bound paclitaxel + carboplatin; Dur, durvalumab; Tre, tremelimumab; Sin, sintilimab; Cem, cemiplimab; Tis, tislelizumab; Min, minimum; NR, not report.

### Risk-of-Bias Assessment

The included RCTs satisfied three tool items, viz. random sequence generation, incomplete outcome data, and selective reporting. Twenty–seven open–label trials did not meet the item of allocation concealment or blinding of participants and personnel, resulting in a high risk of bias in the study assessment. Overall, 27 studies exhibited a high risk of bias, and 11 studies were considered to have a low risk of bias. The details of the quality assessment are presented in **Table S6**.

### Pairwise Meta-Analysis Based on Head-to-Head Comparisons

Direct comparisons were conducted to assess safety profiles among five treatments options (**Table S7**). Compared with conventional chemotherapy, three treatment regimens (ICI monotherapy, dual ICIs, and dual ICIs + chemotherapy) had a similar risk for grade 1–5 AEs, except for ICI monotherapy + chemotherapy (RR: 1.03, 95% CI: 1.01–1.04). Meanwhile, dual ICIs therapy appeared to have a higher risk for grade 1–5 AEs than ICI monotherapy (RR: 1.17, 95% CI: 1.04–1.31). Regarding grade 3–5 AEs, chemotherapy showed a noticeably higher risk compared with ICI monotherapy (RR: 0.33, 95% CI: 0.27–0.40 for ICI monotherapy *vs.* chemotherapy), while there was a reduced risk when compared with dual ICIs + chemotherapy (RR: 0.33, 95% CI: 0.27–0.40 for dual ICIs + chemotherapy *vs.* chemotherapy). Other results were consistent with those observed in grade 1–5 AEs.

In terms of irAEs, the use of ICI monotherapy (RR: 4.06, 95% CI: 2.75–5.98 for grade 1–5; RR: 5.75, 95% CI: 3.50–9.43 for grade 3–5) or ICI monotherapy + chemotherapy (RR: 2.02, 95% CI: 1.63–2.52 for grade 1–5; RR: 2.93, 95% CI: 1.98–4.34 for grade 3–5) was associated with a significantly higher risk of developing these manifestations than chemotherapy. Dual ICIs therapy had a similar risk of grade 1–5 irAEs (RR: 1.48, 95% CI: 0.77–2.84) but a superior risk of grade 3–5 irAEs (RR: 2.12, 95% CI: 1.29–3.48) compared with ICI monotherapy. Detailed data in specific irAEs are listed in **Table S7**. Briefly, results from organ–specific irAEs, including colitis, pneumonitis, hyper/hypothyroidism, hepatitis, and rash, were comparable with those observed in overall irAEs. Regarding heterogeneity of pairwise meta-analysis comparisons, relatively high heterogeneity was found in primary outcomes (*I^2^*: 38.2%–95.0%), except for two pairs (dual ICIs + chemotherapy *vs.* chemotherapy for grade 3–5 AEs and ICI monotherapy *vs.* chemotherapy for grade 3–5 irAEs). Overall, the general heterogeneity in individual irAEs was low to moderate.

Sensitivity analyses were conducted by sequentially removing each study. Following this, the pooled results were in line with the set primacy safety outcomes (**Table S8**). In addition, meta-regression analysis failed to detect any potential confounding factors affecting the primacy outcomes (**Table S9**). A visual inspection of the funnel plots and Begg’s test showed relative symmetry, except for grade 3–5 AE in comparison of ICI monotherapy *vs.* chemotherapy (*P* = 0.018) (**Figure S1** and **Table S10**). However, *P* values of Egger’s test in several outcomes were < 0.05, suggesting that publication bias existed in this study (**Table S10**). The trim and fill method were adopted to mitigate publication bias, and the outcomes were consistent with our primary results (*P* for interaction > 0.05) (**Table S10**).

### Network Meta-Analysis for Overall Safety

**Figure 3:**
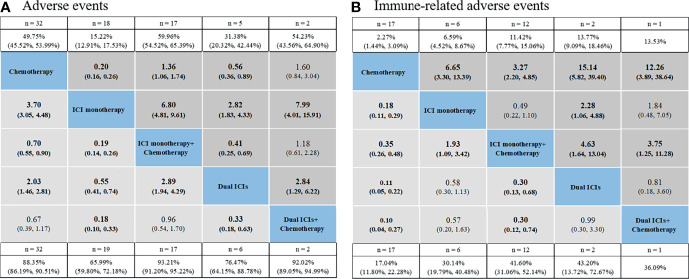
Safety profiles based on adverse events **(A)** and immune-related adverse events **(B)**. Pooled incidences and 95% confidence intervals of grade 1–5 events for each treatment are at bottom and that of grade 3–5 events are at top of the figure. Each cell of the safety profiles contains the pooled relative risks and 95% confidence intervals for grade 1–5 (light gray cell) and grade 3–5 (dark gray cell) events; significant results are in bold. The pooled relative risks and 95% confidence intervals indicate the results of the top treatment compared with the bottom treatment. ICI, immune checkpoint inhibitor; n, number.

The pooled incidence for five treatments showed the following rankings: ICI monotherapy + chemotherapy had the highest incidence of AEs (93.21% for grade 1–5, 58.48% for grade 3–5), followed by dual ICIs + chemotherapy (92.02%, 54.23%), chemotherapy (88.35%, 49.75%), dual ICIs therapy (76.47%, 31.38%), ICI monotherapy (65.99%, 15.22%) (**Figure 3A** and **Table S11**). Established NMA based on the consistency model indicated that ICI monotherapy had the lowest risk of causing grade 1–5 AEs compared with chemotherapy (RR: 3.7, 95% CI: 3.05–4.48 for chemotherapy *vs.* ICI monotherapy), ICI monotherapy + chemotherapy (RR: 0.19, 95% CI: 0.14–0.26), dual ICIs therapy (RR: 0.55, 95% CI: 0.41–0.74), as well as dual ICIs + chemotherapy (RR: 0.18, 95% CI: 0.10–0.33) (**Figure 3A**). Then was dual ICIs therapy (RR: 2.03, 95% CI: 1.46–2.81 for chemotherapy *vs.* dual ICIs; RR: 2.89, 95% CI: 1.94–4.29 for ICI monotherapy + chemotherapy *vs.* dual ICIs; RR: 0.33, 95% CI: 0.18–0.63 for dual ICIs *vs.* dual ICIs + chemotherapy). ICI monotherapy + chemotherapy showed a higher risk of causing grade 1–5 AEs than chemotherapy (RR: 0.70, 95% CI: 0.55–0.90 for chemotherapy *vs.* ICI + chemotherapy), while no significant difference was seen when compared with that of the ICIs + chemotherapy group (RR: 0.96, 95% CI: 0.54–1.70). Similar results were observed in grade 3–5 AEs. The ranking probabilities based on the five treatment groups are also depicted in **Figure 3A**. The rankings of grade 3–5 AEs were in line with those of grade 1–5 AEs, ranging from least safe to safest as follows: ICIs + chemotherapy (probability: 56.2% for grade 1–5; 68.5% for grade 3–5), ICI monotherapy + chemotherapy (56.0%; 67.9%), chemotherapy (91.9%; 91.1%), dual ICIs therapy (100.0%; 98.9%), and ICI monotherapy (100.0%; 100.0%) (**Table 2** and, **Table S12**).

**Table 2:** Rankings based on overall AE and irAE.

	1st	2nd	3rd	4th	5th
**Grade 1-5 AE**	Dual ICIs + CT (56.2)	ICI monotherapy + CT (56.0)	Chemotherapy (91.9)	Dual ICIs (100.0)	ICI monotherapy (100.0)
**Grade 3-5 AE**	Dual ICIs + CT (68.5)	ICI monotherapy + CT (67.9)	Chemotherapy (91.1)	Dual ICIs (98.9)	ICI monotherapy (100.0)
**Grade 1-5 irAE**	Dual ICIs + CT (50.5)	Dual ICIs (47.2)	ICI monotherapy (80.0)	ICI monotherapy + CT (98.0)	Chemotherapy (100.0)
**Grade 3-5 irAE**	Dual ICIs (60.4)	Dual ICIs + CT (42.5)	ICI monotherapy (76.3)	ICI monotherapy + CT (95.0)	Chemotherapy (100.0)

The value in each parenthesis represents the probability of risk to rank (%). ICI, immune checkpoint inhibitor; CT, chemotherapy; AE, adverse event; irAE, immune-related adverse event.

As for overall irAEs, the pooled incidences for chemotherapy, ICI monotherapy, ICI monotherapy + chemotherapy, dual ICIs, and dual ICIs + chemotherapy were 17.04%, 30.14%, 41.60%, 43.20%, and 36.09% for grade 1–5 irAEs, and 2.27%, 6.59%, 11.42%, 13.77%, and 13.53% for grade 3–5 irAEs, respectively. Based on NMA, the safety profiles (**Figure 3B**) of the five treatment choices indicated an extremely decreased risk of irAEs favoring chemotherapy over the other four treatment strategies for both grade 1–5 (RR: 0.18, 95% CI: 0.11–0.29 for chemotherapy *vs.* ICI monotherapy; RR: 0.35, 95% CI: 0.26–0.48 for chemotherapy *vs.* ICI monotherapy + chemotherapy; RR: 0.11, 95% CI: 0.05–0.22 for chemotherapy *vs.* dual ICI therapy; RR: 0.10, 95% CI: 0.04–0.27 for chemotherapy *vs.* dual ICIs + chemotherapy) and grade 3–5 events (RR: 6.65, 95% CI: 3.30–13.39 for ICI monotherapy *vs.* chemotherapy; RR: 3.27, 95% CI: 2.20–4.85 for ICI monotherapy + chemotherapy *vs.* chemotherapy; RR: 15.14, 95% CI: 5.82–39.40 for dual ICI therapy *vs.* chemotherapy; RR: 12.26, 95% CI: 3.89–38.64 for dual ICIs + chemotherapy *vs.* chemotherapy). Among ICI therapeutic schedules, ICI monotherapy + chemotherapy seemed safer than ICI monotherapy (RR: 1.93, 95% CI: 1.09–3.42 for ICI monotherapy *vs.* ICI monotherapy + chemotherapy in grade 1–5 irAEs; RR: 0.49, 95% CI: 0.22–1.10 in grade 3–5 irAEs), dual ICIs therapy (RR: 0.30, 95% CI: 0.13–0.68 in grade 1–5 irAEs; RR: 4.63, 95% CI: 1.64–13.04 for dual ICIs *vs.* ICI monotherapy + chemotherapy in grade 3–5 irAEs), and dual ICIs + chemotherapy (RR: 0.30, 95% CI: 0.12–0.74 in grade 1–5 irAEs; RR: 3.75, 95% CI: 1.25–11.28 for dual ICIs + chemotherapy *vs.* ICI monotherapy + chemotherapy in grade 3–5 irAEs), with one ICI being observed to be safer than two ICIs combination with regards to grade 3–5 irAEs (RR: 2.28, 95% CI: 1.06–4.48 for dual ICIs *vs.* ICI monotherapy). In aspect of safety ranking, dual ICIs + chemotherapy was associated with the worst ranking for grade 1–5 irAEs (probability: 50.5%), followed by dual ICIs (47.2%), ICI monotherapy (80.0%), ICI monotherapy + chemotherapy (98.0%), and finally chemotherapy (100.0%). The risk of experiencing grade 3–5 irAEs was ranked from high to low as follows: dual ICIs (60.4%), dual ICIs + chemotherapy (42.5%), ICI monotherapy (76.3%), ICI monotherapy + chemotherapy (95.0%), and chemotherapy (100.0%) (**Table 2**, **Table S12**).

The results pooled *via* the inconsistency model had a generally satisfactory fit compared with those calculated by the consistency model, except for minor comparisons based on chemotherapy (**Table S13**). Likewise, a less significant inconsistency was observed following the node–splitting analysis (**Table S14**).

### Network Meta-Analysis for Specific IrAEs

**Figure 4:**
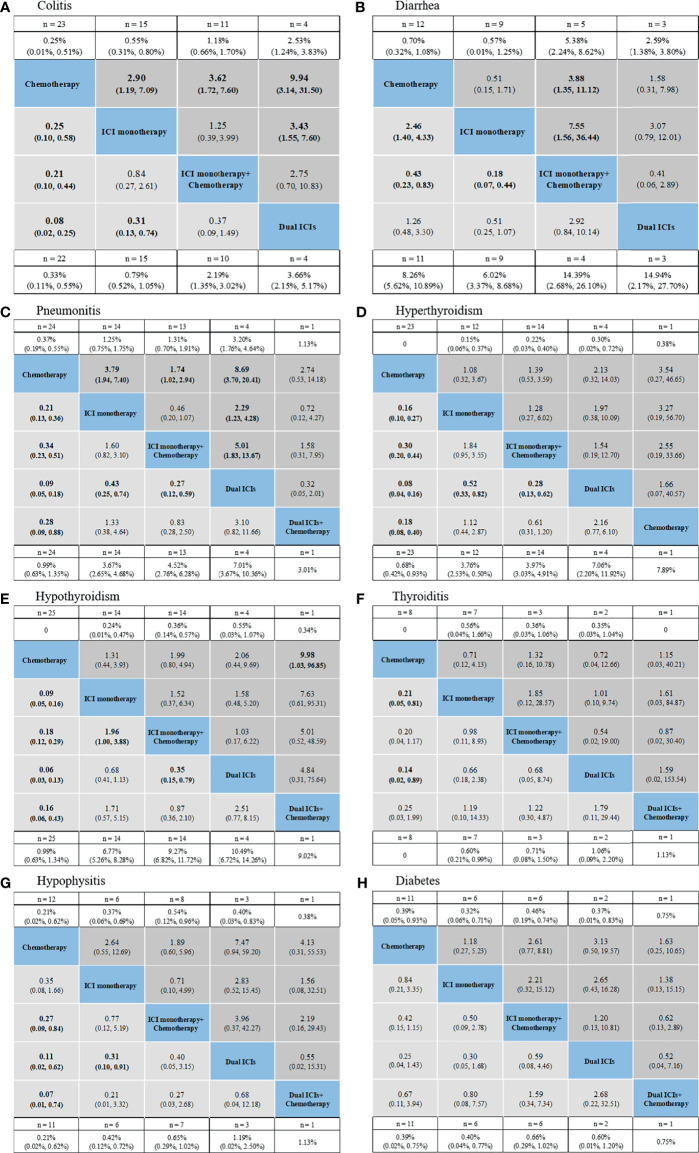
Safety profiles based on specific organs: colitis **(A)**, diarrhea **(B)**, pneumonitis **(C)**, hyperthyroidism **(D)**, hypothyroidism **(E)**, thyroiditis **(F)**, hypophysitis **(G)**, diabetes **(H)**. Pooled incidences and 95% confidence intervals of grade 1–5 events for each treatment are at bottom and that of grade 3–5 events are at top of the figure. Each cell of the safety profiles contains the pooled relative risks and 95% confidence intervals for grade 1–5 (light gray cell) and grade 3–5 (dark gray cell) events; significant results are in bold. The pooled relative risks and 95% confidence intervals indicate the results of the top treatment compared with the bottom treatment. ICI, immune checkpoint inhibitor; n, number.

**Figure 5:**
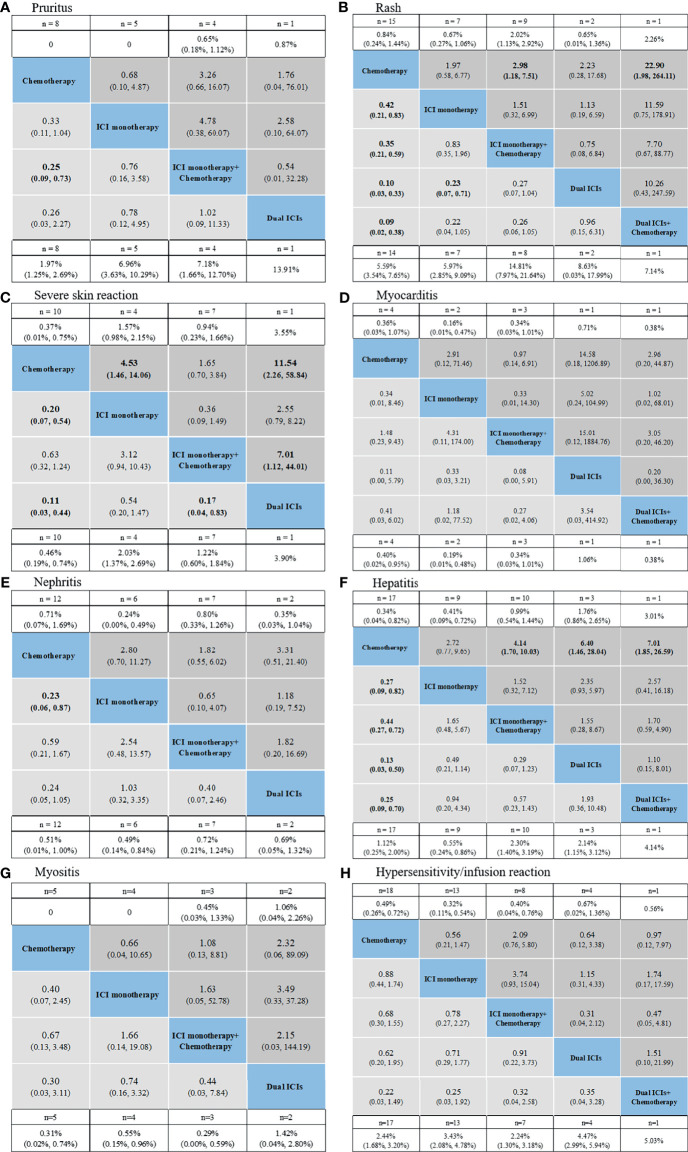
Safety profiles based on specific organs: pruritus **(A)**, rash **(B)**, severe skin reaction **(C)**, myocarditis **(D)**, nephritis **(E)**, hepatitis **(F)**, myositis **(G)**, hypersensitivity/infusion reaction **(H)**. Pooled incidences and 95% confidence intervals of grade 1–5 events for each treatment are at bottom and that of grade 3–5 events are at top of the figure. Each cell of the safety profiles contains the pooled relative risks and 95% confidence intervals for grade 1–5 (light gray cell) and grade 3–5 (dark gray cell) events; significant results are in bold. The pooled relative risks and 95% confidence intervals indicate the results of the top treatment compared with the bottom treatment. ICI, immune checkpoint inhibitor; n, number.

NMAs and ranking probabilities for different treatment strategies in subgroups of irAEs are depicted in **Figures 4**, **5** and **Table 3**. Although the results for individual irAEs varied by organ system and severity, traditional chemotherapy presented the lowest risk in majority of irAEs.

**Table 3:** Toxicity spectra and rankings based on each specific irAEs.

	1st	2nd	3rd	4th	5th
**Gastrointestinal irAE**					
** sColitis G1-5**	Dual ICIs (91.6)	ICI monotherapy + CT (54.0)	ICI monotherapy (61.8)	Chemotherapy (99.9)	–
** Colitis G3-5**	Dual ICIs (92.3)	ICI monotherapy + CT (57.2)	ICI monotherapy (63.7)	Chemotherapy (98.8)	–
** Diarrhea G1-5**	ICI monotherapy + CT (94.9)	Chemotherapy (68.1)	Dual ICIs (64.5)	ICI monotherapy (96.4)	–
** Diarrhea G3-5**	ICI monotherapy + CT (81.6)	Dual ICIs (52.4)	Chemotherapy (57.9)	ICI monotherapy (82.0)	–
**Pulmonary irAE**					
** Pneumonitis G1-5**	Dual ICIs (95.2)	ICI monotherapy (64.1)	Dual ICIs + CT (31.9)	ICI monotherapy + CT (58.6)	Chemotherapy (98.5)
** Pneumonitis G3-5**	Dual ICIs (88.5)	ICI monotherapy (62.6)	Dual ICIs + CT (35.4)	ICI monotherapy + CT (67.9)	Chemotherapy (87.1)
**Endocrine irAE**					
** Hyperthyroidism G1-5**	Dual ICIs (92.7)	ICI monotherapy (58.5)	Dual ICIs + CT (51.3)	ICI monotherapy + CT (89.2)	Chemotherapy (100.0)
** Hyperthyroidism G3-5**	Dual ICIs + CT (57.1)	Dual ICIs (32.2)	ICI monotherapy + CT (29.1)	ICI monotherapy (25.4)	Chemotherapy (31.8)
** Hypothyroidism G1-5**	Dual ICIs (88.0)	ICI monotherapy (76.6)	Dual ICIs + CT (45.3)	ICI monotherapy + CT (60.8)	Chemotherapy (100.0)
** Hypothyroidism G3-5**	Dual ICIs + CT (83.9)	Dual ICIs (38.4)	ICI monotherapy + CT (28.2)	ICI monotherapy (36.3)	Chemotherapy (57.6)
** Thyroiditis G1-5**	Dual ICIs (47.6)	ICI monotherapy (35.7)	ICI monotherapy + CT (32.0)	Dual ICIs + CT (30.3)	Chemotherapy (87.1)
** Thyroiditis G3-5**	Dual ICIs + CT (34.5)	ICI monotherapy + CT (27.7)	Chemotherapy (34.4)	ICI monotherapy (32.3)	Dual ICIs (31.6)
** Hypophysitis G1-5**	Dual ICIs + CT (58.6)	Dual ICIs (44.3)	ICI monotherapy + CT (40.5)	ICI monotherapy (47.6)	Chemotherapy (88.3)
** Hypophysitis G3-5**	Dual ICIs (57.5)	Dual ICIs + CT (24.0)	ICI monotherapy (33.8)	ICI monotherapy + CT (38.7)	Chemotherapy (66.0)
** Diabetes G1-5**	Dual ICIs (62.7)	ICI monotherapy + CT (43.7)	Dual ICIs + CT (24.0)	ICI monotherapy (27.4)	Chemotherapy (39.0)
** Diabetes G3-5**	Dual ICIs (49.3)	ICI monotherapy + CT (39.5)	Dual ICIs + CT (22.4)	ICI monotherapy (27.0)	Chemotherapy (38.3)
** Skin irAE**					
** Pruritus G1-5**	ICI monotherapy + CT (42.4)	Dual ICIs (23.9)	ICI monotherapy (38.8)	Chemotherapy (87.0)	–
** Pruritus G3-5**	ICI monotherapy + CT (57.6)	Dual ICIs (22.1)	Chemotherapy (38.0)	ICI monotherapy (46.2)	–
** Rash G1-5**	Dual ICIs + CT (51.8)	Dual ICIs (49.0)	ICI monotherapy + CT (62.5)	ICI monotherapy (64.6)	Chemotherapy (99.3)
** Rash G3-5**	Dual ICIs + CT (89.6)	ICI monotherapy + CT (49.5)	Dual ICIs (25.1)	ICI monotherapy (36.8)	Chemotherapy (70.0)
** Severe skin reaction G1-5**	Dual ICIs (88.4)	ICI monotherapy (86.2)	ICI monotherapy + CT (87.2)	Chemotherapy (91.1)	–
** Severe skin reaction G3-5**	Dual ICIs (93.4)	ICI monotherapy (87.0)	ICI monotherapy + CT (79.3)	Chemotherapy (87.5)	–
**Other irAE**					
** Myocarditis G1-5**	Dual ICIs (61.1)	ICI monotherapy (44.6)	Dual ICIs + CT (30.2)	Chemotherapy (40.2)	ICI monotherapy + CT (49.7)
** Myocarditis G3-5**	Dual ICIs (60.0)	ICI monotherapy (42.2)	Dual ICIs + CT (28.8)	Chemotherapy (37.7)	ICI monotherapy + CT (37.4)
** Nephritis G1-5**	ICI monotherapy + CT (42.4)	Dual ICIs (23.9)	ICI monotherapy (38.8)	Chemotherapy (87.0)	–
** Nephritis G3-5**	ICI monotherapy + CT (57.6)	Dual ICIs (22.1)	Chemotherapy (38.0)	ICI monotherapy (46.2)	–
** Hepatitis G1-5**	Dual ICIs (75.6)	ICI monotherapy (44.7)	Dual ICIs + CT (38.1)	ICI monotherapy + CT (69.6)	Chemotherapy (98.5)
** Hepatitis G3-5**	Dual ICIs + CT (49.8)	Dual ICIs (25.8)	ICI monotherapy + CT (38.2)	ICI monotherapy (60.0)	Chemotherapy (93.6)
** Myositis G1-5**	Dual ICIs (52.8)	ICI monotherapy (48.7)	ICI monotherapy + CT (34.7)	Chemotherapy (53.4)	–
** Myositis G3-5**	Dual ICIs (55.2)	ICI monotherapy + CT (25.0)	Chemotherapy (35.7)	ICI monotherapy (44.4)	–
** Hypersensitivity/infusion reaction G1-5**	Dual ICIs + CT (77.3)	Dual ICIs (39.6)	ICI monotherapy + CT (25.3)	ICI monotherapy (34.0)	Chemotherapy (45.1)
** Hypersensitivity/infusion reaction G3-5**	ICI monotherapy + CT (64.7)	Chemotherapy (33.8)	Dual ICIs + CT (16.7)	Dual ICIs (27.3)	ICI monotherapy (36.5)

The value in each parenthesis represents the probability of risk to rank (%). ICI, immune checkpoint inhibitor; CT, chemotherapy; irAE, immune-related adverse event; G, grade.

For gastrointestinal irAEs (**Figures 4A, B**), ICI regimens were associated with higher risk of colitis than chemotherapy, with dual ICIs showing the highest risk (incidence: 3.66% for grade 1–5 and 2.53% for grade 3–5; ranking probability: 91.6% for grade 1–5 and 92.3% for grade 3–5). Nevertheless, a different trend was detected in the case of diarrhea. ICI regimens, especially ICI monotherapy, had a lower risk of causing diarrhea than treatment strategies that included chemotherapy.

For pulmonary irAEs, pneumonitis was significantly higher in patients receiving dual ICIs than in those receiving ICI monotherapy (RR: 0.43, 95% CI: 0.25–0.74 for ICI monotherapy *vs.* dual ICIs in grade 1–5; RR: 2.29, 95% CI: 1.23–4.28 in grade 3–5), or ICI monotherapy + chemotherapy (RR: 0.27, 95% CI: 0.12–0.59 for ICI monotherapy + chemotherapy *vs.* dual ICIs in grade 1–5; RR: 5.01, 95% CI: 1.83–13.67 in grade 3–5) (**Figure 4C**). The ranking order of ICIs regimens was as follows: dual ICIs (probability: 95.2% for grade 1–5; 88.5% for grade 3–5), ICI monotherapy (64.1%; 62.6%), dual ICIs + chemotherapy (31.9%; 35.4%), and ICI monotherapy + chemotherapy (58.6%; 67.9%).

For endocrine irAEs, patients administering dual ICIs with or without chemotherapy seemed to be associated with a high risk of hyperthyroidism, hypothyroidism, hypophysitis, thyroiditis, and diabetes. Owing to the low incidence of serious endocrine irAEs, no positive RRs were detected among the individual ICI strategies (**Figures 4D–G**).

For skin irAEs, the use of ICI monotherapy + chemotherapy, dual ICIs + chemotherapy, and dual ICIs presented the highest risk of pruritus, rash, and severe skin reaction, respectively (**Figures 5A–C**). For other irAEs, no significant difference was observed among the ICI regimens (**Figures 5D–H**).

### Sensitivity Analysis in Network Meta-Analysis

Thirty phase III RCTs, and 29 studies that enrolled patients with NSCLC were separately included in the sensitivity analyses. The observed ranking orders were consistent with the original NMA, irrespective of the overall AEs and irAEs (**Table S15**).

## Discussion

### Major Findings and Interpretation

The current NMA highlights the toxicity profile of ICI–based treatments among patients with advanced lung cancer based on 38 RCTs involving 22,178 patients. Our results indicated that the use of mono- or dual-ICI therapy may reduce the risk of treatment–related AEs at the expense of an increased risk of developing irAEs compared with chemotherapy. During therapy with ICI regimens, patients administering dual ICIs with chemotherapy may experience most AEs (either grade 1–5 or grade 3–5) and irAEs of any grade, and those receiving dual ICIs may have the highest risk of developing serious irAEs. There were differences observed in the toxicity spectra among the five treatment therapies. However, these results should be carefully interpreted because of the limited number of studies on groups receiving dual ICIs with and without chemotherapy.

### Comparison With Previous Studies

Recently, developments in lung cancer patients with advanced stages of the disease have shown immunotherapy as a promising treatment option. However, the expanded use of ICIs has resulted in noticeable growth in adverse events, particularly irAEs (6, 68). With more treatment options with ICIs now approved for advanced lung cancer, a robust analysis is urgently required to compare the risk of safety profiles among all of the different treatment regimens.

To date, several systematic reviews and meta-analyses have been conducted to assess the safety profiles of ICIs in patients with cancer, yet few studies have focused on patients with lung cancer. The earliest meta-analysis, which pooled 22 RCTs for evaluating rare but severe irAEs resulting from the use of PD-1 and PD-L1 inhibitors in patients with NSCLC, indicated an increased all–grade pneumonitis risk from ICIs than chemotherapy (OR = 2.35, 95% CI, 1.32–4.20, *P* = 0.004) (15). However, this study had limited value because of the inclusion of minimal trials with a control group, which inevitably led to insufficient evidence for a conclusion; in addition, only ICI monotherapy was investigated. In 2020, an updated NMA of 25 RCTs was conducted for chemotherapy, ICI monotherapy, dual ICIs combination, and ICIs + chemotherapy, and reported a significantly reduced risk of immune–related pneumonitis in patients with lung cancer following ICIs + chemotherapy when compared with dual ICIs combination and ICI monotherapy (14). Furthermore, three other meta-analyses focused on gastrointestinal irAEs, including diarrhea and/or colitis, and consistently found that PD-1/PD-L1 inhibitors might lead to a higher risk of immune–mediated colitis but might result in a reduction in diarrhea when compared with chemotherapy (16–18). Notably, the above–mentioned studies focused on one specific irAE, making it difficult to unveil the panorama of toxicity from ICIs. Recently, Berti et al. compared overall and organ–specific irAEs between immunotherapy and immune–chemotherapy in lung cancer based on 16 phase III clinical trials and found that immunotherapy alone showed a significantly lower risk of irAEs than immunochemotherapy (69). The study investigators excluded phase II RCTs and ignored the discrepancy between ICI monotherapy and dual ICI combination therapy. Given these limitations, the current NMA comprehensively estimated the overall and organ–specific toxicity spectrum among all up to date ICI regimens by pooling all currently available phase II and III clinical trials involving patients with NSCLC and SCLC.

### Safety Profile of ICI Regimens in Patients With Lung Cancer

It is well known that the incidence of overall adverse events during ICI monotherapy is lower than that for conventional chemotherapy, while exposure to immunotherapy increases the risk of irAEs, as seen in the present study. Interestingly, further analyses of ICI–based regimens found that ICI monotherapy + chemotherapy decreased the risk of grade 1–5 and grade 3–5 irAEs compared with ICI monotherapy and dual ICIs therapy. This trend was also observed in pneumonitis and myocarditis (grade1–5 and grade 3–5), as well as grade 1–5 hyperthyroidism, hypothyroidism, thyroiditis, severe skin reaction, hepatitis, myositis based on ranking. Consistent with our study, Chen et al. reported that the use of an ICI with chemotherapy led to less pneumonitis than use of ICI monotherapy or dual ICIs combination (14). One possible reason for the decreased risk in irAEs when chemotherapy is added to ICI regimens may be due to the fact that conventional chemotherapy consists of cytotoxic agents that are believed to cause chemotherapy–induced immunosuppression, augmenting stress on the entire immune system and resulting in a reduced immune function (70, 71). Another contributing factor might be the use of corticosteroids. In chemotherapy regimens containing cytotoxic agents such as platinum, pemetrexed and taxanes, which are the standard treatments for lung cancer, corticosteroids are commonly prescribed as binding pre-treatment for antiemetic and antiallergy purposes. The baseline or early use of corticosteroids at the time of initiating ICI therapy could blunt a proliferative burst of CD8-positive T cells, which are otherwise needed for the ICI therapeutic response, thus affecting the efficacy and toxicity (72, 73). In addition, corticosteroids are recommended immunosuppressive agents for various mild-to-severe irAEs such as pneumonitis, colitis, hepatitis, thyroiditis, rash, etc. (74). Accordingly, the risk of experiencing irAEs may be underestimated in circumstances with corticosteroids.

Some studies have pointed out that the incidence of irAEs in patients on a combination of two ICIs was higher than that observed with ICI monotherapy. In the present study, we did not observe any statistical differences between these two groups in terms of overall grade 1–5 irAEs, let alone for rare irAEs, such as thyroiditis, diabetes, myocarditis, nephritis, hepatitis, and myositis. Even between dual ICIs + chemotherapy and ICI monotherapy (both grade 1–5 and grade 3–5 irAEs), significant difference was not detected. However, this trend can be seen from the ranking either in grade 1–5 irAEs or grade 3–5 irAEs. Given the fact that a limited number of studies directly compared dual ICIs (with or without chemotherapy) with ICIs monotherapy, these results should be interpreted with caution. Therefore, more high–quality RCTs are needed to investigate the incidence of irAEs among different ICI–based regimens.

### Clinical Implication

Our results provide possible safety speculations for clinical decision–making to tailor the best immunotherapy strategy for each patient with lung cancer. For instance, administration of an individual ICI or dual ICIs plus chemotherapy was reported to have a significantly lower risk of pneumonitis (grade 1–5 and grade 3–5) than ICI monotherapy and dual ICIs therapy and could perhaps be preferred in selected cases of lung fibrosis or severe chronic obstructive pulmonary disease. In addition, ICI monotherapy was associated with the lowest risk for both diarrhea and colitis among ICI regimens and could perhaps be preferred in selected patients in whom gastrointestinal irAEs could be a concern. Of course, these results should be proven in prospective registries or cohorts to better understand the safety of novel ICI–based options in this subset of patients.

### Study Strengths and Limitations

The major strength of this study was to depict a full view of the safety profile of ICIs at different levels (risk of overall AEs of any grade and grade 3–5, any irAEs and severe irAEs by individual organs/system). Second, owing to the different toxicity spectrum based on cancer types, the study focused on a specific population of patients (i.e., with lung cancer). Third, except for combination of ICIs and targeted agents, we included all available ICI–based regimens to aid clinicians to tailor the ICI strategy for individual patient with lung cancer.

However, several limitations of this study need to be acknowledged. First, the included RCTs used different terms to describe irAEs. In clinical settings, AEs or irAEs are usually recognised and reported depending on the evaluation of the physician and are diagnosed based on their experience. Therefore, the identification of irAEs might not be completely accurate and might lead to bias in the assessment. In addition, few studies reported irAEs during the entire ICI monotherapy maintenance as separate outcome, making it difficult to investigate irAEs during this period. Second, the included studies showed heterogeneity in terms of subtype of cancer, pharmacological strategy, follow–up time, and other factors. As we focused on outlining the entire safety profile of ICI agents, subgroup analyses based on patients’ histology, specified ICIs and kind of chemotherapy were not performed. However, we performed sensitivity analyses and meta-regression in pairwise meta-analysis, as well as sensitivity analyses in NMA, to control for these possible confounders. Third, inconsistent results between direct and indirect comparisons were observed. Unlike direct comparison, network meta-analysis included both direct evidence and indirect effects from the other studies. Because of above-mentioned heterogeneity among RCTs, integrated results possibly underestimated or overestimated the actual results. Fourth, we did not obtain access to comorbidity data, which might be high–risk factors for certain irAEs. Lastly, we did not have the resources to review non-English publications. However, we enrolled studies identified following a comprehensive search of broad databases and are thus confident that this study covered the majority of trials in these special patients. Given aforementioned limitations, further studies are needed to confirm our findings.

## Conclusions

In summary, this network meta-analysis contributes to clarifying the frequency and characteristics of adverse events during ICI treatment in patients with advanced lung cancer. We found that ICI monotherapy + chemotherapy had the best immune–related safety profile, followed by ICI monotherapy, dual ICIs therapy, dual ICIs + chemotherapy for grade 1–5 irAEs, and ICI monotherapy, dual ICIs + chemotherapy, dual ICIs therapy for grade 3–5 irAEs. The safety ranking of ICI-based choices is modulated by specific irAEs and severity.

## Data Availability Statement

The original contributions presented in the study are included in the article/**Supplementary Material**. Further inquiries can be directed to the corresponding authors.

## Author Contributions

Z-CG, X-YC, and H-WL are the guarantors of the entire manuscript. Z-CG and Y-DY contributed to the study conception and design, critical revision of the manuscript for important intellectual content, and final approval of the version to be published. J-JC, JF, and Y-JS contributed to the data acquisition, analysis, and interpretation. All authors contributed to the article and approved the submitted version.

## Funding

This study was supported by the Ren Ji Boost Project of National Natural Science Foundation of China (RJTJ-JX-001), the Research Funds of Shanghai Health and Family Planning Commission (20184Y0022), Clinical Pharmacy Innovation Research Institute of Shanghai Jiao Tong University School of Medicine (CXYJY2019ZD001), and Shanghai “Rising Stars of Medical Talent” Youth Development Program – Youth Medical Talents – Clinical Pharmacist Program [SHWJRS (2019) 072].

## Conflict of Interest

The authors declare that the research was conducted in the absence of any commercial or financial relationships that could be construed as a potential conflict of interest.

## Publisher’s Note

All claims expressed in this article are solely those of the authors and do not necessarily represent those of their affiliated organizations, or those of the publisher, the editors and the reviewers. Any product that may be evaluated in this article, or claim that may be made by its manufacturer, is not guaranteed or endorsed by the publisher.
