# Supplementary material for: A Network Comparison on Safety Profiling of Immune Checkpoint Inhibitors in Advanced Lung Cancer
Source: Front Immunol. 2021 Dec 3;12:760737. doi: 10.3389/fimmu.2021.760737 (PMC8677695; doi:10.3389/fimmu.2021.760737)
Supplement: Supplementary file 1 [file DataSheet_1.docx]

SUPPLEMENTAL FILE

**Title:** A network comparison on safety profiling of immune checkpoint inhibitors in advanced lung cancer

**Contents**

**Tables**

[Table S1. Checklist of the PRISMA extention for network meta-analysis 3](#_Toc87185041)

[Table S2. Search strategy 8](#_Toc87185042)

[Table S3. Definition of immune-related adverse events 9](#_Toc87185043)

[Table S4. Excluded studies with reasons 11](#_Toc87185044)

[Table S5. Patient demographics and clinical characteristics of RCTs 14](#_Toc87185045)

[Table S6. Quality assessment of RCTs 18](#_Toc87185046)

[Table S7. Direct comparisons of treatments for the risk of adverse events. 19](#_Toc87185047)

[Table S8. Sensitivity analysis in pair-wise meta-analysis 27](#_Toc87185048)

[Table S9. Univariable meta-regression for primary outcomes 29](#_Toc87185049)

[Table S10. Trim and fill method to deal with publication bias 30](#_Toc87185050)

[Table S11. Pooled incidence of adverse events 31](#_Toc87185051)

[Table S12. Ranking of treatments for AE and irAE based on cumulative probability 36](#_Toc87185052)

[Table S13. Evaluation of inconsistency for the primary outcomes among different treatment regimen 37](#_Toc87185053)

[Table S14. Node–splitting analysis of network meta-analysis 39](#_Toc87185054)

[Table S15. Sensitivity analysis in network meta-analysis 40](#_Toc87185055)

**Figure**

[Figure S1. Publication bias for treatments 42](#_Toc87185056)

[References 42](#_Toc87185057)

# Table S1. Checklist of the PRISMA extention for network meta-analysis

| **Section/Topic** | **Item #** | **Checklist Item** | **Reported on Page #** |
| --- | --- | --- | --- |
| **TITLE** |  |  |  |
| Title | 1 | Identify the report as a systematic review *incorporating a network meta-analysis (or related form of meta-analysis).* | **1** |
|  |  |  |  |
| **ABSTRACT** |  |  | 2-3 |
| Structured summary | 2 | Provide a structured summary including, as applicable:  **Background:** main objectives  **Methods:** data sources; study eligibility criteria, participants, and interventions; study appraisal; and *synthesis methods, such as network meta-analysis.*  **Results:** number of studies and participants identified; summary estimates with corresponding confidence/credible intervals; *treatment rankings may also be discussed. Authors may choose to summarize pairwise comparisons against a chosen treatment included in their analyses for brevity.*  **Discussion/Conclusions:** limitations; conclusions and implications of findings.  **Other:** primary source of funding; systematic review registration number with registry name. |  |
|  |  |  |  |
| **INTRODUCTION** |  |  |  |
| Rationale | 3 | Describe the rationale for the review in the context of what is already known*, including mention of why a network meta-analysis has been conducted.* | **4-5** |
| Objectives | 4 | Provide an explicit statement of questions being addressed, with reference to participants, interventions, comparisons, outcomes, and study design (PICOS). | **4-5** |
|  |  |  |  |
| **METHODS** |  |  |  |
| Protocol and registration | 5 | Indicate whether a review protocol exists and if and where it can be accessed (e.g., Web address); and, if available, provide registration information, including registration number. | **5** |
| Eligibility criteria | 6 | Specify study characteristics (e.g., PICOS, length of follow-up) and report characteristics (e.g., years considered, language, publication status) used as criteria for eligibility, giving rationale. *Clearly describe eligible treatments included in the treatment network, and note whether any have been clustered or merged into the same node (with justification).* | **5-6** |
| Information sources | 7 | Describe all information sources (e.g., databases with dates of coverage, contact with study authors to identify additional studies) in the search and date last searched. | **5** |
| Search | 8 | Present full electronic search strategy for at least one database, including any limits used, such that it could be repeated. | Appendix  page 8 |
| Study selection | 9 | State the process for selecting studies (i.e., screening, eligibility, included in systematic review, and, if applicable, included in the meta-analysis). | **5-6** |
| Data collection process | 10 | Describe method of data extraction from reports (e.g., piloted forms, independently, in duplicate) and any processes for obtaining and confirming data from investigators. | **6** |
| Data items | 11 | List and define all variables for which data were sought (e.g., PICOS, funding sources) and any assumptions and simplifications made. | **6** |
| **Geometry of the network** | **S1** | Describe methods used to explore the geometry of the treatment network under study and potential biases related to it. This should include how the evidence base has been graphically summarized for presentation, and what characteristics were compiled and used to describe the evidence base to readers. | **7** |
| Risk of bias within individual studies | 12 | Describe methods used for assessing risk of bias of individual studies (including specification of whether this was done at the study or outcome level), and how this information is to be used in any data synthesis. | **6-7** |
| Summary measures | 13 | State the principal summary measures (e.g., risk ratio, difference in means). *Also describe the use of additional summary measures assessed, such as treatment rankings and surface under the cumulative ranking curve (SUCRA) values, as well as modified approaches used to present summary findings from meta-analyses.* | **7-8** |
| Planned methods of analysis | 14 | Describe the methods of handling data and combining results of studies for each network meta-analysis. This should include, but not be limited to:   - *Handling of multi-arm trials;* - *Selection of variance structure;* - *Selection of prior distributions in Bayesian analyses; and* - *Assessment of model fit.* | **7-8** |
| **Assessment of Inconsistency** | **S2** | Describe the statistical methods used to evaluate the agreement of direct and indirect evidence in the treatment network(s) studied. Describe efforts taken to address its presence when found. | **7** |
| Risk of bias across studies | 15 | Specify any assessment of risk of bias that may affect the cumulative evidence (e.g., publication bias, selective reporting within studies). | **6-7** |
| Additional analyses | 16 | Describe methods of additional analyses if done, indicating which were pre-specified. This may include, but not be limited to, the following:   - Sensitivity or subgroup analyses; - Meta-regression analyses; - *Alternative formulations of the treatment network; and* - *Use of alternative prior distributions for Bayesian analyses (if applicable).* | **7** |
|  |  |  |  |
| **RESULTS†** |  |  |  |
| Study selection | 17 | Give numbers of studies screened, assessed for eligibility, and included in the review, with reasons for exclusions at each stage, ideally with a flow diagram. | **8** |
| **Presentation of network structure** | **S3** | Provide a network graph of the included studies to enable visualization of the geometry of the treatment network. | **8** |
| **Summary of network geometry** | **S4** | Provide a brief overview of characteristics of the treatment network. This may include commentary on the abundance of trials and randomized patients for the different interventions and pairwise comparisons in the network, gaps of evidence in the treatment network, and potential biases reflected by the network structure. | **8** |
| Study characteristics | 18 | For each study, present characteristics for which data were extracted (e.g., study size, PICOS, follow-up period) and provide the citations. | **8** |
| Risk of bias within studies | 19 | Present data on risk of bias of each study and, if available, any outcome level assessment. | **8-9** |
| Results of individual studies | 20 | For all outcomes considered (benefits or harms), present, for each study: 1) simple summary data for each intervention group, and 2) effect estimates and confidence intervals. *Modified approaches may be needed to deal with information from larger networks.* | **8-13** |
| Synthesis of results | 21 | Present results of each meta-analysis done, including confidence/credible intervals. *In larger networks, authors may focus on comparisons versus a particular comparator (e.g. placebo or standard care), with full findings presented in an appendix. League tables and forest plots may be considered to summarize pairwise comparisons.* If additional summary measures were explored (such as treatment rankings), these should also be presented. | **8-13** |
| **Exploration for inconsistency** | **S5** | Describe results from investigations of inconsistency. This may include such information as measures of model fit to compare consistency and inconsistency models, *P* values from statistical tests, or summary of inconsistency estimates from different parts of the treatment network. | **12** |
| Risk of bias across studies | 22 | Present results of any assessment of risk of bias across studies for the evidence base being studied. | **8-9** |
| Results of additional analyses | 23 | Give results of additional analyses, if done (e.g., sensitivity or subgroup analyses, meta-regression analyses*, alternative network geometries studied, alternative choice of prior distributions for Bayesian analyses,* and so forth). | **13** |
|  |  |  |  |
| **DISCUSSION** |  |  |  |
| Summary of evidence | 24 | Summarize the main findings, including the strength of evidence for each main outcome; consider their relevance to key groups (e.g., healthcare providers, users, and policy-makers). | **13-17** |
| Limitations | 25 | Discuss limitations at study and outcome level (e.g., risk of bias), and at review level (e.g., incomplete retrieval of identified research, reporting bias). *Comment on the validity of the assumptions, such as transitivity and consistency. Comment on any concerns regarding network geometry (e.g., avoidance of certain comparisons).* | **17-18** |
| Conclusions | 26 | Provide a general interpretation of the results in the context of other evidence, and implications for future research. | **18** |
|  |  |  |  |
| **FUNDING** |  |  |  |
| Funding | 27 | Describe sources of funding for the systematic review and other support (e.g., supply of data); role of funders for the systematic review. This should also include information regarding whether funding has been received from manufacturers of treatments in the network and/or whether some of the authors are content experts with professional conflicts of interest that could affect use of treatments in the network. | **18-19** |

PICOS = population, intervention, comparators, outcomes, study design. * Text in italics indicates wording specific to reporting of network meta-analyses that has been added to guidance from the PRISMA statement. † Authors may wish to plan for use of appendices to present all relevant information in full detail for items in this section.

# Table S2. Search strategy

| **Search strategy for PubMed, EMBASE, and Cochrane Library databases** | | | |
| --- | --- | --- | --- |
| Patient | #1 | (((lung cancer[Title/Abstract]) OR (lung carcinoma[Title/Abstract])) OR (NSCLC[Title/Abstract])) OR (SCLC[Title/Abstract]) | |
| Intervention | #2 | ((((((((((((((((((((((Immune Checkpoint Inhibitor[Title/Abstract]) OR (ICI[Title/Abstract])) OR (immune therapy[Title/Abstract])) OR (immunotherapy[Title/Abstract])) OR (cytotoxic T-lymphocyte associated antigen-4[Title/Abstract])) OR (CTLA-4[Title/Abstract])) OR (Ipilimumab[Title/Abstract])) OR (Tremelimumab[Title/Abstract])) OR (programmed cell death protein-1[Title/Abstract])) OR (programmed cell death protein[Title/Abstract])) OR (PD-1[Title/Abstract])) OR (nivolumab[Title/Abstract])) OR (pembrolizumab[Title/Abstract])) OR (camrelizumab[Title/Abstract])) OR (sintilimab[Title/Abstract])) OR (toripalimab[Title/Abstract])) OR (tislelizumab[Title/Abstract])) OR (programmed cell death-Ligand 1[Title/Abstract])) OR (PD-L1[Title/Abstract])) OR (atezolizumab[Title/Abstract])) OR (durvalumab[Title/Abstract])) OR (avelumab[Title/Abstract])) OR (cemiplimab[Title/Abstract]) | |
| Study type | #3 | (((((Randomized Controlled Tial[Title/Abstract]) OR (Controlled Cinical Trial[Title/Abstract])) OR (Randomized[Title/Abstract])) OR (Placebo[Title/Abstract])) OR (Randomly[Title/Abstract])) OR (Trial[Title/Abstract]) | |
| Language | #4 | English [language] | |
| Search strategy | #1 AND #2 AND #3 AND #4 Filters: from 2010/1/1 - 2021/4/19 | | |
| **Search terms in ClinicalTrials.gov** | | | |
| 1 | Patient | | Lung cancer |
| 2 | Intervention | | “nivolumab” OR “pembrolizumab” OR “camrelizumab” OR “sintilimab” OR “toripalimab” OR “tislelizumab” OR “atezolizumab” OR “durvalumab” OR “avelumab” OR “cemiplimab” |
| 3 | Study phase | | Phase II and phase III clinical trial |
| 4 | Study status | | Completed and with results |

# Table S3. Definition of immune-related adverse events

| **Study, year** | **Definition of irAE** |
| --- | --- |
| CA184-041, 2012 | IrAE was defined as an AE that was treatment-related and consistent with an immune-mediated event. |
| CA184-041, 2013 | IrAE was defined as an AE that was treatment-related and consistent with an immune-mediated event. |
| CheckMate 057, 2015 | Select adverse events (those with a potential immunologic cause) were grouped according to prespecified categories. |
| CheckMate 017, 2015 | Select adverse events (those with potential immunologic causes) were grouped according to prespecified categories. |
| CheckMate 032, 2016 | Treatment-related adverse events in immunotherapy. |
| POPLAR , 2016 | Immune-mediated adverse events. |
| KEYNOTE-010, 2016 | Adverse events of special interest based on their likely immune aetiology, irrespective of attribution to study treatment. |
| KEYNOTE-021, 2016 | Adverse events of interest based on a presumed immunological mechanism of action. |
| CA184-156, 2016 | AEs of interest were defined as those with potential immunologic etiologies and grouped into predefined categories: enterocolitis, dermatitis, hepatitis, endocrinopathies, and neuropathies. AEs ofinterest considered drug related by the investigator were referred to as immune-related AEs. |
| KEYNOTE 024, 2016 | The immune-mediated events, both those that were and those that were not attributed to study treatment by the investigator, are listed. |
| CheckMate 026, 2017 | Select adverse events are those with potential immunologic etiology that require frequent monitoring/intervention. |
| Study 104, 2017 | AEs of interest consistent with an immune-mediated mechanism (including enterocolitis, dermatitis, hepatitis, endocrinopathies, and neuropathies) and considered drug related by the investigator were classified as immunerelated AEs (irAEs). |
| OAK, 2017 | Immunemediated adverse events. |
| JAVELIN Lung 200, 2018 | Immunemediated adverse events. |
| KEYNOTE-189, 2018 | Immune-mediated adverse events were defined on the basis of a list of terms specified by the sponsor and were included in the analysis regardless of whether they were attributed to treatment by the investigator. |
| IMpower133, 2018 | Immune-related AEs were defined using MedDRA Preferred Terms that included both diagnosed immune conditions and signs and symptoms potentially representative of immune-related events, regardless of investigator-assessed causality. |
| KEYNOTE-407, 2018 | The adverse events of interest are infusion reactions and events with an immune-related cause. |
| CheckMate 227, 2019 | Select AEs are those with potential immunologic etiology that require frequent monitoring/intervention. |
| KEYNOTE-042, 2019 | The events of interest are infusion reactions and events with an immune-mediated cause regardless of attribution to treatment by investigators. |
| IFCT-1603, 2019 | Specific immunological adverse events. |
| IMpower130, 2019 | Immune-related adverse events. |
| CheckMate 078, 2019 | Treatment-related select adverse events. |
| PROLUNG, 2020 | Immune-related adverse events. |
| IMpower110, 2020 | Immune-mediated adverse events were defined according to a list of sponsor-specified terms, regardless of whether these events led to use of systemic glucocorticoids, endocrine therapy, or other immunosuppressants. |
| IMpower131, 2020 | Immune-related adverse events. |
| IMpower132, 2020 | Adverse events of special interest. |
| ARCTIC, 2020 | AEs of special interest (AESIs) included, but were not limited to, events with a potential inflammatory or immune-mediated mechanism that may require more frequent monitoring and/or interventions such as steroids, immunosuppressants, and/or hormone replacement therapy. |
| MYSTIC, 2020 | An adverse event of special interest requiring the use of systemic steroids or other immunosuppressants and/or, for specific endocrine events, endocrine therapy, consistent with an immune-mediated mechanism of action, and where there is no clear alternate etiology. |
| KEYNOTE-604, 2020 | Immue-mediated adverse events. |
| ORIENT-11, 2020 | Immune-related adverse events. |
| CameL, 2020 | Immune-related adverse events. |
| CASPIAN, 2021 | An immunemediated adverse event is defined as an event that is associated with drug exposure and consistent with an immune-mediated mechanism of action, where there is no clear alternate aetiology and the event required treatment with systemic corticosteroids or other immunosuppressants and/or, for specific endocrine events, endocrine therapy. |
| CheckMate 451, 2021 | Treatment-related select adverse events are those with potential immunologic etiology that require frequent monitoring or intervention. |
| CheckMate 9LA, 2021 | Select treatment-related adverse events are events with potential immunologic aetiology that require frequent monitoring/ intervention. |
| CheckMate 331, 2021 | Select treatment-related adverse events are those with potential immunologic etiology that require frequent monitoring/ intervention. |
| KEYNOTE-598, 2021 | Immune-mediated AEs and infusion reactions were defined on the basis of a list of terms specified by the sponsor and considered regardless of attribution to treatment by the investigator. |
| EMPOWER-Lung 1, 2021 | Immune-related adverse events based on a sponsordefined list of terms. |
| RATIONALE 307, 2021 | Potential immune-mediated AEs were selected from a group of preferred terms regardless of whether the investigator attributed the event to a treatment or consideredthe event to be immune related. |

AE: adverse event; irAE: immune-related adverse event.

# Table S4. Excluded studies with reasons

| **Author, year/ NCT number** | **Study** | **Drugs** | **Reason for exclusion** |
| --- | --- | --- | --- |
| Antonia, 2017(Antonia et al., 2017) | PACIFIC | Durvalumab | Placebo as control |
| Brahmer, 2017(Brahmer et al., 2017) | KEYNOTE-024 | Pembrolizumab | Overlap with Reck, 2016(Reck et al., 2016b) |
| Horn, 2017(Horn et al., 2017) | CheckMate 017 and CheckMate 057 | Nivolumab | Overlap with Borghaei, 2015(Borghaei et al., 2015) and Brahmer, 2015(Brahmer et al., 2015) |
| Afzal, 2018(Afzal et al., 2018) | / | Pembrolizumab + CT | Retrospective study |
| Antonia, 2018(Antonia et al., 2018) | PACIFIC | Durvalumab | Placebo as control |
| Bordoni, 2018(Bordoni et al., 2018) | OAK | Atezolizumab | Overlap with Rittmeyer, 2017(Rittmeyer et al., 2017) |
| Fehrenbacher, 2018(Fehrenbacher et al., 2018) | OAK | Atezolizumab | Overlap with Rittmeyer, 2017(Rittmeyer et al., 2017) |
| Gadgeel, 2018(Gadgeel et al., 2018) | KEYNOTE-021 | Pembrolizumab + CT | Overlap with Langer, 2016(Langer et al., 2016) |
| Hellmann, 2018(Hellmann et al., 2018a) | / | Nivolumab + Ipilimumab | Not reported detailed safety data |
| Hellmann, 2018(Hellmann et al., 2018b) | CheckMate 227 | Nivolumab + Ipilimumab | Overlap with Hellmann, 2019(Hellmann et al., 2019) |
| Hida, 2018(Hida et al., 2018) | OAK | Atezolizumab | Overlap with Rittmeyer, 2017(Rittmeyer et al., 2017) |
| Reck, 2018(Reck et al., 2018a) | CheckMate 057 | Nivolumab | Overlap with Borghaei, 2015(Borghaei et al., 2015) |
| Reck, 2018(Reck et al., 2018b) | CheckMate 017 | Nivolumab | Overlap with Brahmer, 2015(Brahmer et al., 2015) |
| Vokes, 2018(Vokes et al., 2018) | CheckMate 017 and CheckMate 057 | Nivolumab | Overlap with Borghaei, 2015(Borghaei et al., 2015) and Brahmer, 2015(Brahmer et al., 2015) |
| Borghaei, 2019(Borghaei et al., 2019) | KEYNOTE-021 | Pembrolizumab + CT | Overlap with Langer, 2016(Langer et al., 2016) |
| Gadgeel, 2019(Gadgeel et al., 2019) | OAK | Atezolizumab | Overlap with Rittmeyer, 2017(Rittmeyer et al., 2017) |
| Levy, 2019(Levy et al., 2019) | / | Pembrolizumab + CC486 | Intervention was drugs other than ICI or chemotherapy |
| Nishio, 2019(Nishio et al., 2019) | IMpower133 | Atezolizumab + CT | Overlap with Horn, 2018(Horn et al., 2018) |
| Owonikoko, 2019(Owonikoko et al., 2019) | CheckMate 451 | Nivolumab + Ipilimumab | Conference abstract |
| Paz-Ares, 2019(Paz-Ares et al., 2019) | CASPIAN | Durvalumab + CT | Overlap with Goldman, 2021(Goldman et al., 2021) |
| Reck, 2019(Reck et al., 2019a) | KEYNOTE-024 | Pembrolizumab | Overlap with Reck, 2016(Reck et al., 2016b) |
| Reck, 2019(Reck et al., 2019b) | CheckMate 227 | Nivolumab + Ipilimumab | Overlap with Hellmann, 2019(Hellmann et al., 2019) |
| von Pawel, 2019(von Pawel et al., 2019) | OAK | Atezolizumab | Overlap with Rittmeyer, 2017(Rittmeyer et al., 2017) |
| Borghaei, 2020(Borghaei et al., 2020) | SWOG S1400A | Durvalumab | Single arm |
| Chalabi, 2020(Chalabi et al., 2020) | POPLAR and OAK | Atezolizumab | Overlap with Fehrenbacher, 2016(Fehrenbacher et al., 2016) and Rittmeyer, 2017(Rittmeyer et al., 2017) |
| Faivre-Finn, 2020(Faivre-Finn et al., 2020) | PACIFIC | Durvalumab | Placebo as control |
| Gadgeel, 2020(Gadgeel et al., 2020) | KEYNOTE-189 | Pembrolizumab + CT | Overlap with Gandhi, 2018(Gandhi et al., 2018) |
| Garassino, 2020(Garassino et al., 2020) | KEYNOTE-189 | Pembrolizumab + CT | Overlap with Gandhi, 2018(Gandhi et al., 2018) |
| Garon, 2020(Garon et al., 2020) | KEYNOTE-189, PARAMOUNT, PRONOUNCE, and JVBL | Pembrolizumab + CT | Review |
| Goldman, 2020(Goldman et al., 2020) | CASPIAN | Durvalumab + CT | Overlap with Goldman, 2021(Goldman et al., 2021) |
| Halmos, 2020(Halmos et al., 2020) | KEYNOTE-021, KEYNOTE-189 and KEYNOTE-407 | Pembrolizumab + CT;  Nivolumab + Ipilimumab | Review |
| Herbst, 2020(Herbst et al., 2020a) | KEYNOTE-010 | Pembrolizumab | Overlap with Herbst, 2016(Herbst et al., 2016) |
| Kahl, 2020(Kahl, 2020) | / | Nivolumab + Ipilimumab | Conference abstract |
| Keeping, 2020(Keeping et al., 2020) | / | Nivolumab | Retrospective study |
| Lu, 2020(Lu et al., 2020) | CheckMate 078 | Nivolumab | Overlap with Wu, 2019(Wu et al., 2019) |
| Mansfield, 2020(Mansfield et al., 2020) | IMpower133 | Atezolizumab + CT | Overlap with Horn, 2018(Horn et al., 2018) |
| Mazieres, 2020(Mazieres et al., 2020) | KEYNOTE-407 | Pembrolizumab + CT | Overlap with Paz-Ares, 2018(Paz-Ares et al., 2018) |
| Paz-Ares, 2020(Paz-Ares et al., 2020a) | PACIFIC | Durvalumab | Placebo as control |
| Paz-Ares, 2020(Paz-Ares et al., 2020b) | KEYNOTE-407 | Pembrolizumab + CT | Overlap with Paz-Ares, 2018(Paz-Ares et al., 2018) |
| Ready, 2020(Ready et al., 2020) | CheckMate 032 | Nivolumab + Ipilimumab | Overlap with Antonia, 2016(Antonia et al., 2016) |
| Satouchi, 2020(Satouchi, 2020) | KEYNOTE-024 | Pembrolizumab | Overlap with Reck, 2016(Reck et al., 2016b) |
| Spigel, 2020(Spigel et al., 2020) | CYPRESS | Checkpoint inhibitors + Pegilodecakin | Intervention was drugs other than ICI or chemotherapy |
| Wu, 2020(Wu et al., 2020) | KEYNOTE-042 | Pembrolizumab | Overlap with Mok, 2019(Mok et al., 2019) |
| Awad, 2021(Awad et al., 2021) | KEYNOTE-021 | Pembrolizumab + CT | Overlap with Langer, 2016(Langer et al., 2016) |
| Mazieres, 2021(Mazieres et al., 2021) | POPLAR and OAK | Atezolizumab | Overlap with Fehrenbacher, 2016(Fehrenbacher et al., 2016) and Rittmeyer, 2017(Rittmeyer et al., 2017) |
| Socinski, 2018(Socinski et al., 2018) | IMpower150 | Atezolizumab + Bevacizumab + CT | Two groups (ICI + targeted + chemotherapy and ICI + targeted therapy) were not involved in the network map |
| NCT03215706 | CheckMate 9LA | Nivolumab + Ipilimumab + CT | Overlap with Paz-Ares, 2021(Paz-Ares et al., 2021) |
| NCT02538666 | CheckMate 451 | Nivolumab + Ipilimumab | Overlap with Owonikoko, 2021(Owonikoko et al., 2021) |
| NCT01454102 | CheckMate 012 | Ipilimumab | Phase I randomised controlled trials |
| NCT01450761 | CA184-156 | Ipilimumab + CT | Overlap with Reck, 2016(Reck et al., 2016a) |
| NCT01285609 | CA184-104 | Ipilimumab + CT | Overlap with Govindan, 2017(Govindan et al., 2017) |
| NCT00527735 | CA184-041 | Ipilimumab + CT | Overlap with Lynch, 2012(Lynch et al., 2012) |
| NCT02613507 | CheckMate 078 | Nivolumab | Overlap with Wu, 2019(Wu et al., 2019) |
| NCT02481830 | CheckMate 331 | Nivolumab | Overlap with Spigel, 2021(Spigel et al., 2021) |
| NCT02041533 | CheckMate 026 | Nivolumab | Overlap with Carbone, 2017(Carbone et al., 2017) |
| NCT01673867 | CheckMate 057 | Nivolumab | Overlap with Borghaei, 2015(Borghaei et al., 2015) |
| NCT01642004 | CheckMate 017 | Nivolumab | Overlap with Brahmer, 2015(Brahmer et al., 2015) |
| NCT03607539 | ORIENT-11 | Sintilimab + CT | Overlap with Yang, 2020(Yang et al., 2020) |
| NCT02395172 | JAVELIN Lung 200 | Avelumab | Overlap with Barlesi, 2018(Barlesi et al., 2018) |
| NCT02763579 | IMpower133 | Atezolizumab + CT | Overlap with Horn, 2018(Horn et al., 2018) |
| NCT02657434 | IMpower132 | Atezolizumab + CT | Overlap with Nishio, 2020(Nishio et al., 2020) |
| NCT02409342 | IMpower110 | Atezolizumab | Overlap with Herbst, 2020(Herbst et al., 2020b) |
| NCT02367794 | IMpower131 | Atezolizumab + CT | Overlap with Jotte, 2020(Jotte et al., 2020) |
| NCT02367781 | IMpower130 | Atezolizumab + CT | Overlap with West, 2019(West et al., 2019) |
| NCT02008227 | OAK | Atezolizumab | Overlap with Rittmeyer, 2017(Rittmeyer et al., 2017) |
| NCT01903993 | POPLAR | Atezolizumab | Overlap with Fehrenbacher, 2016(Fehrenbacher et al., 2016) |

CT: chemotherapy.

# Table S5. Patient demographics and clinical characteristics of RCTs

| **Study, year** | **Total number** | **Median age** | **Male**  **(%)** | **PS 0-1**  **(%)** | **Brain/CNS**  **metastasis (%)** | **Liver**  **metastasis**  **(%)** | **Bone metastasis**  **(%)** | **Current/former**  **smoker (%)** | **Prior**  **surgery**  **(%)** | **Prior**  **radiotherapy**  **(%)** |
| --- | --- | --- | --- | --- | --- | --- | --- | --- | --- | --- |
| CA184-041, 2012(Lynch et al., 2012) | 204 | 60 | 74 | 100 | NR | NR | NR | NR | NR | NR |
| CA184-041, 2013(Reck et al., 2013) | 130 | 58 | 75 | 100 | NR | NR | NR | 89 | NR | NR |
| CheckMate 057,2015(Borghaei et al., 2015) | 582 | 62 | 55 | 100 | 11.7 | NR | NR | 79 | 72 | 48 |
| CheckMate 017, 2015(Brahmer et al., 2015) | 272 | 63 | 76 | 100 | 6.3 | NR | NR | 92 | 53 | 53 |
| CheckMate 032, 2016(Antonia et al., 2016) | 213 | 63 | 60 | NR | NR | NR | NR | 94 | NR | NR |
| POPLAR, 2016(Fehrenbacher et al., 2016) | 287 | 62 | 59 | 100 | NR | NR | NR | 81 | NR | NR |
| KEYNOTE-010, 2016(Herbst et al., 2016) | 1033 | 63 | 62 | 99 | 14.7 | NR | NR | 80 | NR | NR |
| KEYNOTE-021, 2016(Langer et al., 2016) | 123 | 62.5 | 39 | 99 | 12.2 | NR | NR | 81 | NR | NR |
| CA184-156, 2016(Reck et al., 2016a) | 954 | 62 | 67 | 100 | 10.5 | NR | NR | 56 | 14 | 2 |
| KEYNOTE 024, 2016(Reck et al., 2016b) | 305 | 64.5 | 61 | 100 | 9.2 | NR | NR | 92 | NR | NR |
| CheckMate 026, 2017(Carbone et al., 2017) | 541 | 64 | 61 | 99 | 12.8 | 16.6 | NR | 88 | NR | 39 |
| Study 104, 2017(Govindan et al., 2017) | 749 | 64 | 84 | 100 | NR | NR | NR | 87 | NR | NR |
| OAK, 2017(Rittmeyer et al., 2017) | 850 | 64 | 61 | 100 | NR | NR | NR | 82 | NR | NR |
| JAVELIN Lung 200, 2018(Barlesi et al., 2018) | 792 | 64 | 69 | 100 | 10.0 | NR | NR | 83 | NR | NR |
| KEYNOTE-189, 2018(Gandhi et al., 2018) | 616 | 65 | 59 | 99 | 17.5 | NR | NR | 88 | NR | 8 |
| IMpower133, 2018(Horn et al., 2018) | 403 | 64 | 65 | 100 | 8.7 | NR | NR | 97 | 14 | 13 |
| KEYNOTE-407, 2018(Paz-Ares et al., 2018) | 559 | 65 | 81 | 100 | 7.9 | NR | NR | 93 | NR | 7 |
| CheckMate 227, 2019(Hellmann et al., 2019) | 1739 | 64 | 68 | 99 | NR | NR | NR | 94 | NR | NR |
| KEYNOTE-042, 2019(Mok et al., 2019) | 1274 | 63 | 71 | 100 | 5.5 | NR | NR | 78 | NR | 12 |
| IFCT-1603, 2019(Pujol et al., 2019) | 73 | 64.7 | 59 | 85 | NR | NR | NR | 96 | NR | NR |
| IMpower130, 2019(West et al., 2019) | 723 | 64 | 57 | 100 | NR | 14.8 | 27.9 | 89 | NR | NR |
| CheckMate 078, 2019(Wu et al., 2019) | 504 | 60 | 79 | 100 | 14.3 | NR | NR | 70 | NR | NR |
| PROLUNG, 2020(Arrieta et al., 2020) | 78 | 50.1 | 41 | 97 | NR | NR | NR | 56 | NR | NR |
| IMpower110, 2020(Herbst et al., 2020b) | 554 | 64 | 70 | 100 | NR | NR | NR | 87 | NR | NR |
| IMpower131, 2020(Jotte et al., 2020) | 1021 | 66 | 82 | 100 | NR | 20.1 | NR | 92 | NR | NR |
| IMpower132, 2020(Nishio et al., 2020) | 578 | 63.5 | 66 | 100 | NR | 12.6 | NR | 88 | NR | NR |
| ARCTIC, 2020(Planchard et al., 2020) | 351 | 62.5 | 65 | 100 | 10.8 | 19.7 | NR | 78 | NR | NR |
| MYSTIC, 2020(Rizvi et al., 2020) | 1118 | 65 | 69 | 100 | NR | NR | NR | 85 | NR | NR |
| KEYNOTE-604, 2020(Rudin et al., 2020) | 453 | 64 | 65 | 100 | 12.1 | 41.3 | NR | 96 | NR | NR |
| ORIENT-11, 2020(Yang et al., 2020) | 397 | 61 | 76 | 100 | 14.6 | NR | NR | 65 | NR | NR |
| CameL, 2020(Zhou et al., 2020) | 412 | 59 | 72 | 100 | 3.6 | NR | NR | 63 | NR | NR |
| CASPIAN, 2021(Goldman et al., 2021) | 805 | 63 | 71 | 100 | 11.6 | 40.9 | NR | 93 | NR | NR |
| CheckMate 451, 2021(Owonikoko et al., 2021) | 559 | 64 | 64 | 100 | 13.9 | 39.0 | NR | 92 | NR | NR |
| CheckMate 9LA, 2021(Paz-Ares et al., 2021) | 719 | 65 | 70 | 99 | 17.0 | 21.4 | 28.8 | 87 | NR | NR |
| CheckMate 331, 2021(Spigel et al., 2021) | 569 | 62 | 62 | 100 | 16.9 | 36.0 | NR | 91 | NR | NR |
| KEYNOTE-598, 2021(Boyer et al., 2021) | 568 | 64 | 69 | 100 | 10.6 | NR | NR | 91 | NR | 21 |
| EMPOWER-Lung 1, 2021(Sezer et al., 2021) | 710 | 63 | 86 | 100 | 11.7 | NR | NR | 100 | NR | NR |
| RATIONALE 307, 2021(Wang et al., 2021) | 360 | 62 | 92 | 100 | 1.7 | 12.2 | 16.9 | 84 | NR | NR |

PS: performance status; CNS: central nervous system; NR: not report.

# Table S6. Quality assessment of RCTs

| **Study** | **Random sequence generation** | **Allocation concealment** | **Blinding of participants and personnel** | **Blinding of outcome assessment** | **Incomplete outcome data** | **Selective reporting** |
| --- | --- | --- | --- | --- | --- | --- |
| CA184-041, 2012 | Low | Unclear | Low | Unclear | Low | Low |
| CA184-041, 2013 | Low | Unclear | Low | Unclear | Low | Low |
| CheckMate 057, 2015 | Low | Low | High | Low | Low | Low |
| CheckMate 017, 2015 | Low | Low | High | Low | Low | Low |
| CheckMate 032, 2016 | Low | Low | High | Unclear | Low | Low |
| POPLAR , 2016 | Low | Low | High | Low | Low | Low |
| KEYNOTE-010, 2016 | Low | Low | High | Low | Low | Low |
| KEYNOTE-021, 2016 | Low | Low | High | Low | Low | Low |
| CA184-156, 2016 | Low | Low | Low | Low | Low | Low |
| KEYNOTE 024, 2016 | Low | Low | High | Unclear | Low | Low |
| CheckMate 026, 2017 | Low | Low | High | Unclear | Low | Low |
| Study 104, 2017 | Low | Unclear | High | Unclear | Low | Low |
| OAK, 2017 | Low | High | High | Low | Low | Low |
| JAVELIN Lung 200, 2018 | Low | High | High | Low | Low | Low |
| KEYNOTE-189, 2018 | Low | Low | Low | Low | Low | Low |
| IMpower133, 2018 | Low | Low | Low | Low | Low | Low |
| KEYNOTE-407, 2018 | Low | Low | Low | Low | Low | Low |
| CheckMate 227, 2019 | Low | Low | High | Low | Low | Low |
| KEYNOTE-042, 2019 | Low | Low | High | Low | Low | Low |
| IFCT-1603, 2019 | Low | Unclear | Unclear | Unclear | Low | Low |
| IMpower130, 2019 | Low | Low | High | Low | Low | Low |
| CheckMate 078, 2019 | Low | High | High | Unclear | Low | Low |
| PROLUNG, 2020 | Low | Unclear | High | Low | Low | Low |
| IMpower110, 2020 | Low | Low | High | Low | Low | Low |
| IMpower131, 2020 | Low | Low | High | Low | Low | Low |
| IMpower132, 2020 | Low | Unclear | High | Unclear | Low | Low |
| ARCTIC, 2020 | Low | Unclear | High | Unclear | Low | Low |
| MYSTIC, 2020 | Low | Unclear | High | Unclear | Low | Low |
| KEYNOTE-604, 2020 | Low | Unclear | Low | Low | Low | Low |
| ORIENT-11, 2020 | Low | Low | Low | Unclear | Low | Low |
| CameL, 2020 | Low | Low | High | Low | Low | Low |
| CASPIAN, 2021 | Low | High | High | Low | Low | Low |
| CheckMate 451, 2021 | Low | Unclear | Low | Low | Low | Low |
| CheckMate 9LA, 2021 | Low | Low | High | Low | Low | Low |
| CheckMate 331, 2021 | Low | Unclear | High | Low | Low | Low |
| KEYNOTE-598, 2021 | Low | Low | Low | Low | Low | Low |
| EMPOWER-Lung 1, 2021 | Low | High | High | Low | Low | Low |
| RATIONALE 307, 2021 | Low | Unclear | High | Low | Low | Low |

# **Table S7. Direct comparisons of treatments for the risk of adverse events.**

| **Treatment comparison** | **Treatment 1 case/No.** | **Treatment 2 case/No.** | **No. of studies** | **RR** | **95%CI** | ***I^2^*** |
| --- | --- | --- | --- | --- | --- | --- |
| **Grade 1-5 adverse events** |  |  |  |  |  |  |
| ICI monotherapy vs. Chemotherapy | 3444/5323 | 4137/4768 | 15 | 0.75 | 0.70–1.02 | 89.1% |
| ICI monotherapy + CT vs. Chemotherapy | 4480/4832 | 3568/4044 | 17 | **1.03** | **1.01–1.04** | 62.1% |
| Dual ICIs therapy vs. ICI monotherapy | 137/1795 | 1053/1595 | 6 | **1.17** | **1.04–1.31** | 87.7% |
| Dual ICIs therapy vs. Chemotherapy | 665/947 | 759/922 | 2 | 0.83 | 0.64–1.06 | 95.0% |
| Dual ICIs therapy + CT vs. Chemotherapy | 574/624 | 548/615 | 2 | 1.03 | 0.98–1.08 | 39.5% |
| ICI monotherapy vs. ICI monotherapy + CT | 256/391 | 159/172 | 1 |  |  |  |
| Dual ICIs vs. ICI monotherapy + CT | 442/576 | 159/172 | 1 |  |  |  |
| Dual ICIs + CT vs. ICI monotherapy + CT | 240/266 | 237/265 | 1 |  |  |  |
| **Grade 3-5 adverse events** |  |  |  |  |  |  |
| ICI monotherapy vs. Chemotherapy | 838/5323 | 2150/4768 | 15 | **0.33** | **0.27–0.40** | 87.1% |
| ICI monotherapy + CT vs. Chemotherapy | 2939/4832 | 2045/4044 | 17 | **1.15** | **1.07–1.24** | 74.5% |
| Dual ICIs therapy vs. ICI monotherapy | 492/1513 | 207/1314 | 5 | **1.97** | **1.26–3.07** | 87.6% |
| Dual ICIs therapy vs. Chemotherapy | 274/947 | 324/922 | 2 | 0.80 | 0.60–1.07 | 75.9% |
| Dual ICIs therapy + CT vs. Chemotherapy | 334/624 | 278/615 | 2 | **1.18** | **1.05–1.32** | 0.0% |
| ICI monotherapy vs. ICI monotherapy + CT | 76/391 | 96/172 | 1 |  |  |  |
| Dual ICIs therapy vs. ICI monotherapy + CT | 189/576 | 96/172 | 1 |  |  |  |
| Dual ICIs therapy + CT vs. ICI monotherapy + CT | 159/266 | 127/265 | 1 |  |  |  |
| **Grade 1-5 immune-related adverse events** |  |  |  |  |  |  |
| ICI monotherapy vs. Chemotherapy | 449/1800 | 115/1722 | 5 | **4.06** | **2.75–5.98** | 69.1% |
| ICI monotherapy + CT vs. Chemotherapy | 1377/3369 | 649/2745 | 12 | **2.02** | **1.63–2.52** | 85.2% |
| Dual ICIs therapy vs. ICI monotherapy | 206/544 | 146/546 | 2 | 1.48 | 0.77–2.84 | 92.5% |
| Dual ICIs therapy vs. Chemotherapy | 105/371 | 12/352 | 1 |  |  |  |
| Dual ICIs therapy + CT vs. Chemotherapy | 96/266 | 7/266 | 1 |  |  |  |
| Dual ICIs therapy + CT vs. ICI monotherapy + CT | 96/266 | 53/265 | 1 |  |  |  |
| **Grade 3-5 immune-related adverse events** |  |  |  |  |  |  |
| ICI monotherapy vs. Chemotherapy | 114/1800 | 18/1722 | 5 | **5.75** | **3.50–9.43** | 0.0% |
| ICI monotherapy + CT vs. Chemotherapy | 397/3170 | 92/2435 | 12 | **2.93** | **1.98–4.34** | 60.1% |
| Dual ICIs therapy vs. ICI monotherapy | 73/544 | 34/546 | 2 | **2.12** | **1.29–3.48** | 38.2% |
| Dual ICIs therapy vs. Chemotherapy | 44/371 | 3/352 | 1 |  |  |  |
| Dual ICIs therapy + CT vs. Chemotherapy | 36/266 | 1/266 | 1 |  |  |  |
| Dual ICIs therapy + CT vs. ICI monotherapy + CT | 36/266 | 13/265 | 1 |  |  |  |
| **Grade 1-5 colitis** |  |  |  |  |  |  |
| ICI monotherapy vs. Chemotherapy | 35/3822 | 3/3116 | 12 | **3.91** | **1.81–8.41** | 0.0% |
| ICI monotherapy + CT vs. Chemotherapy | 91/3489 | 9/2626 | 10 | **4.68** | **2.24–9.80** | 14.4% |
| Dual ICIs therapy vs. ICI monotherapy | 38/941 | 11/925 | 4 | **3.23** | **1.03–10.07** | 55.4% |
| Dual ICIs therapy vs. Chemotherapy | 12/371 | 0/352 | 1 |  |  |  |
| **Grade 3-5 colitis** |  |  |  |  |  |  |
| ICI monotherapy vs. Chemotherapy | 18/3822 | 1/3116 | 12 | **3.71** | **1.40–9.88** | 0.0% |
| ICI monotherapy + CT vs. Chemotherapy | 56/3627 | 6/2691 | 11 | **3.55** | **1.70–7.41** | 0.0% |
| Dual ICIs therapy vs. ICI monotherapy | 27/941 | 7/925 | 4 | **3.35** | **1.46–7.69** | 0.0% |
| Dual ICIs therapy vs. Chemotherapy | 7/371 | 0/352 | 1 |  |  |  |
| **Grade 1-5 diarrhea** |  |  |  |  |  |  |
| ICI monotherapy vs. Chemotherapy | 74/1920 | 159/1636 | 7 | **0.4** | **0.28–0.58** | 25.5% |
| ICI monotherapy + CT vs. Chemotherapy | 241/1370 | 91/1085 | 4 | **2.36** | **1.76–3.17** | 24.2% |
| Dual ICIs therapy vs. ICI monotherapy | 77/659 | 60/644 | 3 | 1.65 | 0.70–3.86 | 79.2% |
| Dual ICIs therapy vs. Chemotherapy | 17/371 | 1/352 | 1 |  |  |  |
| **Grade 3-5 diarrhea** |  |  |  |  |  |  |
| ICI monotherapy vs. Chemotherapy | 4/1920 | 13/1636 | 7 | 0.42 | 0.15–1.23 | 0.0% |
| ICI monotherapy + CT vs. Chemotherapy | 73/1508 | 12/1150 | 5 | **4.08** | **1.67–9.98** | 43.8% |
| Dual ICIs therapy vs. ICI monotherapy | 18/659 | 9/644 | 3 | 2.69 | 0.44–16.29 | 62.2% |
| Dual ICIs therapy vs. Chemotherapy | 8/371 | 1/352 | 1 |  |  |  |
| **Grade 1-5 pneumonitis** |  |  |  |  |  |  |
| ICI monotherapy vs. Chemotherapy | 163/3774 | 21/3092 | 11 | **5.08** | **2.75–9.40** | 33.9% |
| ICI monotherapy + CT vs. Chemotherapy | 186/3885 | 42/2835 | 13 | **2.81** | **2.00–3.95** | 0.0% |
| Dual ICIs therapy vs. ICI monotherapy | 74/941 | 28/925 | 4 | **2.57** | **1.68–3.93** | 0.0% |
| Dual ICIs therapy vs. Chemotherapy | 25/371 | 5/352 | 1 |  |  |  |
| Dual ICIs therapy + CT vs. Chemotherapy | 8/266 | 2/266 | 1 |  |  |  |
| **Grade 3-5 pneumonitis** |  |  |  |  |  |  |
| ICI monotherapy vs. Chemotherapy | 60/3774 | 8/3092 | 11 | **3.82** | **1.95–7.50** | 0.0% |
| ICI monotherapy + CT vs. Chemotherapy | 53/3885 | 19/2835 | 13 | **1.74** | **1.03–2.95** | 0.0% |
| Dual ICIs therapy vs. ICI monotherapy | 34/941 | 13/925 | 4 | **2.37** | **1.26–4.46** | 0.0% |
| Dual ICIs therapy vs. Chemotherapy | 11/371 | 2/352 | 1 |  |  |  |
| Dual ICIs therapy + CT vs. Chemotherapy | 3/266 | 1/266 | 1 |  |  |  |
| **Grade 1-5 hyperthyroidism** |  |  |  |  |  |  |
| ICI monotherapy vs. Chemotherapy | 131/3154 | 15/2479 | 9 | **6.00** | **3.59–10.01** | 0.0% |
| ICI monotherapy + CT vs. Chemotherapy | 174/4273 | 29/3196 | 14 | **3.49** | **2.23–5.45** | 14.5% |
| Dual ICIs therapy vs. ICI monotherapy | 59/941 | 30/925 | 4 | **1.88** | **1.22–2.89** | 0.0% |
| Dual ICIs therapy vs. Chemotherapy | 7/371 | 1/352 | 1 |  |  |  |
| Dual ICIs therapy + CT vs. Chemotherapy | 21/266 | 0/266 | 1 |  |  |  |
| **Grade 3-5 hyperthyroidism** |  |  |  |  |  |  |
| ICI monotherapy vs. Chemotherapy | 2/3154 | 0/2479 | 9 | 1.99 | 0.21–19.07 | 0.0% |
| ICI monotherapy + CT vs. Chemotherapy | 6/4273 | 0/3196 | 14 | 2.35 | 0.64–8.68 | 0.0% |
| Dual ICIs therapy vs. ICI monotherapy | 2/941 | 0/925 | 4 | 2.99 | 0.31–28.64 | 0.0% |
| Dual ICIs therapy vs. Chemotherapy | 1/371 | 0/352 | 1 |  |  |  |
| Dual ICIs therapy + CT vs. Chemotherapy | 1/266 | 0/266 | 1 |  |  |  |
| **Grade 1-5 hypothyroidism** |  |  |  |  |  |  |
| ICI monotherapy vs. Chemotherapy | 277/3678 | 18/2973 | 11 | **9.23** | **5.91–14.43** | 0.0% |
| ICI monotherapy + CT vs. Chemotherapy | 378/4273 | 42/3196 | 14 | **5.44** | **3.38–8.77** | 47.5% |
| Dual ICIs therapy vs. ICI monotherapy | 99/941 | 71/925 | 4 | 1.35 | 0.98–1.87 | 12.5% |
| Dual ICIs therapy vs. Chemotherapy | 28/371 | 2/352 | 1 |  |  |  |
| Dual ICIs therapy + CT vs. Chemotherapy | 24/266 | 2/266 | 1 |  |  |  |
| **Grade 3-5 hypothyroidism** |  |  |  |  |  |  |
| ICI monotherapy vs. Chemotherapy | 4/3678 | 0/2973 | 11 | 3.42 | 0.56–20.96 | 0.0% |
| ICI monotherapy + CT vs. Chemotherapy | 13/4273 | 0/3196 | 14 | **3.05** | **1.02–9.13** | 0.0% |
| Dual ICIs therapy vs. ICI monotherapy | 5/941 | 3/925 | 4 | 1.49 | 0.38–5.80 | 0.0% |
| Dual ICIs therapy vs. Chemotherapy | 3/371 | 0/352 | 1 |  |  |  |
| Dual ICIs therapy + CT vs. Chemotherapy | 3/266 | 0/266 | 1 |  |  |  |
| **Grade 1-5 thyroiditis** |  |  |  |  |  |  |
| ICI monotherapy vs. Chemotherapy | 18/2096 | 0/1498 | 5 | **4.76** | **1.23–18.40** | 0.0% |
| ICI monotherapy + CT vs. Chemotherapy | 8/953 | 0/752 | 3 | 4.87 | 0.85–27.82 | 0.0% |
| Dual ICIs therapy vs. ICI monotherapy | 6/455 | 4/458 | 2 | 1.5 | 0.42–5.31 | 0.0% |
| Dual ICIs therapy + CT vs. Chemotherapy | 3/266 | 0/266 | 1 |  |  |  |
| **Grade 3-5 thyroiditis** |  |  |  |  |  |  |
| ICI monotherapy vs. Chemotherapy | 0/2096 | 0/1498 | 5 |  |  |  |
| ICI monotherapy + CT vs. Chemotherapy | 1/953 | 0/752 | 3 | 3.02 | 0.12–73.85 | 0.0% |
| Dual ICIs therapy vs. ICI monotherapy | 1/455 | 1/458 | 2 | 1.01 | 0.11–9.66 | 0.0% |
| Dual ICIs therapy + CT vs. Chemotherapy | 0/266 | 0/266 | 1 |  |  |  |
| **Grade 1-5 hypophysitis** |  |  |  |  |  |  |
| ICI monotherapy vs. Chemotherapy | 6/1841 | 0/1426 | 4 | 3.62 | 0.62–21.13 | 0.0% |
| ICI monotherapy + CT vs. Chemotherapy | 14/2143 | 1/1921 | 7 | **4.06** | **1.27–12.95** | 0.0% |
| Dual ICIs therapy vs. ICI monotherapy | 13/826 | 3/827 | 3 | **3.47** | **1.15–10.49** | 0.0% |
| Dual ICIs therapy vs. Chemotherapy | 2/371 | 0/352 | 1 |  |  |  |
| Dual ICIs therapy+CT vs. Chemotherapy | 3/266 | 0/266 | 1 |  |  |  |
| **Grade 3-5 hypophysitis** |  |  |  |  |  |  |
| ICI monotherapy vs. Chemotherapy | 6/1841 | 0/1426 | 4 | 3.62 | 0.62–21.13 | 0.0% |
| ICI monotherapy + CT vs. Chemotherapy | 7/2281 | 1/1986 | 8 | 2.86 | 0.70–11.75 | 0.0% |
| Dual ICIs therapy vs. ICI monotherapy | 4/826 | 0/827 | 3 | 3.62 | 0.59–22.13 | 0.0% |
| Dual ICIs therapy vs. Chemotherapy | 1/371 | 0/352 | 1 |  |  |  |
| Dual ICIs therapy + CT vs. Chemotherapy | 1/266 | 0/266 | 1 |  |  |  |
| **Grade 1-5 diabetes** |  |  |  |  |  |  |
| ICI monotherapy vs. Chemotherapy | 5/1829 | 1/1235 | 5 | 1.46 | 0.31–7.00 | 0.0% |
| ICI monotherapy + CT vs. Chemotherapy | 20/2328 | 4/1535 | 6 | 2.27 | 0.87–5.91 | 0.0% |
| Dual ICIs therapy vs. ICI monotherapy | 4/653 | 0/650 | 2 | 4.98 | 0.58–42.49 | 0.0% |
| Dual ICIs therapy vs. Chemotherapy | 2/371 | 0/352 | 1 |  |  |  |
| Dual ICIs therapy + CT vs. Chemotherapy | 2/266 | 0/266 | 1 |  |  |  |
| **Grade 3-5 diabetes** |  |  |  |  |  |  |
| ICI monotherapy vs. Chemotherapy | 3/1829 | 0/1235 | 5 | 2.56 | 0.28–23.07 | 0.0% |
| ICI monotherapy + CT vs. Chemotherapy | 14/2328 | 2/1535 | 6 | 2.43 | 0.78–7.53 | 0.0% |
| Dual ICIs therapy vs. ICI monotherapy | 3/653 | 0/650 | 2 | 3.91 | 0.43–35.28 | 0.0% |
| Dual ICIs therapy vs. Chemotherapy | 1/371 | 0/352 | 1 |  |  |  |
| Dual ICIs therapy + CT vs. Chemotherapy | 2/266 | 0/266 | 1 |  |  |  |
| **Grade 1-5 pruritus** |  |  |  |  |  |  |
| ICI monotherapy vs. Chemotherapy | 58/803 | 11/577 | 4 | **2.93** | **1.30–6.60** | 29.6% |
| ICI monotherapy + CT vs. Chemotherapy | 120/1370 | 20/1085 | 4 | **3.64** | **1.33–9.99** | 70.8% |
| Dual ICIs therapy vs. ICI monotherapy | 16/115 | 11/98 | 1 |  |  |  |
| **Grade 3-5 pruritus** |  |  |  |  |  |  |
| ICI monotherapy vs. Chemotherapy | 0/803 | 0/577 | 4 |  |  |  |
| ICI monotherapy + CT vs. Chemotherapy | 8/1370 | 0/1085 | 4 | 4.69 | 0.82–26.79 | 0.0% |
| Dual ICIs therapy vs. ICI monotherapy | 1/115 | 0/98 | 1 |  |  |  |
| **Grade 1-5 rash** |  |  |  |  |  |  |
| ICI monotherapy vs. Chemotherapy | 123/1765 | 41/1510 | 6 | **2.29** | **1.33–3.93** | 41.6% |
| ICI monotherapy + CT vs. Chemotherapy | 466/2790 | 152/2155 | 8 | **2.56** | **1.54–4.24** | 83.1% |
| Dual ICIs therapy vs. ICI monotherapy | 32/486 | 7/467 | 2 | **4.06** | **1.79–9.22** | 0.0% |
| Dual ICIs therapy vs. Chemotherapy | 16/371 | 2/352 | 1 |  |  |  |
| Dual ICIs therapy + CT vs. Chemotherapy | 19/266 | 2/266 | 1 |  |  |  |
| **Grade 3-5 rash** |  |  |  |  |  |  |
| ICI monotherapy vs. Chemotherapy | 13/1765 | 4/1510 | 6 | **2.29** | **1.33–3.93** | 41.6% |
| ICI monotherapy + CT vs. Chemotherapy | 59/2928 | 10/2220 | 9 | **3.29** | **1.17–9.28** | 43.1% |
| Dual ICIs therapy vs. ICI monotherapy | 4/486 | 4/467 | 2 | 1.00 | 0.14–7.14 | 32.3% |
| Dual ICIs therapy vs. Chemotherapy | 2/371 | 0/352 | 1 |  |  |  |
| Dual ICIs therapy + CT vs. Chemotherapy | 6/266 | 0/266 | 1 |  |  |  |
| **Grade 1-5 severe skin reaction** |  |  |  |  |  |  |
| ICI monotherapy vs. Chemotherapy | 32/1472 | 4/1074 | 3 | **4.88** | **1.82–13.09** | 0.0% |
| ICI monotherapy + CT vs. Chemotherapy | 24/1834 | 10/1301 | 6 | 1.52 | 0.73–3.18 | 0.0% |
| Dual ICIs therapy vs. ICI monotherapy | 11/282 | 6/281 | 1 |  |  |  |
| **Grade 3-5 severe skin reaction** |  |  |  |  |  |  |
| ICI monotherapy vs. Chemotherapy | 13/1765 | 4/1510 | 6 | **2.29** | **1.33–3.93** | 41.6% |
| ICI monotherapy + CT vs. Chemotherapy | 59/2928 | 10/2220 | 9 | **3.29** | **1.17–9.28** | 43.1% |
| Dual ICIs therapy vs. ICI monotherapy | 10/282 | 4/281 | 1 |  |  |  |
| **Grade 1-5 myocarditis** |  |  |  |  |  |  |
| ICI monotherapy vs. Chemotherapy | 1/636 | 0/615 | 1 |  |  |  |
| ICI monotherapy + CT vs. Chemotherapy | 1/779 | 2/763 | 3 | 0.60 | 0.07–4.89 | 0.0% |
| Dual ICIs therapy vs. ICI monotherapy | 3/282 | 1/281 | 1 |  |  |  |
| Dual ICIs therapy + CT vs. Chemotherapy | 1/266 | 0/266 | 1 |  |  |  |
| **Grade 3-5 myocarditis** |  |  |  |  |  |  |
| ICI monotherapy vs. Chemotherapy | 1/636 | 0/615 | 1 |  |  |  |
| ICI monotherapy + CT vs. Chemotherapy | 1/779 | 1/763 | 3 | 0.94 | 0.06–14.98 | 0.0% |
| Dual ICIs therapy vs. ICI monotherapy | 2/282 | 0/281 | 1 |  |  |  |
| Dual ICIs therapy + CT vs. Chemotherapy | 1/266 | 0/266 | 1 |  |  |  |
| **Grade 1-5 nephritis** |  |  |  |  |  |  |
| ICI monotherapy vs. Chemotherapy | 6/1645 | 0/1588 | 5 | 3.70 | 0.77–17.68 | 0.0% |
| ICI monotherapy + CT vs. Chemotherapy | 18/2106 | 5/1551 | 7 | 1.99 | 0.76–5.25 | 0.0% |
| Dual ICIs therapy vs. ICI monotherapy | 5/653 | 5/650 | 2 | **1.11** | **1.17–7.33** | 34.9% |
| Dual ICIs therapy vs. Chemotherapy | 2/371 | 0/352 | 1 |  |  |  |
| **Grade 3-5 nephritis** |  |  |  |  |  |  |
| ICI monotherapy vs. Chemotherapy | 4/1645 | 0/1588 | 5 | 2.92 | 0.59–14.41 | 0.0% |
| ICI monotherapy + CT vs. Chemotherapy | 14/2106 | 2/1551 | 7 | 2.71 | 0.82–8.98 | 0.0% |
| Dual ICIs therapy vs. ICI monotherapy | 1/653 | 1/650 | 2 | 1 | 0.06–15.85 | 0.0% |
| Dual ICIs therapy vs. Chemotherapy | 0/371 | 0/352 | 1 |  |  |  |
| **Grade 1-5 hepatitis** |  |  |  |  |  |  |
| ICI monotherapy vs. Chemotherapy | 18/2520 | 1/2048 | 7 | **3.20** | **1.03–9.97** | 0.0% |
| ICI monotherapy + CT vs. Chemotherapy | 74/2928 | 21/2093 | 10 | **2.16** | **1.35–3.46** | 0.0% |
| Dual ICIs therapy vs. ICI monotherapy | 18/826 | 8/827 | 3 | 2.11 | 0.64–6.91 | 38.2% |
| Dual ICIs therapy vs. Chemotherapy | 9/371 | 0/352 | 1 |  |  |  |
| Dual ICIs therapy + CT vs. Chemotherapy | 11/266 | 0/266 | 1 |  |  |  |
| **Grade 3-5 hepatitis** |  |  |  |  |  |  |
| ICI monotherapy vs. Chemotherapy | 10/2520 | 0/2048 | 7 | 3.88 | 0.82–18.31 | 0.0% |
| ICI monotherapy + CT vs. Chemotherapy | 35/2928 | 2/2093 | 10 | **4.08** | **1.69–9.84** | 0.0% |
| Dual ICIs therapy vs. ICI monotherapy | 15/826 | 6/827 | 3 | 2.12 | 0.77–5.87 | 3.3% |
| Dual ICIs therapy vs. Chemotherapy | 8/371 | 0/352 | 1 |  |  |  |
| Dual ICIs therapy + CT vs. Chemotherapy | 8/266 | 0/266 | 1 |  |  |  |
| **Grade 1-5 myositis** |  |  |  |  |  |  |
| ICI monotherapy vs. Chemotherapy | 6/836 | 1/459 | 2 | 2.46 | 0.41–14.83 | 0.0% |
| ICI monotherapy + CT vs. Chemotherapy | 3/965 | 1/763 | 3 | 1.58 | 0.27–9.08 | 0.0% |
| Dual ICIs therapy vs. ICI monotherapy | 4/455 | 3/458 | 2 | 1.35 | 0.30–6.03 | 0.0% |
| **Grade 3-5 myositis** |  |  |  |  |  |  |
| ICI monotherapy vs. Chemotherapy | 0/836 | 0/459 | 2 |  |  |  |
| ICI monotherapy + CT vs. Chemotherapy | 1/965 | 0/763 | 3 | 3.00 | 0.12–73.25 | 0.0% |
| Dual ICIs therapy vs. ICI monotherapy | 3/455 | 0/458 | 2 | 6.98 | 0.36–134.42 | 0.0% |
| **Grade 1-5 hypersensitivity/infusion reaction** |  |  |  |  |  |  |
| ICI monotherapy vs. Chemotherapy | 123/2773 | 81/2474 | 9 | 1.10 | 0.44–2.73 | 84.1% |
| ICI monotherapy + CT vs. Chemotherapy | 54/2290 | 29/1713 | 7 | 1.33 | 0.80–2.20 | 5.3% |
| Dual ICIs therapy vs. ICI monotherapy | 48/1309 | 50/1128 | 4 | 0.87 | 0.52–1.46 | 37.7% |
| Dual ICIs therapy + CT vs. Chemotherapy | 18/358 | 4/349 | 1 |  |  |  |
| **Grade 3-5 hypersensitivity/infusion reaction** |  |  |  |  |  |  |
| ICI monotherapy vs. Chemotherapy | 8/2773 | 15/2474 | 9 | 0.49 | 0.17–1.43 | 19.7% |
| ICI monotherapy + CT vs. Chemotherapy | 15/2428 | 4/1778 | 8 | 2.11 | 0.80–5.58 | 0.0% |
| Dual ICIs therapy vs. ICI monotherapy | 9/1309 | 4/1128 | 4 | 1.78 | 0.51–6.28 | 0.0% |
| Dual ICIs therapy + CT vs. Chemotherapy | 2/358 | 2/349 | 1 |  |  |  |

ICI: immune checkpoint inhibitor; CT: chemotherapy; vs.: versus; RR: relative risks; 95% CIs: 95% confidence intervals.

# **Table S8. Sensitivity analysis in pair-wise meta-analysis**

| **Omitted studies** | **RR (95%CI)** | **RR (95%CI)** |
| --- | --- | --- |
| **Adverse events** | **Grade 1-5** | **Grade 3-5** |
| **ICI monotherapy vs. Chemotherapy** |  |  |
| CheckMate 057, 2015 | 0.74 (0.69–0.81) | 0.34 (0.28–0.42) |
| CheckMate 017, 2015 | 0.75 (0.70–0.81) | 0.35 (0.28–0.42) |
| POPLAR, 2016 | 0.75 (0.69–0.80) | 0.33 (0.27–0.41) |
| KEYNOTE-010, 2016 | 0.75 (0.69–0.80) | 0.32 (0.26–0.40) |
| KEYNOTE 024, 2016 | 0.74 (0.69–0.80) | 0.32 (0.26–0.40) |
| CheckMate 026, 2017 | 0.75 (0.69–0.81) | 0.33 (0.26–0.41) |
| OAK, 2017 | 0.75 (0.69–0.81) | 0.33 (0.26–0.41) |
| JAVELIN Lung 200, 2018 | 0.75 (0.69–0.81) | 0.34 (0.28–0.42) |
| CheckMate 227, 2019 | 0.74 (0.69–0.80) | 0.32 (0.26–0.40) |
| KEYNOTE-042, 2019 | 0.75 (0.70–0.81) | 0.32 (0.26–0.40) |
| CheckMate 078, 2019 | 0.75 (0.69–0.81) | 0.34 (0.28–0.42) |
| IMpower110, 2020 | 0.74 (0.70–0.77) | 0.32 (0.26–0.38) |
| MYSTIC, 2020 | 0.76 (0.70–0.81) | 0.32 (0.26–0.40) |
| CheckMate 331, 2021 | 0.76 (0.71–0.81) | 0.34 (0.28–0.42) |
| EMPOWER-Lung 1, 2021 | 0.76 (0.70–0.81) | 0.33 (0.26–0.41) |
| **ICI monotherapy+CT vs. Chemotherapy** |  |  |
| CA184-041, 2012 | 1.03 (1.01–1.05) | 1.14 (1.06–1.24) |
| CA184-041, 2013 | 1.03 (1.01–1.05) | 1.15 (1.06–1.25) |
| KEYNOTE-021, 2016 | 1.03 (1.01–1.05) | 1.14 (1.06–1.24) |
| CA184-156, 2016 | 1.03 (1.01–1.05) | 1.16 (1.07–1.26) |
| Study 104, 2017 | 1.03 (1.01–1.05) | 1.13 (1.05–1.22) |
| KEYNOTE-189, 2018 | 1.03 (1.01–1.06) | 1.16 (1.07–1.26) |
| IMpower133, 2018 | 1.03 (1.01–1.05) | 1.16 (1.07–1.26) |
| KEYNOTE-407, 2018 | 1.03 (1.01–1.06) | 1.16 (1.07–1.26) |
| CheckMate 227, 2019 | 1.02 (1.00–1.04) | 1.13 (1.05–1.21) |
| IMpower130, 2019 | 1.03 (1.01–1.05) | 1.15 (1.06–1.24) |
| IMpower131, 2020 | 1.03 (1.01–1.05) | 1.16 (1.07–1.26) |
| IMpower132, 2020 | 1.03 (1.01–1.05) | 1.14 (1.05–1.23) |
| KEYNOTE-604, 2020 | 1.03 (1.01–1.05) | 1.16 (1.07–1.26) |
| ORIENT-11, 2020 | 1.03 (1.01–1.05) | 1.16 (1.07–1.26) |
| CameL, 2020 | 1.03 (1.01–1.05) | 1.13 (1.05–1.22) |
| CASPIAN, 2021 | 1.03 (1.01–1.05) | 1.17 (1.08–1.26) |
| RATIONALE 307, 2021 | 1.03 (1.01–1.05) | 1.16 (1.07–1.26) |
| **Dual ICIs therapy vs. ICI monotherapy** |  |  |
| CheckMate 032, 2016 | 1.14 (0.99–1.30) | 1.99 (1.18–3.36) |
| CheckMate 227, 2019 | 1.17 (0.98–1.39) | 2.04 (1.09–3.84) |
| ARCTIC, 2020 | 1.21 (1.03–1.42) | 2.17 (1.29–3.66) |
| MYSTIC, 2020 | 1.18 (1.00–1.40) | 2.09 (1.17–3.75) |
| CheckMate 451, 2021 | 1.12 (1.00–1.25) | 1.59 (1.35–1.88) |
| KEYNOTE-598, 2021 | 1.20 (1.06–1.35) | – |
| **Immune-related adverse events** | **Grade 1-5** | **Grade 3-5** |
| **ICI monotherapy vs. Chemotherapy** |  |  |
| KEYNOTE 024, 2016 | 3.78 (2.49–5.74) | 5.41 (3.25–9.12) |
| KEYNOTE-042, 2019 | 4.32 (2.39–7.82) | 6.03 (2.99–12.14) |
| IMpower110, 2020 | 4.58 (2.41–6.15) | 6.20 (3.54–10.84) |
| MYSTIC, 2020 | 4.17 (2.57–6.75) | 5.88 (3.42–10.11) |
| EMPOWER-Lung 1, 2021 | 3.59 (2.47–5.22) | 5.47 (3.28–9.11) |
| **ICI monotherapy+CT vs. Chemotherapy** |  |  |
| KEYNOTE-021, 2016 | 2.04 (1.62–2.58) | 2.95 (1.96–4.46) |
| CA184-156, 2016 | 2.05 (1.59–2.64) | 2.54 (1.84–3.51) |
| Study 104, 2017 | 2.15 (1.68–2.75) | 2.76 (1.76–4.34) |
| KEYNOTE-189, 2018 | 2.05 (1.61–2.61) | 3.06 (1.98–4.73) |
| IMpower133, 2018 | 2.10 (1.63–2.69) | – |
| KEYNOTE-407, 2018 | 1.93 (1.55–2.41) | 2.88 (1.84–4.50) |
| IMpower130, 2019 | 2.00 (1.58–2.53) | 3.01 (1.95–4.65) |
| PROLUNG, 2020 | 1.95 (1.55–2.44) | 2.90 (1.93–4.37) |
| IMpower132, 2020 | 2.16 (1.67–2.79) | 3.05 (1.94–4.77) |
| KEYNOTE-604, 2020 | 2.01 (1.59–2.54) | – |
| ORIENT-11, 2020 | 2.16 (1.70–2.76) | 3.31 (2.31–4.74) |
| CASPIAN, 2021 | 1.88 (1.53–2.32) | 2.79 (1.88–4.16) |
| CA184-041, 2012 | – | 2.94 (1.91–4.52) |
| CA184-041, 2013 | – | 3.01 (1.97–4.62) |

ICI: immune checkpoint inhibitor; CT: chemotherapy; RR: relative risks; 95% CIs: 95% confidence intervals.

# **Table S9.** Univariable meta-regression for primary outcomes

| ***P* value** | | | | | | | |
| --- | --- | --- | --- | --- | --- | --- | --- |
| **Outcomes** | **Age** | **Male** | **PS score** | **Brain metastasis** | **Liver metastasis** | **Current/former Smoker** | **Previous radiotherapy** |
| **Grade 1-5 AE** |  |  |  |  |  |  |  |
| ICI monotherapy vs. CT | 0.678 | 0.977 | 0.984 | – | – | 0.908 | 0.723 |
| ICI monotherapy + CT vs. CT | 0.539 | 0.362 | 0.693 | 0.943 | 0.612 | 0.411 | 0.987 |
| Dual ICIs therapy vs. ICI monotherapy | 0.721 | 0.350 | 0.775 | 0.667 | – | 0.648 | – |
| **Grade 3-5 AE** |  |  |  |  |  |  |  |
| ICI monotherapy vs. CT | 0.715 | 0.979 | 0.912 | 0.838 | – | 0.948 | 0.594 |
| ICI monotherapy + CT vs. CT | 0.560 | 0.732 | 0.904 | 0.835 | 0.798 | 0.897 | 0.918 |
| Dual ICIs therapy vs. ICI monotherapy | 0.744 | 0.633 | 0.752 | – | – | 0.817 | – |
| **Grade 1-5 irAE** |  |  |  |  |  |  |  |
| ICI monotherapy vs. CT | 0.770 | 0.915 | 0.108 | 0.626 | – | 0.620 | – |
| ICI monotherapy + CT vs. CT | 0.635 | 0.287 | 0.373 | 0.810 | 0.526 | 0.857 | 0.633 |
| **Grade 3-5 irAE** |  |  |  |  |  |  |  |
| ICI monotherapy vs. CT | 0.698 | 0.926 | 0.108 | 0.570 | – | 0.481 | – |
| ICI monotherapy + CT vs. CT | 0.449 | 0.587 | 0.531 | 0.965 | 0.592 | 0.916 | 0.916 |

AE: adverse event; irAE: immune-related adverse event; ICI: immune checkpoint inhibitor; CT: chemotherapy.

# **Table S10. Trim and fill method to deal with publication bias**

| Outcome | Publication bias | | Before trim and fill | | After trim and fill | | P for  interaction |
| --- | --- | --- | --- | --- | --- | --- | --- |
|  | P for  Egger’s test | P for  Begg’s test | No. of  studies | RR (95%Cl) | No. of studies | RR (95%Cl) |  |
| Grade 1-5 AE (ICI monotherapy vs. CT) | **0.014** | 0.428 | 15 | 0.75 (0.70–1.02) | 17 | **0.78 (0.70–0.95)** | 1.00 |
| Grade 3-5 AE (ICI monotherapy vs. CT) | **0.006** | **0.018** | 15 | **0.33 (0.27–0.40)** | 19 | **0.35 (0.33–0.38)** | 0.58 |
| Grade 1-5 AE (ICI monotherapy + CT vs. CT) | **0.014** | 0.266 | 17 | **1.03 (1.01–1.04)** | 23 | 1.01 (0.99–1.02) | 0.07 |
| Grade 3-5 AE (ICI monotherapy + CT vs. CT) | 0.130 | 0.091 | - | | | |  |
| Grade 1-5 irAE (ICI monotherapy + CT vs. CT) | **0.016** | 0.064 | 12 | **1.84 (1.11–1.86)** | 14 | **1.81 (1.45–2.26)** | 0.92 |
| Grade 3-5 irAE (ICI monotherapy + CT vs. CT) | 0.805 | 0.732 | - | | | |  |

AE: adverse event; irAE: immune-related adverse event; ICI: immune checkpoint inhibitor; CT: chemotherapy; RR: relative risks; 95% CIs: 95% confidence intervals.

# **Table S11. Pooled incidence of adverse events**

| **Treatment** | **Case/No.** | **No. of studies** | **Pooled incidence** | **95%CI** | ***I^2^*** |
| --- | --- | --- | --- | --- | --- |
| **Grade 1-5 adverse events** | | | | | |
| Chemotherapy | 7560/8591 | 32 | 88.35% | 86.19%–90.51% | 91.4% |
| ICI monotherapy | 4041/6158 | 19 | 65.99% | 59.80%–72.18% | 96.8% |
| ICI monotherapy + CT | 4380/4732 | 17 | 93.21% | 91.20%–95.22% | 94.5% |
| Dual ICIs therapy | 1371/1795 | 6 | 76.47% | 64.15%–88.78% | 98.1% |
| Dual ICIs therapy + CT | 574/624 | 2 | 92.02% | 89.05%–94.99% | 46.3% |
| **Grade 3-5 adverse events** | | | | | |
| Chemotherapy | 4128/8591 | 32 | 49.75% | 45.52%–53.99% | 93.9% |
| ICI monotherapy | 914/5877 | 18 | 15.22% | 12.91%–17.53% | 84.3% |
| ICI monotherapy + CT | 2939/4832 | 17 | 59.96% | 54.52%–65.39% | 90.2% |
| Dual ICIs therapy | 492/1513 | 5 | 31.38% | 20.32%–42.44% | 95.5% |
| Dual ICIs therapy + CT | 334/624 | 2 | 54.23% | 43.56%–64.90% | 86.5% |
| **Grade 1-5 immune-related adverse events** | | |  |  |  |
| Chemotherapy | 764/4467 | 17 | 17.04% | 11.80%–22.28% | 97.7% |
| ICI monotherapy | 545/1977 | 6 | 30.14% | 19.79%–40.48% | 96.6% |
| ICI monotherapy + CT | 1377/3369 | 12 | 41.60% | 31.06%–52.14% | 97.8% |
| Dual ICIs therapy | 206/544 | 2 | 43.20% | 13.72%–72.67% | 97.8% |
| Dual ICIs therapy + CT | 96/266 | 1 | 36.09% |  |  |
| **Grade 3-5 immune-related adverse events** | | |  |  |  |
| Chemotherapy | 110/4157 | 17 | 2.27% | 1.44%–3.09% | 77.8% |
| ICI monotherapy | 132/1977 | 6 | 6.59% | 4.52%–8.67% | 71.4% |
| ICI monotherapy + CT | 397/3170 | 12 | 11.42% | 7.77%–15.06% | 91.8% |
| Dual ICIs therapy | 73/544 | 2 | 13.77% | 9.09%–18.46% | 54.7% |
| Dual ICIs therapy + CT | 36/266 | 1 | 13.53% |  |  |
| **Grade 1-5 colitis** |  |  |  |  |  |
| Chemotherapy | 12/5742 | 22 | 0.33% | 0.11%–0.55% | 0.0% |
| ICI monotherapy | 44/4378 | 15 | 0.79% | 0.52%–1.05% | 0.0% |
| ICI monotherapy + CT | 91/3489 | 10 | 2.19% | 1.35%–3.02% | 67.8% |
| Dual ICIs therapy | 38/941 | 4 | 3.66% | 2.15%–5.17% | 34.3% |
| **Grade 3-5 colitis** |  |  |  |  |  |
| Chemotherapy | 7/5807 | 23 | 0.25% | 0.01%–0.51% | 5.6% |
| ICI monotherapy | 24/4378 | 15 | 0.55% | 0.31%–0.80% | 0.0% |
| ICI monotherapy + CT | 56/3627 | 11 | 1.18% | 0.66%–1.70% | 55.9% |
| Dual ICIs therapy | 27/941 | 4 | 2.53% | 1.24%–3.83% | 52.6% |
| **Grade 1-5 diarrhea** |  |  |  |  |  |
| Chemotherapy | 250/2721 | 11 | 8.26% | 5.62%–10.89% | 96.2% |
| ICI monotherapy | 127/2195 | 9 | 6.02% | 3.37%–8.68% | 93.2% |
| ICI monotherapy + CT | 241/1370 | 4 | 14.39% | 2.68%–26.10% | 98.7% |
| Dual ICIs therapy | 77/659 | 3 | 14.94% | 2.17%–27.70% | 94.8% |
| **Grade 3-5 diarrhea** |  |  |  |  |  |
| Chemotherapy | 25/2786 | 12 | 0.70% | 0.32%–1.08% | 22.3% |
| ICI monotherapy | 12/2195 | 9 | 0.57% | 0.01%–1.25% | 60.9% |
| ICI monotherapy + CT | 73/1408 | 5 | 5.38% | 2.24%–8.62% | 84.2% |
| Dual ICIs therapy | 18/659 | 3 | 2.59% | 1.38%–3.80% | 0.0% |
| **Grade 1-5 pneumonitis** |  |  |  |  |  |
| Chemotherapy | 63/5927 | 24 | 0.99% | 0.63%–1.35% | 42.3% |
| ICI monotherapy | 183/4330 | 14 | 3.67% | 2.65%–4.68% | 69.9% |
| ICI monotherapy + CT | 186/3885 | 13 | 4.52% | 2.76%–6.28% | 89.4% |
| Dual ICIs therapy | 74/941 | 4 | 7.01% | 3.67%–10.36% | 76.1% |
| Dual ICIs therapy + CT | 8/266 | 1 | 3.01% |  |  |
| **Grade 3-5 pneumonitis** |  |  |  |  |  |
| Chemotherapy | 27/5927 | 24 | 0.37% | 0.19%–0.55% | 0.0% |
| ICI monotherapy | 68/4330 | 14 | 1.25% | 0.75%–1.75% | 56.2% |
| ICI monotherapy + CT | 53/3885 | 13 | 1.31% | 0.70%–1.91% | 63.9% |
| Dual ICIs therapy | 34/941 | 4 | 3.20% | 1.76%–4.64% | 37.2% |
| Dual ICIs therapy + CT | 3/266 | 1 | 1.13% |  |  |
| **Grade 1-5 hyperthyroidism** | |  |  |  |  |
| Chemotherapy | 44/5675 | 23 | 0.68% | 0.42%–0.93% | 7.3% |
| ICI monotherapy | 157/3710 | 12 | 3.76% | 2.53%–0.50% | 76.5% |
| ICI monotherapy + CT | 174/4273 | 14 | 3.97% | 3.03%–4.91% | 59.9% |
| Dual ICIs therapy | 59/941 | 4 | 7.06% | 2.20%–11.92% | 88.8% |
| Dual ICIs therapy + CT | 21/266 | 1 | 7.89% |  |  |
| **Grade 3-5 hyperthyroidism** | |  |  |  |  |
| Chemotherapy | 0/5675 | 23 | 0 |  |  |
| ICI monotherapy | 2/3710 | 12 | 0.15% | 0.06%–0.37% | 0.0% |
| ICI monotherapy + CT | 6/4273 | 14 | 0.22% | 0.03%–0.40% | 0.0% |
| Dual ICIs therapy | 2/941 | 4 | 0.30% | 0.02%–0.72% | 0.0% |
| Dual ICIs therapy + CT | 1/266 | 1 | 0.38% |  |  |
| **Grade 1-5 hypothyroidism** |  |  |  |  |  |
| Chemotherapy | 60/6169 | 25 | 0.99% | 0.63%–1.34% | 31.5% |
| ICI monotherapy | 327/4234 | 14 | 6.77% | 5.26%–8.28% | 73.8% |
| ICI monotherapy + CT | 378/4273 | 14 | 9.27% | 6.82%–11.72% | 89.4% |
| Dual ICIs therapy | 99/941 | 4 | 10.49% | 6.72%–14.26% | 71.3% |
| Dual ICIs therapy + CT | 24/266 | 1 | 9.02% |  |  |
| **Grade 3-5 hypothyroidism** |  |  |  |  |  |
| Chemotherapy | 0/6169 | 25 | 0 |  |  |
| ICI monotherapy | 5/4234 | 14 | 0.24% | 0.01%–0.47% | 0.0% |
| ICI monotherapy + CT | 13/4273 | 14 | 0.36% | 0.14%–0.57% | 0.0% |
| Dual ICIs therapy | 5/941 | 4 | 0.55% | 0.03%–1.07% | 0.0% |
| Dual ICIs therapy + CT | 3/266 | 1 |  |  |  |
| **Grade 1-5 thyroiditis** |  |  |  |  |  |
| Chemotherapy | 0/2250 | 8 | 0 |  |  |
| ICI monotherapy | 22/2554 | 7 | 0.60% | 0.21%–0.99% | 39.5% |
| ICI monotherapy + CT | 8/953 | 3 | 0.71% | 0.08%–1.50% | 47.2% |
| Dual ICIs therapy | 6/455 | 2 | 1.06% | 0.09%–2.20% | 32.9% |
| Dual ICIs therapy + CT | 3/266 | 1 | 1.13% |  |  |
| **Grade 3-5 thyroiditis** |  |  |  |  |  |
| Chemotherapy | 0/2250 | 8 | 0 |  |  |
| ICI monotherapy | 1/2554 | 7 | 0.56% | 0.04%–1.66% | 0.0% |
| ICI monotherapy + CT | 1/953 | 3 | 0.36% | 0.03%–1.06% | 0.0% |
| Dual ICIs therapy | 1/455 | 2 | 0.35% | 0.03%–1.04% | 0.0% |
| Dual ICIs therapy + CT | 0/266 | 1 | 0 |  |  |
| **Grade 1-5 hypophysitis** |  |  |  |  |  |
| Chemotherapy | 1/3347 | 11 | 0.21% | 0.02%–0.62% | 0.0% |
| ICI monotherapy | 9/2299 | 6 | 0.42% | 0.12%–0.72% | 0.0% |
| ICI monotherapy + CT | 14/2143 | 7 | 0.65% | 0.29%–1.02% | 0.0% |
| Dual ICIs therapy | 13/826 | 3 | 1.19% | 0.02%–2.50% | 70.7% |
| Dual ICIs therapy + CT | 3/266 | 1 | 1.13% |  |  |
| **Grade 3-5 hypophysitis** |  |  |  |  |  |
| Chemotherapy | 1/3412 | 12 | 0.21% | 0.02%–0.62% | 0.0% |
| ICI monotherapy | 6/2299 | 6 | 0.37% | 0.06%–0.69% | 0.0% |
| ICI monotherapy + CT | 7/2281 | 8 | 0.54% | 0.12%–0.96% | 0.0% |
| Dual ICIs therapy | 4/826 | 3 | 0.40% | 0.03%–0.83% | 0.0% |
| Dual ICIs therapy + CT | 1/266 | 1 | 0.38% |  |  |
| **Grade 1-5 diabetes** |  |  |  |  |  |
| Chemotherapy | 5/2770 | 11 | 0.39% | 0.02%–0.75% | 0.0% |
| ICI monotherapy | 5/2110 | 6 | 0.40% | 0.04%–0.77% | 0.0% |
| ICI monotherapy + CT | 20/2328 | 6 | 0.66% | 0.29%–1.02% | 16.4% |
| Dual ICIs therapy | 4/653 | 2 | 0.60% | 0.01%–1.20% | 0.0% |
| Dual ICIs therapy + CT | 2/266 | 1 | 0.75% |  |  |
| **Grade 3-5 diabetes** |  |  |  |  |  |
| Chemotherapy | 2/2770 | 11 | 0.39% | 0.05%–0.93% | 0.0% |
| ICI monotherapy | 3/2110 | 6 | 0.32% | 0.06%–0.71% | 0.0% |
| ICI monotherapy + CT | 14/2328 | 6 | 0.46% | 0.19%–0.74% | 0.0% |
| Dual ICIs therapy | 3/653 | 2 | 0.37% | 0.01%–0.83% | 0.0% |
| Dual ICIs therapy + CT | 2/266 | 1 | 0.75% |  |  |
| **Grade 1-5 pruritus** |  |  |  |  |  |
| Chemotherapy | 31/1662 | 8 | 1.97% | 1.25%–2.69% | 0.0% |
| ICI monotherapy | 69/901 | 5 | 6.96% | 3.63%–10.29% | 73.6% |
| ICI monotherapy + CT | 120/1370 | 4 | 7.18% | 1.66%–12.70% | 96.2% |
| Dual ICIs therapy | 16/115 | 1 | 13.91% |  |  |
| **Grade 3-5 pruritus** |  |  |  |  |  |
| Chemotherapy | 0/1662 | 8 | 0 |  |  |
| ICI monotherapy | 0/901 | 5 | 0 |  |  |
| ICI monotherapy + CT | 8/1370 | 4 | 0.65% | 0.18%–1.12% | 0.0% |
| Dual ICIs therapy | 1/115 | 1 | 0.87% |  |  |
| **Grade 1-5 rash** |  |  |  |  |  |
| Chemotherapy | 193/3665 | 14 | 5.59% | 3.54%–7.65% | 92.5% |
| ICI monotherapy | 125/1863 | 7 | 5.97% | 2.85%–9.09% | 93.7% |
| ICI monotherapy + CT | 466/2790 | 8 | 14.81% | 7.97%–21.64% | 97.3% |
| Dual ICIs therapy | 32/486 | 2 | 8.63% | 0.03%–17.99% | 87.5% |
| Dual ICIs therapy + CT | 19/266 | 1 | 7.14% |  |  |
| **Grade 3-5 rash** |  |  |  |  |  |
| Chemotherapy | 14/3730 | 15 | 0.84% | 0.24%–1.44% | 18.0% |
| ICI monotherapy | 13/1863 | 7 | 0.67% | 0.27%–1.06% | 0.0% |
| ICI monotherapy + CT | 59/2928 | 9 | 2.02% | 1.13%–2.92% | 66.2% |
| Dual ICIs therapy | 4/486 | 2 | 0.65% | 0.01%–1.36% | 0.0% |
| Dual ICIs therapy + CT | 6/266 | 1 | 2.26% |  |  |
| **Grade 1-5 severe skin reaction** | |  |  |  |  |
| Chemotherapy | 16/2649 | 10 | 0.46% | 0.19%–0.74% | 0.0% |
| ICI monotherapy | 38/1753 | 4 | 2.03% | 1.37%–2.69% | 0.0% |
| ICI monotherapy + CT | 28/2125 | 7 | 1.22% | 0.60%–1.84% | 34.8% |
| Dual ICIs therapy | 11/282 | 1 | 3.90% |  |  |
| **Grade 3-5 severe skin reaction** | |  |  |  |  |
| Chemotherapy | 9/2649 | 10 | 0.37% | 0.01%–0.75% | 19.0% |
| ICI monotherapy | 30/1753 | 4 | 1.57% | 0.98%–2.15% | 0.0% |
| ICI monotherapy + CT | 19/2125 | 7 | 0.94% | 0.23%–1.66% | 59.7% |
| Dual ICIs therapy | 10/282 | 1 | 3.55% |  |  |
| **Grade 1-5 myocarditis** |  |  |  |  |  |
| Chemotherapy | 2/1378 | 4 | 0.40% | 0.02%–0.95% | 0.0% |
| ICI monotherapy | 2/917 | 2 | 0.19% | 0.01%–0.48% | 0.0% |
| ICI monotherapy + CT | 1/779 | 3 | 0.34% | 0.03%–1.01% | 0.0% |
| Dual ICIs therapy | 3/282 | 1 | 1.06% |  |  |
| Dual ICIs therapy + CT | 1/266 | 1 | 0.38% |  |  |
| **Grade 3-5 myocarditis** |  |  |  |  |  |
| Chemotherapy | 1/1378 | 4 | 0.36% | 0.03%–1.07% | 0.0% |
| ICI monotherapy | 1/917 | 2 | 0.16% | 0.01%–0.47% | 0.0% |
| ICI monotherapy + CT | 1/779 | 3 | 0.34% | 0.03%–1.01% | 0.0% |
| Dual ICIs therapy | 2/282 | 1 | 0.71% |  |  |
| Dual ICIs therapy + CT | 1/266 | 1 | 0.38% |  |  |
| **Grade 1-5 nephritis** |  |  |  |  |  |
| Chemotherapy | 5/3139 | 12 | 0.51% | 0.01%–1.00% | 0.0% |
| ICI monotherapy | 11/1926 | 6 | 0.49% | 0.14%–0.84% | 0.0% |
| ICI monotherapy + CT | 18/2106 | 7 | 0.72% | 0.21%–1.24% | 43.8% |
| Dual ICIs therapy | 5/653 | 2 | 0.69% | 0.05%–1.32% | 0.0% |
| **Grade 3-5 nephritis** |  |  |  |  |  |
| Chemotherapy | 2/3139 | 12 | 0.71% | 0.07%–1.69% | 0.0% |
| ICI monotherapy | 5/1926 | 6 | 0.24% | 0.00%–0.49% | 0.0% |
| ICI monotherapy + CT | 14/2106 | 7 | 0.80% | 0.33%–1.26% | 0.0% |
| Dual ICIs therapy | 1/653 | 2 | 0.35% | 0.03%–1.04% | 0.0% |
| **Grade 1-5 hepatitis** |  |  |  |  |  |
| Chemotherapy | 22/4141 | 17 | 1.12% | 0.25%–2.00% | 58.2% |
| ICI monotherapy | 25/2978 | 9 | 0.55% | 0.24%–0.86% | 22.9% |
| ICI monotherapy + CT | 74/2928 | 10 | 2.30% | 1.40%–3.19% | 61.0% |
| Dual ICIs therapy | 18/826 | 3 | 2.14% | 1.15%–3.12% | 0.0% |
| Dual ICIs therapy + CT | 11/266 | 1 | 4.14% |  |  |
| **Grade 3-5 hepatitis** |  |  |  |  |  |
| Chemotherapy | 2/4141 | 17 | 0.34% | 0.04%–0.82% | 0.0% |
| ICI monotherapy | 15/2978 | 9 | 0.41% | 0.09%–0.72% | 32.8% |
| ICI monotherapy + CT | 35/2928 | 10 | 0.99% | 0.54%–1.44% | 28.7% |
| Dual ICIs therapy | 15/826 | 3 | 1.76% | 0.86%–2.65% | 0.0% |
| Dual ICIs therapy + CT | 8/266 | 1 | 3.01% |  |  |
| **Grade 1-5 myositis** |  |  |  |  |  |
| Chemotherapy | 2/1222 | 5 | 0.31% | 0.02%–0.74% | 0.0% |
| ICI monotherapy | 9/1294 | 4 | 0.55% | 0.15%–0.96% | 0.0% |
| ICI monotherapy + CT | 4/1299 | 3 | 0.29% | 0.00%–0.59% | 0.0% |
| Dual ICIs therapy | 4/455 | 2 | 1.42% | 0.04%–2.80% | 0.0% |
| **Grade 3-5 myositis** |  |  |  |  |  |
| Chemotherapy | 0/1222 | 5 | 0 |  |  |
| ICI monotherapy | 0/1294 | 4 | 0 |  |  |
| ICI monotherapy + CT | 1/1299 | 3 | 0.45% | 0.03%–1.33% | 0.0% |
| Dual ICIs therapy | 3/455 | 2 | 1.06% | 0.04%–2.26% | 0.0% |
| **Grade 1-5 hypersensitivity/infusion reaction** | | |  |  |  |
| Chemotherapy | 114/4536 | 17 | 2.44% | 1.68%–3.20% | 61.6% |
| ICI monotherapy | 167/4086 | 13 | 3.43% | 2.08%–4.78% | 89.7% |
| ICI monotherapy + CT | 54/2290 | 7 | 2.24% | 1.30%–3.18% | 55.8% |
| Dual ICIs therapy | 54/1124 | 4 | 4.47% | 2.99%–5.94% | 33.2% |
| Dual ICIs therapy + CT | 18/358 | 1 | 5.03% |  |  |
| **Grade 3-5 hypersensitivity/infusion reaction** | | |  |  |  |
| Chemotherapy | 21/4601 | 18 | 0.49% | 0.26%–0.72% | 0.0% |
| ICI monotherapy | 14/4086 | 13 | 0.32% | 0.11%–0.54% | 0.0% |
| ICI monotherapy + CT | 15/2428 | 8 | 0.40% | 0.04%–0.76% | 36.6% |
| Dual ICIs therapy | 7/1124 | 4 | 0.67% | 0.02%–1.36% | 25.0% |
| Dual ICIs therapy + CT | 2/358 | 1 | 0.56% |  |  |

AE: adverse event; irAE: immune-related adverse event; ICI: immune checkpoint inhibitor; CT: chemotherapy; No.: number; 95% CI: 95% confidence interval.

# **Table S12. Ranking of treatments for AE and irAE based on cumulative probability**

| Ranking | Chemotherapy | ICI monotherapy | ICI monotherapy + CT | Dual ICIs | Dual ICIs +CT |
| --- | --- | --- | --- | --- | --- |
| Grade 1-5 AE | |  |  |  |  |
| First | 0.0% | 0.0% | 43.8% | 0.0% | **56.2%** |
| Second | 8.1% | 0.0% | **56.0%** | 0.0% | 35.9% |
| Third | **91.9%** | 0.0% | 0.2% | 0.0% | 7.9% |
| Fourth | 0.0% | 0.0% | 0.0% | **100.0%** | 0.0% |
| Fifth | 0.0% | **100.0%** | 0.0% | 0.0% | 0.0% |
| Grade 3-5 AE | |  |  |  |  |
| First | 0.1% | 0.0% | 31.4% | 0.0% | **68.5%** |
| Second | 8.2% | 0.0% | **67.9%** | 0.0% | 23.8% |
| Third | **91.1%** | 0.0% | 0.6% | 1.1% | 7.3% |
| Fourth | 0.6% | 0.0% | 0.0% | **98.9%** | 0.4% |
| Fifth | 0.0% | **100.0%** | 0.0% | 0.0% | 0.0% |
| Grade 1-5 irAE | |  |  |  |  |
| First | 0.0% | 1.2% | 0.0% | 48.3% | **50.5%** |
| Second | 0.0% | 17.5% | 0.1% | **47.2%** | 35.3% |
| Third | 0.0% | **80.0%** | 1.9% | 4.3% | 13.7% |
| Fourth | 0.0% | 1.4% | **98.0%** | 0.1% | 0.5% |
| Fifth | **100.0%** | 0.0% | 0.0% | 0.0% | 0.0% |
| Grade 3-5 irAE | |  |  |  |  |
| First | 0.0% | 0.5% | 0.0% | **60.4%** | 39.1% |
| Second | 0.0% | 19.3% | 0.1% | 38.1% | **42.5%** |
| Third | 0.0% | **76.3%** | 4.9% | 1.3% | 17.4% |
| Fourth | 0.0% | 3.9% | **95.0%** | 0.1% | 1.0% |
| Fifth | **100.0%** | 0.0% | 0.0% | 0.0% | 0.0% |

AE: adverse event; irAE: immune-related adverse event; ICI: immune checkpoint inhibitor; CT: chemotherapy.

# **Table S13. Evaluation of inconsistency for the primary outcomes among different treatment regimen**

| **Treatment comparison** | **Consistency model** | **Inconsistency model** | **P for interaction** |
| --- | --- | --- | --- |
| **Grade 1-5 AE** |  |  |  |
| ICI monotherapy vs. Chemotherapy | 0.27 (0.22, 0.33) | 1.55 (0.74, 3.22) | **< 0.01** |
| ICI monotherapy + CT vs. Chemotherapy | 1.42 (1.11, 1.82) | 0.90 (0.42, 1.91) | 0.262 |
| Dual ICIs therapy vs. Chemotherapy | 0.49 (0.36, 0.68) | 0.27 (0.22, 0.34) | **0.002** |
| Dual ICIs therapy + CT vs. Chemotherapy | 1.49 (0.86, 2.57) | 0.52 (0.21, 1.30) | 0.052 |
| ICI monotherapy + CT vs. ICI monotherapy | 5.26 (3.88, 7.13) | 9.98 (4.04, 24.61) | 0.188 |
| Dual ICIs therapy vs. ICI monotherapy | 1.82 (1.35, 2.45) | 2.69 (1.30, 5.58) | 0.331 |
| Dual ICIs therapy + CT vs. ICI monotherapy | 5.49 (3.07, 9.82) | 3.86 (1.60, 9.28) | 0.512 |
| Dual ICIs therapy vs. ICI monotherapy + CT | 0.35 (0.23, 0.51) | 0.27 (0.11, 0.64) | 0.599 |
| Dual ICIs therapy + CT vs. ICI monotherapy + CT | 1.04 (0.59, 1.85) | 0.39 (0.11, 1.31) | 0.159 |
| Dual ICIs therapy + CT vs. Dual ICIs therapy | 3.01 (1.59, 5.69) | 1.43 (0.48, 4.31) | 0.250 |
| **Grade 3-5 AE** |  |  |  |
| ICI monotherapy vs. Chemotherapy | 0.20 (0.16, 0.26) | 2.35 (0.84, 6.55) | **< 0.01** |
| ICI monotherapy + CT vs. Chemotherapy | 1.36 (1.06, 1.74) | 1.88 (0.66, 5.33) | 0.554 |
| Dual ICIs therapy vs. Chemotherapy | 0.56 (0.36, 0.89) | 0.18 (0.14, 0.24) | **< 0.01** |
| Dual ICIs therapy + CT vs. Chemotherapy | 1.60 (0.84, 3.04) | 0.62 (0.22, 1.75) | 0.128 |
| ICI monotherapy + CT vs. ICI monotherapy | 6.80 (4.81, 9.61) | 12.30 (4.35, 34.77) | 0.299 |
| Dual ICIs therapy vs. ICI monotherapy | 2.82 (1.83, 4.33) | 4.76 (1.73, 13.05) | 0.350 |
| Dual ICIs therapy + CT vs. ICI monotherapy | 7.99 (4.01, 15.91) | 7.32 (2.59, 20.66) | 0.890 |
| Dual ICIs therapy vs. ICI monotherapy + CT | 0.41 (0.25, 0.69) | 0.39 (0.14, 1.05) | 0.931 |
| Dual ICIs therapy + CT vs. ICI monotherapy + CT | 1.18 (0.61, 2.28) | 0.59 (0.14, 2.44) | 0.388 |
| Dual ICIs therapy + CT vs. Dual ICIs therapy | 2.84 (1.29, 6.22) | 1.54 (0.38, 6.17) | 0.454 |
| **Grade 1-5 irAE** |  |  |  |
| ICI monotherapy vs. Chemotherapy | 5.50 (3.39, 8.93) | 0.81 (0.26, 2.54) | **< 0.01** |
| ICI monotherapy + CT vs. Chemotherapy | 2.85 (2.08, 3.90) | 5.45 (3.33, 8.93) | **0.030** |
| Dual ICIs therapy vs. Chemotherapy | 9.52 (4.46, 20.34) | 3.56 (1.11, 11.44) | 0.166 |
| Dual ICIs therapy + CT vs. Chemotherapy | 9.62 (3.77, 24.59) | 2.60 (1.95, 3.47) | **< 0.01** |
| ICI monotherapy + CT vs. ICI monotherapy | 0.52 (0.29, 0.92) | 0.48 (0.27, 0.84) | 0.846 |
| Dual ICIs therapy vs. ICI monotherapy | 1.73 (0.88, 3.39) | 2.05 (0.67, 6.28) | 0.799 |
| Dual ICIs therapy + CT vs. ICI monotherapy | 1.75 (0.61, 5.00) | 3.83 (1.13, 13.02) | 0.341 |
| Dual ICIs therapy vs. ICI monotherapy + CT | 3.34 (1.47, 7.58) | 4.30 (1.51, 12.23) | 0.709 |
| Dual ICIs therapy + CT vs. ICI monotherapy + CT | 3.37 (1.35, 8.41) | 8.03 (2.53, 25.52) | 0.248 |
| Dual ICIs therapy + CT vs. Dual ICIs therapy | 1.01 (0.30, 3.37) | 1.87 (0.41, 8.41) | 0.533 |
| **Grade 3-5 irAE** |  |  |  |
| ICI monotherapy vs. Chemotherapy | 6.65 (3.30, 13.39) | 0.76 (0.13, 4.66) | **0.027** |
| ICI monotherapy + CT vs. Chemotherapy | 3.27 (2.20, 4.85) | 6.90 (3.01, 15.78) | 0.111 |
| Dual ICIs therapy vs. Chemotherapy | 15.14 (5.82, 39.40) | 4.40 (0.43, 44.84) | 0.335 |
| Dual ICIs therapy + CT vs. Chemotherapy | 12.26 (3.89, 38.64) | 3.11 (2.03, 4.74) | 0.028 |
| ICI monotherapy + CT vs. ICI monotherapy | 0.49 (0.22, 1.10) | 0.45 (0.18, 1.15) | 0.892 |
| Dual ICIs therapy vs. ICI monotherapy | 2.28 (1.06, 4.88) | 2.27 (0.39, 13.26) | 0.996 |
| Dual ICIs therapy + CT vs. ICI monotherapy | 1.84 (0.48, 7.05) | 6.01 (0.55, 65.54) | 0.397 |
| Dual ICIs therapy vs. ICI monotherapy + CT | 4.63 (1.64, 13.04) | 5.04 (1.00, 25.36) | 0.931 |
| Dual ICIs therapy + CT vs. ICI monotherapy + CT | 3.75 (1.25, 11.28) | 13.36 (1.37, 130.62) | 0.325 |
| Dual ICIs therapy + CT vs. Dual ICIs therapy | 0.81 (0.18, 3.60) | 2.65 (0.17, 40.62) | 0.457 |

AE: adverse event; irAE: immune-related adverse event; ICI: immune checkpoint inhibitor; CT: chemotherapy.

# **Table S14. Node**–**splitting analysis of network meta-analysis**

|  | **Direct effect** | | **Indirect effect** | |  |
| --- | --- | --- | --- | --- | --- |
| **Nodes** | **coefficient** | **SE** | **coefficient** | **SE** | **P** |
| **Grade 1-5 AE** |  |  |  |  |  |
| Chemotherapy, ICI monotherapy | -1.28 | 0.10 | -1.90 | 0.46 | 0.18 |
| Chemotherapy, ICI monotherapy + CT | 0.36 | 0.13 | 0.12 | 0.54 | 0.65 |
| Chemotherapy, Dual ICIs therapy | -0.74 | 0.26 | -0.68 | 0.23 | 0.88 |
| Chemotherapy, Dual ICIs therapy + CT | 0.32 | 0.30 | 0.89 | 0.79 | 0.50 |
| ICI monotherapy, ICI monotherapy + CT | 1.72 | 0.43 | 1.65 | 0.17 | 0.89 |
| ICI monotherapy, Dual ICIs therapy | 0.60 | 0.16 | 0.60 | 0.48 | 1.00 |
| ICI monotherapy + CT, Dual ICIs therapy | -1.18 | 0.43 | -1.02 | 0.24 | 0.75 |
| ICI monotherapy + CT, Dual ICIs therapy + CT | 0.09 | 0.43 | 0.002 | 0.40 | 0.88 |
| **Grade 3-5 AE** |  |  |  |  |  |
| Chemotherapy, ICI monotherapy | -1.59 | 0.13 | -1.89 | 0.58 | 0.62 |
| Chemotherapy, ICI monotherapy + CT | 0.33 | 0.13 | -0.43 | 0.67 | 0.26 |
| Chemotherapy, Dual ICIs therapy | -0.33 | 0.35 | -0.77 | 0.32 | 0.36 |
| Chemotherapy, Dual ICIs therapy + CT | 0.34 | 0.35 | 1.36 | 0.90 | 0.29 |
| ICI monotherapy, ICI monotherapy + CT | 1.64 | 0.52 | 1.95 | 0.19 | 0.57 |
| ICI monotherapy, Dual ICIs therapy | 0.91 | 0.23 | 1.88 | 0.61 | 0.14 |
| ICI monotherapy + CT, Dual ICIs therapy | -0.94 | 0.51 | -0.86 | 0.31 | 0.89 |
| ICI monotherapy + CT, Dual ICIs therapy + CT | 0.48 | 0.50 | -0.11 | 0.46 | 0.39 |
| **Grade 1-5 irAE** |  |  |  |  |  |
| Chemotherapy, ICI monotherapy | 1.67 | 0.25 | 3.04 | 1.36 | 0.32 |
| Chemotherapy, Dual ICIs therapy | 2.47 | 0.57 | 2.04 | 0.57 | 0.60 |
| Chemotherapy, Dual ICIs therapy + CT | 3.04 | 0.56 | 0.50 | 0.90 | **0.03** |
| ICI monotherapy, Dual ICIs therapy | 0.55 | 0.38 | 0.49 | 1.20 | 0.96 |
| ICI monotherapy + CT, Dual ICIs therapy + CT | 0.81 | 0.44 | 3.36 | 1.08 | **0.03** |
| **Grade 3-5 irAE** |  |  |  |  |  |
| Chemotherapy, ICI monotherapy | 1.87 | 0.37 | 2.77 | 1.60 | 0.58 |
| Chemotherapy, Dual ICIs therapy | 2.88 | 0.75 | 2.58 | 0.71 | 0.78 |
| Chemotherapy, Dual ICIs therapy + CT | 3.73 | 1.12 | 0.77 | 1.48 | 0.20 |
| ICI monotherapy, Dual ICIs therapy | 0.84 | 0.42 | 0.59 | 1.69 | 0.89 |
| ICI monotherapy + CT, Dual ICIs therapy + CT | 1.11 | 0.58 | 4.06 | 2.23 | 0.20 |

AE: adverse event; irAE: immune-related adverse event; ICI: immune checkpoint inhibitor; CT: chemotherapy.

# **Table S15. Sensitivity analysis in network meta-analysis**

|  | **Rank of risk** | | | | |
| --- | --- | --- | --- | --- | --- |
| **Groups** | **1st** | **2nd** | **3rd** | **4th** | **5th** |
| **Phase III studies** |  |  |  |  |  |
| **Grade 1-5 AE** |  |  |  |  |  |
| Chemotherapy | 0.0 | 6.9 | 93.1 | 0.0 | 0.0 |
| ICI monotherapy | 0.0 | 0.0 | 0.0 | 0.1 | 100.0 |
| ICI monotherapy + CT | 49.3 | 50.6 | 0.1 | 0.0 | 0.0 |
| Dual ICIs therapy | 0.0 | 0.0 | 0.0 | 100.0 | 0.1 |
| Dual ICIs therapy + CT | 50.7 | 42.4 | 6.9 | 0.0 | 0.0 |
| **Grade 3-5 AE** |  |  |  |  |  |
| Chemotherapy | 0.3 | 10.1 | 88.2 | 1.4 | 0.0 |
| ICI monotherapy | 0.0 | 0.0 | 0.0 | 0.0 | 100.0 |
| ICI monotherapy + CT | 30.2 | 68.2 | 1.6 | 0.0 | 0.0 |
| Dual ICIs therapy | 0.0 | 0.3 | 2.0 | 97.8 | 0.0 |
| Dual ICIs therapy + CT | 69.6 | 21.5 | 8.2 | 0.8 | 0.0 |
| **Grade 1-5 irAE** |  |  |  |  |  |
| Chemotherapy | 0.0 | 0.0 | 0.0 | 0.0 | 100.0 |
| ICI monotherapy | 0.7 | 17.3 | 81.8 | 0.2 | 0.0 |
| ICI monotherapy + CT | 0.0 | 0.0 | 0.4 | 99.6 | 0.0 |
| Dual ICIs therapy | 55.6 | 42.0 | 0.4 | 0.0 | 0.0 |
| Dual ICIs therapy + CT | 43.7 | 40.8 | 15.4 | 0.1 | 0.0 |
| **Grade 3-5 irAE** |  |  |  |  |  |
| Chemotherapy | 0.0 | 0.0 | 0.0 | 0.0 | 100.0 |
| ICI monotherapy | 0.7 | 19.9 | 73.3 | 6.1 | 0.0 |
| ICI monotherapy + CT | 0.0 | 0.4 | 7.1 | 92.5 | 0.0 |
| Dual ICIs therapy | 57.6 | 40.2 | 2.1 | 0.2 | 0.0 |
| Dual ICIs therapy + CT | 41.7 | 39.5 | 17.5 | 1.3 | 0.0 |
| **NSCLC patients** |  |  |  |  |  |
| **Grade 1-5 AE** |  |  |  |  |  |
| Chemotherapy | 0.0 | 6.0 | 94.0 | 0.0 | 0.0 |
| ICI monotherapy | 0.0 | 0.0 | 0.0 | 0.9 | 99.1 |
| ICI monotherapy + CT | 33.7 | 66.2 | 0.1 | 0.0 | 0.0 |
| Dual ICIs therapy | 0.0 | 0.0 | 0.0 | 99.1 | 0.9 |
| Dual ICIs therapy + CT | 66.3 | 27.8 | 5.9 | 0.0 | 0.0 |
| **Grade 3-5 AE** |  |  |  |  |  |
| Chemotherapy | 0.0 | 17.7 | 82.2 | 0.1 | 0.0 |
| ICI monotherapy | 0.0 | 0.0 | 0.0 | 0.1 | 99.9 |
| ICI monotherapy + CT | 52.2 | 47.8 | 0.0 | 0.0 | 0.0 |
| Dual ICIs therapy | 0.0 | 0.0 | 1.2 | 98.7 | 0.1 |
| Dual ICIs therapy + CT | 47.8 | 34.5 | 16.6 | 1.1 | 0.0 |
| **Grade 1-5 irAE** |  |  |  |  |  |
| Chemotherapy | 0.0 | 0.0 | 0.0 | 100.0 | - |
| ICI monotherapy | 5.0 | 94.0 | 1.0 | 0.0 | - |
| ICI monotherapy + CT | 0.1 | 1.1 | 98.9 | 0.0 | - |
| Dual ICIs therapy | 94.9 | 4.9 | 0.2 | 0.0 | - |
| **Grade 3-5 irAE** |  |  |  |  |  |
| Chemotherapy | 0.0 | 0.0 | 0.0 | 100.0 | - |
| ICI monotherapy | 0.2 | 98.8 | 1.0 | 0.0 | - |
| ICI monotherapy + CT | 0.0 | 1.0 | 99.0 | 0.0 | - |
| Dual ICIs therapy | 99.8 | 0.2 | 0.0 | 0.0 | - |

AE: adverse event; irAE: immune-related adverse event; ICI: immune checkpoint inhibitor; CT: chemotherapy.

# **Figure S1. Publication bias for treatments**


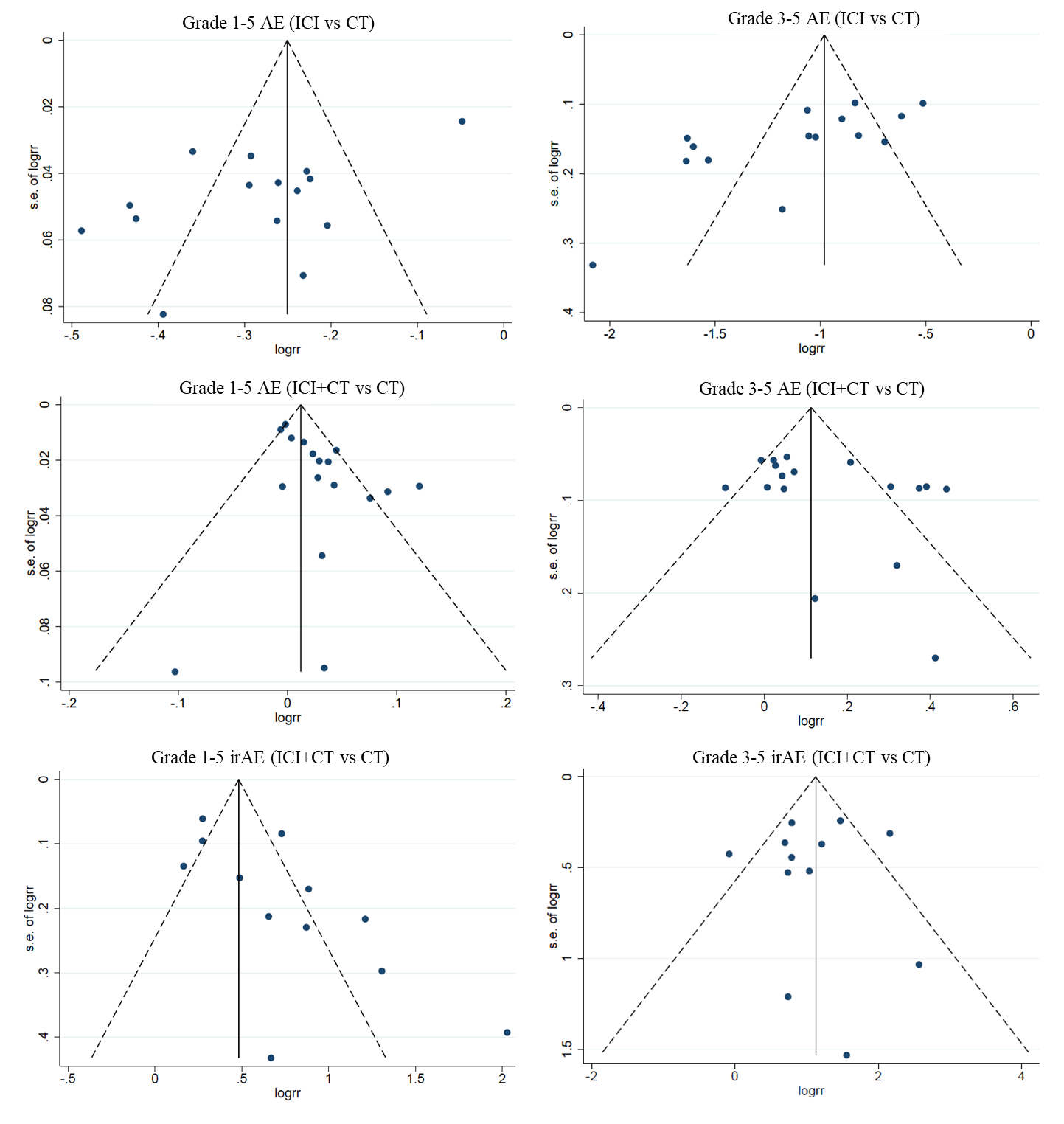


AE: adverse event; irAE: immune-related adverse event; ICI: immune checkpoint inhibitor; CT: chemotherapy.

# **References**

Afzal, M.Z., Dragnev, K., and Shirai, K. (2018). A tertiary care cancer center experience with carboplatin and pemetrexed in combination with pembrolizumab in comparison with carboplatin and pemetrexed alone in non-squamous non-small cell lung cancer. *J Thorac Dis* 10(6)**,** 3575-3584. doi: 10.21037/jtd.2018.06.08.

Antonia, S.J., López-Martin, J.A., Bendell, J., Ott, P.A., Taylor, M., Eder, J.P., et al. (2016). Nivolumab alone and nivolumab plus ipilimumab in recurrent small-cell lung cancer (CheckMate 032): a multicentre, open-label, phase 1/2 trial. *Lancet Oncol* 17(7)**,** 883-895. doi: 10.1016/s1470-2045(16)30098-5.

Antonia, S.J., Villegas, A., Daniel, D., Vicente, D., Murakami, S., Hui, R., et al. (2018). Overall Survival with Durvalumab after Chemoradiotherapy in Stage III NSCLC. *N Engl J Med* 379(24)**,** 2342-2350. doi: 10.1056/NEJMoa1809697.

Antonia, S.J., Villegas, A., Daniel, D., Vicente, D., Murakami, S., Hui, R., et al. (2017). Durvalumab after Chemoradiotherapy in Stage III Non-Small-Cell Lung Cancer. *N Engl J Med* 377(20)**,** 1919-1929. doi: 10.1056/NEJMoa1709937.

Arrieta, O., Barrón, F., Ramírez-Tirado, L.A., Zatarain-Barrón, Z.L., Cardona, A.F., Díaz-García, D., et al. (2020). Efficacy and Safety of Pembrolizumab Plus Docetaxel vs Docetaxel Alone in Patients With Previously Treated Advanced Non-Small Cell Lung Cancer: The PROLUNG Phase 2 Randomized Clinical Trial. *JAMA Oncol* 6(6)**,** 856-864. doi: 10.1001/jamaoncol.2020.0409.

Awad, M.M., Gadgeel, S.M., Borghaei, H., Patnaik, A., Yang, J.C., Powell, S.F., et al. (2021). Long-Term Overall Survival From KEYNOTE-021 Cohort G: Pemetrexed and Carboplatin With or Without Pembrolizumab as First-Line Therapy for Advanced Nonsquamous NSCLC. *J Thorac Oncol* 16(1)**,** 162-168. doi: 10.1016/j.jtho.2020.09.015.

Barlesi, F., Vansteenkiste, J., Spigel, D., Ishii, H., Garassino, M., de Marinis, F., et al. (2018). Avelumab versus docetaxel in patients with platinum-treated advanced non-small-cell lung cancer (JAVELIN Lung 200): an open-label, randomised, phase 3 study. *Lancet Oncol* 19(11)**,** 1468-1479. doi: 10.1016/s1470-2045(18)30673-9.

Bordoni, R., Ciardiello, F., von Pawel, J., Cortinovis, D., Karagiannis, T., Ballinger, M., et al. (2018). Patient-Reported Outcomes in OAK: A Phase III Study of Atezolizumab Versus Docetaxel in Advanced Non-Small-cell Lung Cancer. *Clin Lung Cancer* 19(5)**,** 441-449.e444. doi: 10.1016/j.cllc.2018.05.011.

Borghaei, H., Langer, C.J., Gadgeel, S., Papadimitrakopoulou, V.A., Patnaik, A., Powell, S.F., et al. (2019). 24-Month Overall Survival from KEYNOTE-021 Cohort G: Pemetrexed and Carboplatin with or without Pembrolizumab as First-Line Therapy for Advanced Nonsquamous Non-Small Cell Lung Cancer. *J Thorac Oncol* 14(1)**,** 124-129. doi: 10.1016/j.jtho.2018.08.004.

Borghaei, H., Paz-Ares, L., Horn, L., Spigel, D.R., Steins, M., Ready, N.E., et al. (2015). Nivolumab versus Docetaxel in Advanced Nonsquamous Non-Small-Cell Lung Cancer. *N Engl J Med* 373(17)**,** 1627-1639. doi: 10.1056/NEJMoa1507643.

Borghaei, H., Redman, M.W., Kelly, K., Waqar, S.N., Robert, F., Kiefer, G.J., et al. (2020). SWOG S1400A (NCT02154490): A Phase II Study of Durvalumab for Patients With Previously Treated Stage IV or Recurrent Squamous Cell Lung Cancer (Lung-MAP Sub-study). *Clin Lung Cancer*. doi: 10.1016/j.cllc.2020.10.015.

Boyer, M., Şendur, M.A.N., Rodríguez-Abreu, D., Park, K., Lee, D.H., Çiçin, I., et al. (2021). Pembrolizumab Plus Ipilimumab or Placebo for Metastatic Non-Small-Cell Lung Cancer With PD-L1 Tumor Proportion Score ≥ 50%: Randomized, Double-Blind Phase III KEYNOTE-598 Study. *J Clin Oncol***,** Jco2003579. doi: 10.1200/jco.20.03579.

Brahmer, J., Reckamp, K.L., Baas, P., Crinò, L., Eberhardt, W.E., Poddubskaya, E., et al. (2015). Nivolumab versus Docetaxel in Advanced Squamous-Cell Non-Small-Cell Lung Cancer. *N Engl J Med* 373(2)**,** 123-135. doi: 10.1056/NEJMoa1504627.

Brahmer, J.R., Rodríguez-Abreu, D., Robinson, A.G., Hui, R., Csőszi, T., Fülöp, A., et al. (2017). Health-related quality-of-life results for pembrolizumab versus chemotherapy in advanced, PD-L1-positive NSCLC (KEYNOTE-024): a multicentre, international, randomised, open-label phase 3 trial. *Lancet Oncol* 18(12)**,** 1600-1609. doi: 10.1016/s1470-2045(17)30690-3.

Carbone, D.P., Reck, M., Paz-Ares, L., Creelan, B., Horn, L., Steins, M., et al. (2017). First-Line Nivolumab in Stage IV or Recurrent Non-Small-Cell Lung Cancer. *N Engl J Med* 376(25)**,** 2415-2426. doi: 10.1056/NEJMoa1613493.

Chalabi, M., Cardona, A., Nagarkar, D.R., Dhawahir Scala, A., Gandara, D.R., Rittmeyer, A., et al. (2020). Efficacy of chemotherapy and atezolizumab in patients with non-small-cell lung cancer receiving antibiotics and proton pump inhibitors: pooled post hoc analyses of the OAK and POPLAR trials. *Ann Oncol* 31(4)**,** 525-531. doi: 10.1016/j.annonc.2020.01.006.

Faivre-Finn, C., Spigel, D.R., Senan, S., Langer, C., Perez, B.A., Özgüroğlu, M., et al. (2020). Impact of prior chemoradiotherapy-related variables on outcomes with durvalumab in unresectable Stage III NSCLC (PACIFIC). *Lung Cancer* 151**,** 30-38. doi: 10.1016/j.lungcan.2020.11.024.

Fehrenbacher, L., Spira, A., Ballinger, M., Kowanetz, M., Vansteenkiste, J., Mazieres, J., et al. (2016). Atezolizumab versus docetaxel for patients with previously treated non-small-cell lung cancer (POPLAR): a multicentre, open-label, phase 2 randomised controlled trial. *Lancet* 387(10030)**,** 1837-1846. doi: 10.1016/s0140-6736(16)00587-0.

Fehrenbacher, L., von Pawel, J., Park, K., Rittmeyer, A., Gandara, D.R., Ponce Aix, S., et al. (2018). Updated Efficacy Analysis Including Secondary Population Results for OAK: A Randomized Phase III Study of Atezolizumab versus Docetaxel in Patients with Previously Treated Advanced Non-Small Cell Lung Cancer. *J Thorac Oncol* 13(8)**,** 1156-1170. doi: 10.1016/j.jtho.2018.04.039.

Gadgeel, S., Rodríguez-Abreu, D., Speranza, G., Esteban, E., Felip, E., Dómine, M., et al. (2020). Updated Analysis From KEYNOTE-189: Pembrolizumab or Placebo Plus Pemetrexed and Platinum for Previously Untreated Metastatic Nonsquamous Non-Small-Cell Lung Cancer. *J Clin Oncol* 38(14)**,** 1505-1517. doi: 10.1200/jco.19.03136.

Gadgeel, S.M., Lukas, R.V., Goldschmidt, J., Conkling, P., Park, K., Cortinovis, D., et al. (2019). Atezolizumab in patients with advanced non-small cell lung cancer and history of asymptomatic, treated brain metastases: Exploratory analyses of the phase III OAK study. *Lung Cancer* 128**,** 105-112. doi: 10.1016/j.lungcan.2018.12.017.

Gadgeel, S.M., Stevenson, J.P., Langer, C.J., Gandhi, L., Borghaei, H., Patnaik, A., et al. (2018). Pembrolizumab and platinum-based chemotherapy as first-line therapy for advanced non-small-cell lung cancer: Phase 1 cohorts from the KEYNOTE-021 study. *Lung Cancer* 125**,** 273-281. doi: 10.1016/j.lungcan.2018.08.019.

Gandhi, L., Rodríguez-Abreu, D., Gadgeel, S., Esteban, E., Felip, E., De Angelis, F., et al. (2018). Pembrolizumab plus Chemotherapy in Metastatic Non-Small-Cell Lung Cancer. *N Engl J Med* 378(22)**,** 2078-2092. doi: 10.1056/NEJMoa1801005.

Garassino, M.C., Gadgeel, S., Esteban, E., Felip, E., Speranza, G., Domine, M., et al. (2020). Patient-reported outcomes following pembrolizumab or placebo plus pemetrexed and platinum in patients with previously untreated, metastatic, non-squamous non-small-cell lung cancer (KEYNOTE-189): a multicentre, double-blind, randomised, placebo-controlled, phase 3 trial. *Lancet Oncol* 21(3)**,** 387-397. doi: 10.1016/s1470-2045(19)30801-0.

Garon, E.B., Kim, J.S., and Govindan, R. (2020). Pemetrexed maintenance with or without pembrolizumab in non-squamous non-small cell lung cancer: A cross-trial comparison of KEYNOTE-189 versus PARAMOUNT, PRONOUNCE, and JVBL. *Lung Cancer* 151**,** 25-29. doi: 10.1016/j.lungcan.2020.11.018.

Goldman, J.W., Dvorkin, M., Chen, Y., Reinmuth, N., Hotta, K., Trukhin, D., et al. (2021). Durvalumab, with or without tremelimumab, plus platinum-etoposide versus platinum-etoposide alone in first-line treatment of extensive-stage small-cell lung cancer (CASPIAN): updated results from a randomised, controlled, open-label, phase 3 trial. *Lancet Oncol* 22(1)**,** 51-65. doi: 10.1016/s1470-2045(20)30539-8.

Goldman, J.W., Garassino, M.C., Chen, Y., Özgüroğlu, M., Dvorkin, M., Trukhin, D., et al. (2020). Patient-reported outcomes with first-line durvalumab plus platinum-etoposide versus platinum-etoposide in extensive-stage small-cell lung cancer (CASPIAN): a randomized, controlled, open-label, phase III study. *Lung Cancer* 149**,** 46-52. doi: 10.1016/j.lungcan.2020.09.003.

Govindan, R., Szczesna, A., Ahn, M.J., Schneider, C.P., Gonzalez Mella, P.F., Barlesi, F., et al. (2017). Phase III Trial of Ipilimumab Combined With Paclitaxel and Carboplatin in Advanced Squamous Non-Small-Cell Lung Cancer. *J Clin Oncol* 35(30)**,** 3449-3457. doi: 10.1200/jco.2016.71.7629.

Halmos, B., Burke, T., Kalyvas, C., Insinga, R., Vandormael, K., Frederickson, A., et al. (2020). A Matching-Adjusted Indirect Comparison of Pembrolizumab + Chemotherapy vs. Nivolumab + Ipilimumab as First-Line Therapies in Patients with PD-L1 TPS ≥1% Metastatic NSCLC. *Cancers (Basel)* 12(12). doi: 10.3390/cancers12123648.

Hellmann, M.D., Callahan, M.K., Awad, M.M., Calvo, E., Ascierto, P.A., Atmaca, A., et al. (2018a). Tumor Mutational Burden and Efficacy of Nivolumab Monotherapy and in Combination with Ipilimumab in Small-Cell Lung Cancer. *Cancer Cell* 33(5)**,** 853-861.e854. doi: 10.1016/j.ccell.2018.04.001.

Hellmann, M.D., Ciuleanu, T.E., Pluzanski, A., Lee, J.S., Otterson, G.A., Audigier-Valette, C., et al. (2018b). Nivolumab plus Ipilimumab in Lung Cancer with a High Tumor Mutational Burden. *N Engl J Med* 378(22)**,** 2093-2104. doi: 10.1056/NEJMoa1801946.

Hellmann, M.D., Paz-Ares, L., Bernabe Caro, R., Zurawski, B., Kim, S.W., Carcereny Costa, E., et al. (2019). Nivolumab plus Ipilimumab in Advanced Non-Small-Cell Lung Cancer. *N Engl J Med* 381(21)**,** 2020-2031. doi: 10.1056/NEJMoa1910231.

Herbst, R.S., Baas, P., Kim, D.W., Felip, E., Pérez-Gracia, J.L., Han, J.Y., et al. (2016). Pembrolizumab versus docetaxel for previously treated, PD-L1-positive, advanced non-small-cell lung cancer (KEYNOTE-010): a randomised controlled trial. *Lancet* 387(10027)**,** 1540-1550. doi: 10.1016/s0140-6736(15)01281-7.

Herbst, R.S., Garon, E.B., Kim, D.W., Cho, B.C., Perez-Gracia, J.L., Han, J.Y., et al. (2020a). Long-Term Outcomes and Retreatment Among Patients With Previously Treated, Programmed Death-Ligand 1â€’Positive, Advanced Nonâ€’Small-Cell Lung Cancer in the KEYNOTE-010 Study. *Journal of clinical oncology***,** JCO1902446‐. doi: 10.1200/JCO.19.02446.

Herbst, R.S., Giaccone, G., de Marinis, F., Reinmuth, N., Vergnenegre, A., Barrios, C.H., et al. (2020b). Atezolizumab for First-Line Treatment of PD-L1-Selected Patients with NSCLC. *N Engl J Med* 383(14)**,** 1328-1339. doi: 10.1056/NEJMoa1917346.

Hida, T., Kaji, R., Satouchi, M., Ikeda, N., Horiike, A., Nokihara, H., et al. (2018). Atezolizumab in Japanese Patients With Previously Treated Advanced Non-Small-Cell Lung Cancer: A Subgroup Analysis of the Phase 3 OAK Study. *Clin Lung Cancer* 19(4)**,** e405-e415. doi: 10.1016/j.cllc.2018.01.004.

Horn, L., Mansfield, A.S., Szczęsna, A., Havel, L., Krzakowski, M., Hochmair, M.J., et al. (2018). First-Line Atezolizumab plus Chemotherapy in Extensive-Stage Small-Cell Lung Cancer. *N Engl J Med* 379(23)**,** 2220-2229. doi: 10.1056/NEJMoa1809064.

Horn, L., Spigel, D.R., Vokes, E.E., Holgado, E., Ready, N., Steins, M., et al. (2017). Nivolumab Versus Docetaxel in Previously Treated Patients With Advanced Non-Small-Cell Lung Cancer: Two-Year Outcomes From Two Randomized, Open-Label, Phase III Trials (CheckMate 017 and CheckMate 057). *J Clin Oncol* 35(35)**,** 3924-3933. doi: 10.1200/jco.2017.74.3062.

Jotte, R., Cappuzzo, F., Vynnychenko, I., Stroyakovskiy, D., Rodriguez-Abreu, D., Hussein, M., et al. (2020). Atezolizumab in Combination With Carboplatin and Nab-Paclitaxel in Advanced Squamous Non-Small-Cell Lung Cancer (IMpower131): results From a Randomized Phase III Trial. *Journal of thoracic oncology*. doi: 10.1016/j.jtho.2020.03.028.

Kahl, K.L. (2020). Nivolumab/Ipilimumab Combo Yields Durable Efficacy in Advanced NSCLC. *Oncology (Williston Park)* 34(7)**,** 254.

Keeping, S.T., Cope, S., Chan, K., Wilson, F.R., Jansen, J.P., Penrod, J.R., et al. (2020). Comparative effectiveness of nivolumab versus standard of care for third-line patients with small-cell lung cancer. *J Comp Eff Res* 9(18)**,** 1275-1284. doi: 10.2217/cer-2020-0134.

Langer, C.J., Gadgeel, S.M., Borghaei, H., Papadimitrakopoulou, V.A., Patnaik, A., Powell, S.F., et al. (2016). Carboplatin and pemetrexed with or without pembrolizumab for advanced, non-squamous non-small-cell lung cancer: a randomised, phase 2 cohort of the open-label KEYNOTE-021 study. *Lancet Oncol* 17(11)**,** 1497-1508. doi: 10.1016/s1470-2045(16)30498-3.

Levy, B.P., Giaccone, G., Besse, B., Felip, E., Garassino, M.C., Domine Gomez, M., et al. (2019). Randomised phase 2 study of pembrolizumab plus CC-486 versus pembrolizumab plus placebo in patients with previously treated advanced non-small cell lung cancer. *Eur J Cancer* 108**,** 120-128. doi: 10.1016/j.ejca.2018.11.028.

Lu, S., Wang, J., Cheng, Y., Mok, T., Chang, J., Zhang, L., et al. (2020). Nivolumab versus docetaxel in a predominantly Chinese patient population with previously treated advanced non-small cell lung cancer: 2-year follow-up from a randomized, open-label, phase 3 study (CheckMate 078). *Lung Cancer* 152**,** 7-14. doi: 10.1016/j.lungcan.2020.11.013.

Lynch, T.J., Bondarenko, I., Luft, A., Serwatowski, P., Barlesi, F., Chacko, R., et al. (2012). Ipilimumab in combination with paclitaxel and carboplatin as first-line treatment in stage IIIB/IV non-small-cell lung cancer: results from a randomized, double-blind, multicenter phase II study. *J Clin Oncol* 30(17)**,** 2046-2054. doi: 10.1200/jco.2011.38.4032.

Mansfield, A.S., Każarnowicz, A., Karaseva, N., Sánchez, A., De Boer, R., Andric, Z., et al. (2020). Safety and patient-reported outcomes of atezolizumab, carboplatin, and etoposide in extensive-stage small-cell lung cancer (IMpower133): a randomized phase I/III trial. *Ann Oncol* 31(2)**,** 310-317. doi: 10.1016/j.annonc.2019.10.021.

Mazieres, J., Kowalski, D., Luft, A., Vicente, D., Tafreshi, A., Gümüş, M., et al. (2020). Health-Related Quality of Life With Carboplatin-Paclitaxel or nab-Paclitaxel With or Without Pembrolizumab in Patients With Metastatic Squamous Non-Small-Cell Lung Cancer. *J Clin Oncol* 38(3)**,** 271-280. doi: 10.1200/jco.19.01348.

Mazieres, J., Rittmeyer, A., Gadgeel, S., Hida, T., Gandara, D.R., Cortinovis, D.L., et al. (2021). Atezolizumab Versus Docetaxel in Pretreated Patients With NSCLC: Final Results From the Randomized Phase 2 POPLAR and Phase 3 OAK Clinical Trials. *J Thorac Oncol* 16(1)**,** 140-150. doi: 10.1016/j.jtho.2020.09.022.

Mok, T.S.K., Wu, Y.L., Kudaba, I., Kowalski, D.M., Cho, B.C., Turna, H.Z., et al. (2019). Pembrolizumab versus chemotherapy for previously untreated, PD-L1-expressing, locally advanced or metastatic non-small-cell lung cancer (KEYNOTE-042): a randomised, open-label, controlled, phase 3 trial. *Lancet* 393(10183)**,** 1819-1830. doi: 10.1016/s0140-6736(18)32409-7.

Nishio, M., Barlesi, F., West, H., Ball, S., Bordoni, R., Cobo, M., et al. (2020). Atezolizumab Plus Chemotherapy for First-Line Treatment of Non-Squamous Non-Small Cell Lung Cancer: Results From the Randomized Phase III IMpower132 Trial. *J Thorac Oncol*. doi: 10.1016/j.jtho.2020.11.025.

Nishio, M., Sugawara, S., Atagi, S., Akamatsu, H., Sakai, H., Okamoto, I., et al. (2019). Subgroup Analysis of Japanese Patients in a Phase III Study of Atezolizumab in Extensive-stage Small-cell Lung Cancer (IMpower133). *Clin Lung Cancer* 20(6)**,** 469-476.e461. doi: 10.1016/j.cllc.2019.07.005.

Owonikoko, T.K., Kim, H.R., Govindan, R., Ready, N., Reck, M., Peters, S., et al. (2019). Nivolumab (nivo) plus ipilimumab (ipi), nivo, or placebo (pbo) as maintenance therapy in patients (pts) with extensive disease small cell lung cancer (ED-SCLC) after first-line (1L) platinum-based chemotherapy (chemo): Results from the double-blind, randomized phase III CheckMate 451 study. *Annals of Oncology* 30(Suppl 2)**,** ii77. doi: 10.1093/annonc/mdz094.

Owonikoko, T.K., Park, K., Govindan, R., Ready, N., Reck, M., Peters, S., et al. (2021). Nivolumab and Ipilimumab as Maintenance Therapy in Extensive-Disease Small-Cell Lung Cancer: CheckMate 451. *J Clin Oncol***,** Jco2002212. doi: 10.1200/jco.20.02212.

Paz-Ares, L., Ciuleanu, T.E., Cobo, M., Schenker, M., Zurawski, B., Menezes, J., et al. (2021). First-line nivolumab plus ipilimumab combined with two cycles of chemotherapy in patients with non-small-cell lung cancer (CheckMate 9LA): an international, randomised, open-label, phase 3 trial. *Lancet Oncol* 22(2)**,** 198-211. doi: 10.1016/s1470-2045(20)30641-0.

Paz-Ares, L., Dvorkin, M., Chen, Y., Reinmuth, N., Hotta, K., Trukhin, D., et al. (2019). Durvalumab plus platinum-etoposide versus platinum-etoposide in first-line treatment of extensive-stage small-cell lung cancer (CASPIAN): a randomised, controlled, open-label, phase 3 trial. *Lancet* 394(10212)**,** 1929-1939. doi: 10.1016/s0140-6736(19)32222-6.

Paz-Ares, L., Luft, A., Vicente, D., Tafreshi, A., Gümüş, M., Mazières, J., et al. (2018). Pembrolizumab plus Chemotherapy for Squamous Non-Small-Cell Lung Cancer. *N Engl J Med* 379(21)**,** 2040-2051. doi: 10.1056/NEJMoa1810865.

Paz-Ares, L., Spira, A., Raben, D., Planchard, D., Cho, B.C., Özgüroğlu, M., et al. (2020a). Outcomes with durvalumab by tumour PD-L1 expression in unresectable, stage III non-small-cell lung cancer in the PACIFIC trial. *Ann Oncol*. doi: 10.1016/j.annonc.2020.03.287.

Paz-Ares, L., Vicente, D., Tafreshi, A., Robinson, A., Soto Parra, H., Mazières, J., et al. (2020b). A Randomized, Placebo-Controlled Trial of Pembrolizumab Plus Chemotherapy in Patients With Metastatic Squamous NSCLC: Protocol-Specified Final Analysis of KEYNOTE-407. *J Thorac Oncol* 15(10)**,** 1657-1669. doi: 10.1016/j.jtho.2020.06.015.

Planchard, D., Reinmuth, N., Orlov, S., Fischer, J.R., Sugawara, S., Mandziuk, S., et al. (2020). ARCTIC: durvalumab with or without tremelimumab as third-line or later treatment of metastatic non-small-cell lung cancer. *Ann Oncol* 31(5)**,** 609-618. doi: 10.1016/j.annonc.2020.02.006.

Pujol, J.L., Greillier, L., Audigier-Valette, C., Moro-Sibilot, D., Uwer, L., Hureaux, J., et al. (2019). A Randomized Non-Comparative Phase II Study of Anti-Programmed Cell Death-Ligand 1 Atezolizumab or Chemotherapy as Second-Line Therapy in Patients With Small Cell Lung Cancer: Results From the IFCT-1603 Trial. *J Thorac Oncol* 14(5)**,** 903-913. doi: 10.1016/j.jtho.2019.01.008.

Ready, N.E., Ott, P.A., Hellmann, M.D., Zugazagoitia, J., Hann, C.L., de Braud, F., et al. (2020). Nivolumab Monotherapy and Nivolumab Plus Ipilimumab in Recurrent Small Cell Lung Cancer: Results From the CheckMate 032 Randomized Cohort. *J Thorac Oncol* 15(3)**,** 426-435. doi: 10.1016/j.jtho.2019.10.004.

Reck, M., Bondarenko, I., Luft, A., Serwatowski, P., Barlesi, F., Chacko, R., et al. (2013). Ipilimumab in combination with paclitaxel and carboplatin as first-line therapy in extensive-disease-small-cell lung cancer: results from a randomized, double-blind, multicenter phase 2 trial. *Ann Oncol* 24(1)**,** 75-83. doi: 10.1093/annonc/mds213.

Reck, M., Brahmer, J., Bennett, B., Taylor, F., Penrod, J.R., DeRosa, M., et al. (2018a). Evaluation of health-related quality of life and symptoms in patients with advanced non-squamous non-small cell lung cancer treated with nivolumab or docetaxel in CheckMate 057. *Eur J Cancer* 102**,** 23-30. doi: 10.1016/j.ejca.2018.05.005.

Reck, M., Luft, A., Szczesna, A., Havel, L., Kim, S.W., Akerley, W., et al. (2016a). Phase III Randomized Trial of Ipilimumab Plus Etoposide and Platinum Versus Placebo Plus Etoposide and Platinum in Extensive-Stage Small-Cell Lung Cancer. *J Clin Oncol* 34(31)**,** 3740-3748. doi: 10.1200/jco.2016.67.6601.

Reck, M., Rodríguez-Abreu, D., Robinson, A.G., Hui, R., Csőszi, T., Fülöp, A., et al. (2016b). Pembrolizumab versus Chemotherapy for PD-L1-Positive Non-Small-Cell Lung Cancer. *N Engl J Med* 375(19)**,** 1823-1833. doi: 10.1056/NEJMoa1606774.

Reck, M., Rodríguez-Abreu, D., Robinson, A.G., Hui, R., Csőszi, T., Fülöp, A., et al. (2019a). Updated Analysis of KEYNOTE-024: Pembrolizumab Versus Platinum-Based Chemotherapy for Advanced Non-Small-Cell Lung Cancer With PD-L1 Tumor Proportion Score of 50% or Greater. *J Clin Oncol* 37(7)**,** 537-546. doi: 10.1200/jco.18.00149.

Reck, M., Schenker, M., Lee, K.H., Provencio, M., Nishio, M., Lesniewski-Kmak, K., et al. (2019b). Nivolumab plus ipilimumab versus chemotherapy as first-line treatment in advanced non-small-cell lung cancer with high tumour mutational burden: patient-reported outcomes results from the randomised, open-label, phase III CheckMate 227 trial. *Eur J Cancer* 116**,** 137-147. doi: 10.1016/j.ejca.2019.05.008.

Reck, M., Taylor, F., Penrod, J.R., DeRosa, M., Morrissey, L., Dastani, H., et al. (2018b). Impact of Nivolumab versus Docetaxel on Health-Related Quality of Life and Symptoms in Patients with Advanced Squamous Non-Small Cell Lung Cancer: Results from the CheckMate 017 Study. *J Thorac Oncol* 13(2)**,** 194-204. doi: 10.1016/j.jtho.2017.10.029.

Rittmeyer, A., Barlesi, F., Waterkamp, D., Park, K., Ciardiello, F., von Pawel, J., et al. (2017). Atezolizumab versus docetaxel in patients with previously treated non-small-cell lung cancer (OAK): a phase 3, open-label, multicentre randomised controlled trial. *Lancet* 389(10066)**,** 255-265. doi: 10.1016/s0140-6736(16)32517-x.

Rizvi, N.A., Cho, B.C., Reinmuth, N., Lee, K.H., Luft, A., Ahn, M.J., et al. (2020). Durvalumab With or Without Tremelimumab vs Standard Chemotherapy in First-line Treatment of Metastatic Non-Small Cell Lung Cancer: The MYSTIC Phase 3 Randomized Clinical Trial. *JAMA Oncol* 6(5)**,** 661-674. doi: 10.1001/jamaoncol.2020.0237.

Rudin, C.M., Awad, M.M., Navarro, A., Gottfried, M., Peters, S., Csőszi, T., et al. (2020). Pembrolizumab or Placebo Plus Etoposide and Platinum as First-Line Therapy for Extensive-Stage Small-Cell Lung Cancer: Randomized, Double-Blind, Phase III KEYNOTE-604 Study. *J Clin Oncol* 38(21)**,** 2369-2379. doi: 10.1016/j.lungcan.2019.06.007

10.1200/jco.20.00793.

Satouchi, M. (2020). First-line pembrolizumab vs chemotherapy in metastatic non-small-cell lung cancer: KEYNOTE-024 Japan subset. *BMC Cancer* 111(12)**,** 4480-4489. doi: 10.1186/s12885-020-06958-3

10.1111/cas.14647.

Sezer, A., Kilickap, S., Gümüş, M., Bondarenko, I., Özgüroğlu, M., Gogishvili, M., et al. (2021). Cemiplimab monotherapy for first-line treatment of advanced non-small-cell lung cancer with PD-L1 of at least 50%: a multicentre, open-label, global, phase 3, randomised, controlled trial. *Lancet* 397(10274)**,** 592-604. doi: 10.1016/s0140-6736(21)00228-2.

Socinski, M.A., Jotte, R.M., Cappuzzo, F., Orlandi, F., Stroyakovskiy, D., Nogami, N., et al. (2018). Atezolizumab for First-Line Treatment of Metastatic Nonsquamous NSCLC. *N Engl J Med* 378(24)**,** 2288-2301. doi: 10.1056/NEJMoa1716948.

Spigel, D., Jotte, R., Nemunaitis, J., Shum, M., Schneider, J., Goldschmidt, J., et al. (2020). Randomized Phase 2 Studies of Checkpoint Inhibitors Alone or in Combination With Pegilodecakin in Patients With Metastatic NSCLC (CYPRESS 1 and CYPRESS 2). *J Thorac Oncol*. doi: 10.1016/j.jtho.2020.10.001.

Spigel, D.R., Vicente, D., Ciuleanu, T.E., Gettinger, S., Peters, S., Horn, L., et al. (2021). Second-Line Nivolumab in Relapsed Small-Cell Lung Cancer: CheckMate 331. *Ann Oncol*. doi: 10.1016/j.annonc.2021.01.071.

Vokes, E.E., Ready, N., Felip, E., Horn, L., Burgio, M.A., Antonia, S.J., et al. (2018). Nivolumab versus docetaxel in previously treated advanced non-small-cell lung cancer (CheckMate 017 and CheckMate 057): 3-year update and outcomes in patients with liver metastases. *Ann Oncol* 29(4)**,** 959-965. doi: 10.1093/annonc/mdy041.

von Pawel, J., Bordoni, R., Satouchi, M., Fehrenbacher, L., Cobo, M., Han, J.Y., et al. (2019). Long-term survival in patients with advanced non–small-cell lung cancer treated with atezolizumab versus docetaxel: Results from the randomised phase III OAK study. *European Journal of Cancer* 107**,** 124-132. doi: 10.1016/j.ejca.2018.11.020.

Wang, J., Lu, S., Yu, X., Hu, Y., Sun, Y., Wang, Z., et al. (2021). Tislelizumab Plus Chemotherapy vs Chemotherapy Alone as First-line Treatment for Advanced Squamous Non-Small-Cell Lung Cancer: A Phase 3 Randomized Clinical Trial. *JAMA Oncol*. doi: 10.1001/jamaoncol.2021.0366.

West, H., McCleod, M., Hussein, M., Morabito, A., Rittmeyer, A., Conter, H.J., et al. (2019). Atezolizumab in combination with carboplatin plus nab-paclitaxel chemotherapy compared with chemotherapy alone as first-line treatment for metastatic non-squamous non-small-cell lung cancer (IMpower130): a multicentre, randomised, open-label, phase 3 trial. *Lancet Oncol* 20(7)**,** 924-937. doi: 10.1016/s1470-2045(19)30167-6.

Wu, Y.L., Lu, S., Cheng, Y., Zhou, C., Wang, J., Mok, T., et al. (2019). Nivolumab Versus Docetaxel in a Predominantly Chinese Patient Population With Previously Treated Advanced NSCLC: CheckMate 078 Randomized Phase III Clinical Trial. *J Thorac Oncol* 14(5)**,** 867-875. doi: 10.1016/j.jtho.2019.01.006.

Wu, Y.L., Zhang, L., Fan, Y., Zhou, J., Zhang, L., Zhou, Q., et al. (2020). Randomized clinical trial of pembrolizumab vs chemotherapy for previously untreated Chinese patients with PD-L1-positive locally advanced or metastatic non-small-cell lung cancer: KEYNOTE-042 China Study. *Int J Cancer*. doi: 10.1002/ijc.33399.

Yang, Y., Wang, Z., Fang, J., Yu, Q., Han, B., Cang, S., et al. (2020). Efficacy and Safety of Sintilimab Plus Pemetrexed and Platinum as First-Line Treatment for Locally Advanced or Metastatic Nonsquamous NSCLC: a Randomized, Double-Blind, Phase 3 Study (Oncology pRogram by InnovENT anti-PD-1-11). *J Thorac Oncol* 15(10)**,** 1636-1646. doi: 10.1007/s00262-020-02738-x

10.1016/j.jtho.2020.07.014.

Zhou, C., Chen, G., Huang, Y., Zhou, J., Lin, L., Feng, J., et al. (2020). Camrelizumab plus carboplatin and pemetrexed versus chemotherapy alone in chemotherapy-naive patients with advanced non-squamous non-small-cell lung cancer (CameL): a randomised, open-label, multicentre, phase 3 trial. *Lancet Respir Med*. doi: 10.1080/15384047.2020.1829265

10.1016/s2213-2600(20)30365-9.
